# Supplementary material for: Understanding the relation between Zika virus infection during pregnancy and adverse fetal, infant and child outcomes: a protocol for a systematic review and individual participant data meta-analysis of longitudinal studies of pregnant women and their infants and children
Source: BMJ Open. 2019 Jun 18;9(6):e026092. doi: 10.1136/bmjopen-2018-026092 (PMC6588966; doi:10.1136/bmjopen-2018-026092)
Supplement: Supplementary data [file bmjopen-2018-026092supp001.pdf]

Supplementary Table 1. List of longitudinal research studies and active surveillance programs that have agreed to contribute participant-level data to the ZIKV Consortium individual participant data meta-analysis of longitudinal studies of pregnant women and their infants and children

Country	City	Study Name	Coordinating Center(s)	Consortium Name*
Brazil	Campina Grande	Freqüência e evolução dos achados ultrassonograficos e de ressonäncia magnética em fetos de mães com sintomas de Zika virus e a associação com desfechos neonatais em Campina Grande - Paraíba: Estudo de coorte	Instituto do Cérebro, Rio de Janeiro; Instituto D´Or, Rio de Janeiro	
Brazil	Goiânia	Cohort of Pregnant women with rash from Goiânia, Goiás State, Brazil and Cohort of children vertically exposed to Zika virus in Goiania	Institute of Tropical Pathology and Public Health<br><br>Federal University of Goiás, Brazil	ZikaPLAN
Brazil	Jundiaí	Infecção Vertical pelo vírus ZIKA e suas repercussões na área materno-infantil	Faculdade de Medicina de Jundiaí	
Brazil	São Luís, Maranhão	Monitoramento da microcefalia em recém-nascidos e acompanhamento clínico e de crescimento e desenvolvimento de uma coorte de crianças com provável infecção congênita pelo virus da Zika	Hospital Universitário da Universidade Federal do Maranhão/HU/UFMA	

Country	City	Study Name	Coordinating Center(s)	Consortium Name*
Brazil	Metropolitan region of Recife, Pernambuco	Coorte de gestantes com exantema no estado de Pernambuco	Universidade Federal de Pernambuco and Centro de Pesquisas Aggeu Magalhães-Fiocruz-PE	MERG/Fiocruz, ZikaPLAN
Brazil	Pernambuco	Coorte de gestanes com exantema no estado de Pernambuco	Fundação Oswaldo Cruz (Fiocruz)	MERG/Fiocruz, ZikaPlan
Brazil	Pernambuco	Coorte clínica de crianças com microcefalia em Pernambuco	Universidade Federal de Pernambuco and Centro de Pesquisas Aggeu Magalhães-Fiocruz-PE	MERG/Fiocruz, ZikaPlan
Brazil	Ribeirão Preto	Natural history of Zika virus infection in pregnant and consequences for pregnancy, fetus and child (Zika Project in Pregnancy - ZIG)	Universidade de São Paulo	
Brazil	Rio de Janeiro	Infecção pelo vírus Zika em uma coorte de gestantes e seus conceptos	Maternidade Escola da Universidade Federal do Rio de Janeiro	
Brazil	Rio de Janeiro	Estudo de coorte de gestantes e criancas expostas e infectadas intrautero pelo Zika virus	Instituto de Puericultura e Pediatria Martagão Gesteira, Rio de Janeiro; Hospital Universitário Pedro Ernesto	
Brazil	Rio de Janeiro	Zika Virus Infection in Pregnant Women in Rio de Janeiro	Fundação Oswaldo Cruz (Fiocruz), Rio de Janeiro	Fiocruz
Brazil	Rio de Janeiro	Zika virus coinfection among HIV infected pregnant women in a Brazilian cohort	Hospital dos Servidores do Estado	

Country	City	Study Name	Coordinating Center(s)	Consortium Name*
Brazil	São José do Rio Preto	Diagnóstico de arboviroses brasileiras e emergentes em pacientes e mosquitos em duas regiões distintas do Brasil	Faculdade de Medicina de São José do Rio Preto, Secretaria de Desenvolvimento, Econômico, Ciência e Tecnologia, São Paulo State	
Brazil	Vitoria	Epidemia de Zika virus no estado do Espirito Santo: estudo de impacto da infeccao sobre o feto em uma coorte de gestantes, com sintomas da doenca e confirmacao virologica da infeccao	Hospital Universitário Cassiano Antônio de Moraes	
Brazil<br>Colombia<br>Guatemala<br>Nicaragua<br>Puerto Rico<br>Mexico		Zika in Infants and Pregnancy (ZIP)	RTI International; Eunice Kennedy Shriver National Institute of Child Health and Human Development; National Institute of Allergy and Infectious Disease, National Institute of Environmental Health Sciences; Fundação Oswaldo Cruz (Fiocruz)	NIH/NIAID
Colombia	Baranquilla, Soledad, Bucaramanga, Tuluá	Zika en Embarazadas y Niños (ZEN)		CDC/INS
Colombia	Santander	Neurodevelopment outcome of newborns exposed to Zika virus in utero (ZEN)	UNC-CH, Michigan State University, Universidad Industrial de Santander	

Country	City	Study Name	Coordinating Center(s)	Consortium Name*
Colombia	Barranquilla<br>Cali<br>Cúcuta	Vigilancia de Embarazadas con Zika (VEZ; Surveillance cohort)		CDC
Ecuador<br>Cuba<br>Mexico (IMSS, MOH)<br>Venezuela: Valencia<br>Brazil: Fortaleza, Recife, Rio de Janeiro<br>Colombia: Bucaramanga		Pregnant Women Cohort for evaluation of absolute and relative risk of congenital malformations after Zika virus infection – developmental milestones of children born to women exposed to Zika virus during pregnancy	Heidelberg University	ZIKAlliance, Fiocruz, IDAMS
Grenada		The Spectrum of Zika Disease in Grenada - Pregnancy Cohort	St. George's University, Stanford University, Windward Islands Research and Education Foundation	
Guadaloupe, Martinique, French Guyana, St Martin		Zika Virus Infection's Pregnancy Consequences in French Department of America (ZIKA-DFA-FE)		INSERM
French Guyana		Zika Virus Infection's Neonatal and Pediatric Consequences in French Department of America (ZIKA-DFA-BB)		INSERM
Honduras		Zika Virus Infection in Pregnant Women in Honduras (ZIPH case-cohort study)	Tulane	
La Réunion		ZikaRun: an integrative mother-infant inception cohort study to anticipate	Cellule Régionale de l'Institut de Veille Sanitaire océan Indien	INSERM

Country	City	Study Name	Coordinating Center(s)	Consortium Name*
		the introduction of Zika virus in the at-risk La Reunion island, Indian Ocean	2Département de Médecine Générale, UFR santé, Université de la Réunion, Saint Denis 3INSERM CIC1410, CHU Reunion, Saint Denis - Saint Pierre 4CH Gabriel Martin, Saint Paul 5Centre d'Etudes Périnatales de l'océan Indien (CEPOI), EA7388, Université de la Réunion, CHU Reunion, Saint Pierre 6UM 134 Processus Infectieux en Milieu Insulaire Tropical (PIMIT), Université de La Réunion, INSERM U1187, CNRS 9192, IRD 249 7UMR Diabète AthéroThRombose Océan Indien (DéTROI), INSERM U188, Sainte Clotilde, La Réunion"	
Jamaica, Haiti		ZIKAction: Mother to child transmission of Chikungunya, Dengue, and Zika Virus Infection: A prospective observational cohort study of pregnant women and their infants		ZIKAction

Country	City	Study Name	Coordinating Center(s)	Consortium Name*
Panama<br>El Salvador		Panama/El Salvador Influenza Birth Cohort Study with Added Zika Component		CDC
Spain		pedZIKARed/gestZIKARed Spanish Zika database for pregnant women and children	Barceola University Hospital Vall d'Hebron	ZIKAction
Suriname		A symptomatic cohort study in Zika infected pregnant women	Acadamic Hospital Paramaribo	
Western French Guiana		Association between Zika virus and foetopathy: a prospective cohort study in French Guiana	Centre Hospitalier de l'Ouest Guyanais Saint-Laurent du Maroni	

CDC=Centers for Disease Control and Prevention; IDAMS=International Research Consortium on Dengue Risk Assessment, Management, and Surveillance; INSERM=Institut National de la Santé Et de la Recherche Médicale; NIAID=National Institutes of Allergy and Infectious Disease; NIH=National Institutes of Health
