# Supplementary material for: Understanding the relation between Zika virus infection during pregnancy and adverse fetal, infant and child outcomes: a protocol for a systematic review and individual participant data meta-analysis of longitudinal studies of pregnant women and their infants and children
Source: BMJ Open. 2019 Jun 18;9(6):e026092. doi: 10.1136/bmjopen-2018-026092 (PMC6588966; doi:10.1136/bmjopen-2018-026092)
Supplement: Supplementary data [file bmjopen-2018-026092supp003.pdf]

Supplementary Table 3. Zika virus-related and general clinical trial databases (adapted from Reveiz, et al [1])

Data base name	Link
Clinical Trails.gov	https://clinicaltrials.gov/ct2/search
World Health Organization (WHO) International Clinical Trials Registry Platform (ICTRP)	http://apps.who.int/trialsearch/
United States Centers for Disease Control and Prevention (US-CDC)	https://www.cdc.gov/publications/
European Centers for Disease Control (E-CDC)	https://ecdc.europa.eu/en/publications-data
Pan American Health Organization (PAHO) Zika research portal	https://www.paho.org/zika-research/
Fiocruz Research portal	https://portal.fiocruz.br/
Sistema Nacional de Ética em Pesquisa (SISNEP)	http://portal2.saude.gov.br/sisnep/pesquisador/
Registro peruano de ensayos clínicos y de estudios observacionales (REPEC)	http://www.ensayosclinicos-repec.ins.gob.pe/acerca-del-repec/busqueda-de-ensayos-clinicos
Registro nacional de investigaciones en salud (ReNIS)	https://sisa.msal.gov.ar/sisa/#Renis
Registro nacional de ensayos clínicos (RNEC)	http://189.254.115.252/Resoluciones/Consultas/ConWebRegEnsayosClinicos.asp

Reference

1. Reveiz L, Haby MM, Martínez-Vega R, Pinzón-Flores CE, Elias V, Smith E, et al. Risk of bias and confounding of observational studies of Zika virus infection: A scoping review of research protocols. PLOS ONE. 2017;12(7):e0180220. doi: 10.1371/journal.pone.0180220.
