# Supplementary material for: Understanding the relation between Zika virus infection during pregnancy and adverse fetal, infant and child outcomes: a protocol for a systematic review and individual participant data meta-analysis of longitudinal studies of pregnant women and their infants and children
Source: BMJ Open. 2019 Jun 18;9(6):e026092. doi: 10.1136/bmjopen-2018-026092 (PMC6588966; doi:10.1136/bmjopen-2018-026092)
Supplement: Supplementary data [file bmjopen-2018-026092supp004.pdf]

Supplementary Text 1. ZIKV IPD-MA search strategy

PICO Question:

Population	Exposure	Comparator	Outcome (open)
Pregnant women and her fetus, infant, or child	ZIKV infection during pregnancy	No ZIKV infection during pregnancy	Primary: microcephaly, miscarriage, fetal loss. Secondary: early/late fetal death, ocular abnormalities, hearing loss, neuroimaging abnormalities, etc.

Medline (through Ovid):

1. exp Zika Virus Infection/ or exp ZIKA VIRUS/

2. (zika or ZIKV).ti,ab,kf.

3. 1 or 2

4. exp Pregnancy/ or exp Maternal Exposure/ or exp "Embryonic and Fetal Development"/ or exp "Congenital, Hereditary, and Neonatal Diseases and Abnormalities"/ or exp Infant/ or exp Child/

5. (pregnan* or matern* or gestation* or perinatal* or birth* or congenital* or newborn* or fetal or fetus* or foetal or foetus* or neonat* or infan* or toddler* or child*).ti,ab,kf.

6. 4 or 5

7. 3 and 6

8. 7 not (exp Animals/ not exp Humans/)

Embase (through Ovid):

1. exp Zika virus/ or exp Zika fever/

2. (zika or ZIKV).ti,ab,kw.

3. 1 or 2

4. exp pregnancy/ or exp pregnancy outcome/ or exp high risk pregnancy/ or exp pregnancy complication/ or exp maternal exposure/ or exp fetus/ or exp "functions of embryonic, fetal and placental structures"/ or exp Infant/ or exp infant disease/ or exp child/ or exp childhood disease/

5. (pregnan* or matern* or gestation* or perinatal* or birth* or congenital* or newborn* or fetal or fetus* or foetal or foetus* or neonat* or infan* or toddler* or child*).ti,ab,kw.

6. 4 or 5

7. 3 and 6

8. 7 not ((exp animal/ or exp nonhuman/) not exp human/)
