# Supplementary material for: Understanding the relation between Zika virus infection during pregnancy and adverse fetal, infant and child outcomes: a protocol for a systematic review and individual participant data meta-analysis of longitudinal studies of pregnant women and their infants and children
Source: BMJ Open. 2019 Jun 18;9(6):e026092. doi: 10.1136/bmjopen-2018-026092 (PMC6588966; doi:10.1136/bmjopen-2018-026092)
Supplement: Author's manuscript [file bmjopen-2018-026092.draft_revisions.pdf]

BMJ Open is committed to open peer review. As part of this commitment we make the peer review history of every article we publish publicly available.

When an article is published we post the peer reviewers' comments and the authors' responses online. We also post the versions of the paper that were used during peer review. These are the versions that the peer review comments apply to.

The versions of the paper that follow are the versions that were submitted during the peer review process. They are not the versions of record or the final published versions. They should not be cited or distributed as the published version of this manuscript.

BMJ Open is an open access journal and the full, final, typeset and author-corrected version of record of the manuscript is available on our site with no access controls, subscription charges or pay-per-view fees (http://bmjopen.bmj.com).

If you have any questions on BMJ Open's open peer review process please email editorial.bmjopen@bmj.com

BMJ Open

Understanding the relation between Zika virus infection during pregnancy and adverse fetal, infant, and child outcomes: a protocol for a systematic review and individual participant data meta-analysis of longitudinal studies of pregnant women and their infants and children

Journal:	*BMJ Open*
Manuscript ID	bmjopen-2018-026092
Article Type:	Protocol
Date Submitted by the Author:	17-Aug-2018

Complete List of Authors:	Wilder-Smith, Annelies; Nanyang Technological University, Lee Kong Chian School of Medicine<br>Wei, Yinghui; University of Plymouth, Centre for Mathematical Sciences<br>Velho Barreto de Araújo, Thalia; Universidade Federal de Pernambuco, Department of Social Medicine<br>Turchi Martelli, Celina Maria; Oswaldo Cruz Foundation , Department of Collective Health, Institute Aggeu Magalhães (CPqAM)<br>Turchi, Marília Dalva; Federal University of Goias, Institute of Tropical Pathology and Public Health<br>Tami, Adriana; University Medical Center Groningen, Department of Medical Microbiology<br>Souza, João ; University of São Paulo<br>Sousa, Patricia; State Department of Health of Maranhão, Reference Center for Neurodevelopment, Assistance, and Rehabilitation of Children<br>Soriano-Arandes, Antoni; University Hospital Vall d'Hebron, Department of Pediatrics<br>Sanchez Clemente, Nuria; University of São Paulo, Department of Epidemiology<br>Reveiz, L; Pan American Health Organization, Evidence and Intelligence for Action in Health<br>Prata-Barbosa, Arnaldo; D'Or Institute for Research & Education, Department of Pediatrics<br>Pomar, Léo; Centre Hospitalier de l'Ouest Guyanais, Department of Obstetrics and Gynecology<br>Pelá Rosado, Luiza Emylce ; Goiânia State Health Secretary, Hospital Materno Infantil de Goiânia<br>Perez, Freddy; Pan American Health Organization, Communicable Diseases and Environmental Determinants of Health Department<br>Passos, Saulo; FMJ, Department of Pediatrics<br>Nogueira, Mauricio; Faculdade de Medicina de Sao Jose do Rio Preto, Department of Dermatologic Diseases<br>Noel, Trevor P.; St. George's University, Windward Islands Research and Education Foundation<br>Moura da Silva , Antônio ; Universidade Federal do Maranhão - São Luís , Department of Public Health<br>Moreira , Maria Elisabeth; Oswaldo Cruz Foundation (Fiocruz), Department of Neonatology

Miranda Montoya, Maria Consuelo ; Universidad Industrial de Santander, Facultad de Salud Carrera
Miranda-Filho, Demócrito de Barros; University of Pernambuco, Faculty of Medical Sciences
Maxwell, Lauren; World Health Organization, Reproductive Health and Research; Emory University, Hubert Department of Global Health
Macpherson, Calum; St George's University, Windward Islands Research and Education Foundation
Low, Nicola; University of Bern, Bern, Switzerland, Institute of Social and Preventive Medicine
Lan , Zhiyi; McGill University, McGill University Health Centre
LaBeaud, Angelle Desiree ; Stanford Hospital, Pediatric Infectious Diseases
Koopmans, M; Erasmus Medical Center, Rotterdam, Netherlands, Department of Virology
Kim, Caron; World Health Organization, Department of Reproductive Health and Research
João, Esaú; Hospital Federal dos Servidores do Estado, Department of Infectious Diseases
Jaenisch, Thomas ; UniversitatsKlinikum Heidelberg, Department of Infectious Diseases, Section Clinical Tropical Medicine;
Hofer, C. B.; Universidade Federal do Rio de Janeiro, Instituto de Puericultura e Pediatria Martagão Gesteira
Gustafson, Paul; University of British Columbia, Statistics
Gérardin, Patrick; CHU La Réunion, INSERM CIC1410 Clinical Epidemiology; Universite de la Reunion, UM 134 PIMIT (CNRS 9192, INSERM U1187, IRD 249, Université de la Réunion)
Ganz, Jucelia S; Children’s Hospital Juvencio Matos
Elias, Vanessa; Pan American Health Organization, Sustainable Development and Environmental Health
Debray, Thomas; University Medical Center Utrecht, Julius Center for Health Sciences and Primary Care
Cafferata, Maria Luisa; Instituto de Efectividad Clinica y Sanitaria, Mother and Children Health Research Department
buekens, pierre; Tulane University, School of Public Health and Tropical Medicine
Broutet, Nathalie; World Health Organization, Dept of Reproductive Health and Research
Brickley, Elizabeth B.; London School of Hygiene and Tropical Medicine, Department of Infectious Disease Epidemiology
Brasil, Patrícia ; Fundacao Oswaldo Cruz, Instituto de pesquisa Clínica Evandro Chagas
Bethencourt, Sarah ; Universidad de Carabobo, Facultad de Ciencias de la Salud
Benedetti, Andrea; McGill University, Departments of Medicine and of Epidemiology, Biostatistics & Occupational Health
Arraes de Alencar Ximenes, Ricardo; Federal University of Pernambuco, Department of Tropical Medicine
Alves da Cunha, Antonio; Federal University of Rio de Janeiro, Department of Pediatrics
Alger, Jackeline; Universidad Nacional Autónoma de Honduras, Facultad de Ciencias Médicas

Keywords: individual participant data meta-analysisis, prognosis, congenital Zika syndrome, Zika virus, microcephaly, risk prediction model

SCHOLARONE™
Manuscripts

For peer review only

TITLE: Understanding the relation between Zika virus infection during pregnancy and adverse fetal, infant, and child outcomes: a protocol for a systematic review and individual participant data meta-analysis of longitudinal studies of pregnant women and their infants and children

Registration: PROSPERO international prospective register of systematic reviews record number (CRD42017068915)

Author:
Zika Virus Individual Participant Data Consortium

Individual authors listed in descending alphabetical order at the end of the publication:
Annelies Wilder-Smith
Lee Kong Chian School of Medicine
Nanyang Technological University
Singapore

Yinghui Wei
Centre for Mathematical Sciences
University of Plymouth
Plymouth, England

Thalia Velho Barreto de Araújo
Department of Social Medicine
Federal University of Pernambuco
Recife, Brazil

Celina Maria Turchi Martelli
Department of Collective Health,
Institute Aggeu *Magalhães (CPqAM)*
Fundação Oswaldo Cruz (Fiocruz)
Recife, Brazil

Marília Dalva Turchi
Institute of Tropical Pathology and Public Health
Federal University of Goias
Goiânia, Brazil

Adriana Tami
Department of Medical Microbiology
University Medical Center Groningen
University of Groningen
Groningen, Netherlands

Patricia Sousa

For peer review only - http://bmjopen.bmj.com/site/about/guidelines.xhtml

Reference Center for Neurodevelopment, Assistance, and Rehabilitation of Children
State Department of Health of Maranhão
São Luís, Brazil

João Paulo Souza
Department of Social Medicine
University of São Paulo
São Paulo, Brazil

Antoni Soriano-Arandes
Department of Pediatrics
University Hospital Vall d'Hebron
Barcelona, Spain

Antonio A. Silva
Department of Public Health
Federal University of Maranhão
São Luís, Brazil

Nuria Sanchez Clemente
Department of Epidemiology
University of São Paulo
São Paulo, Brazil

Ludovic Reveiz
Evidence and Intelligence for Action in Health
Pan American Health Organization
Washington, D.C., USA

Arnaldo Prata-Barbosa
Department of Pediatrics
D'Or Institute for Research & Education
Rio de Janeiro, Brazil

Léo Pomar
Department of Obstetrics and Gynecology
Centre Hospitalier de l'Ouest Guyanais
Saint-Laurent du Maroni, French Guiana

Luiza Emylce Pelá Rosado
Hospital Materno Infantil de Goiânia
Goiânia State Health Secretary
Goiás, Brazil

For peer review only
For peer review only - http://bmjopen.bmj.com/site/about/guidelines.xhtml

Freddy Perez
Communicable Diseases and Environmental Determinants of Health Department
Pan American Health Organization
Washington, D.C., USA

Saulo Passos
Department of Pediatrics
Faculty of Medicine of Jundiai
São Paulo, Brazil

Mauricio Nogueira
Department of Dermatologic Diseases
Faculdade de Medicina de São José do Rio Preto
São José do Rio Preto, Brazil

Trevor P. Noel
Windward Islands Research and Education Foundation
St. George's University
True Blue Point, Grenada

Maria Elisabeth Moreira
Department of Neonatology
Fundação Oswaldo Cruz (Fiocruz)
Rio de Janeiro, Brazil

Demócrito de Barros Miranda-Filho
Faculty of Medical Sciences
University of Pernambuco
Recife, Brazil

María Consuelo Miranda Montoya
Facultad de Salud Carrera
Universidad Industrial de Santander
Santander, Colombia

*corresponding author
Lauren Maxwell
Department of Reproductive Health and Research
World Health Organization
Geneva, Switzerland
maxwelll@who.int
404.728.2017

Calum N.L. Macpherson
Windward Islands Research and Education Foundation

For peer review only - http://bmjopen.bmj.com/site/about/guidelines.xhtml

St. George's University
True Blue Point, Grenada

Nicola Low
Institute of Social and Preventive Medicine
University of Bern
Bern, Switzerland

Zhiyi Lan
McGill University Health Centre
McGill University
Montréal, Canada

Angelle Desiree LaBeaud
Pediatric Infectious Diseases
Stanford Hospital
California, USA

Marion Koopmans
Department of Virology
Erasmus MC
Rotterdam, Netherlands

Caron Kim
Department of Reproductive Health and Research
World Health Organization
Geneva, Switzerland

Esaú João
Department of Infectious Diseases
Hospital Federal dos Servidores do Estado
Rio de Janeiro, Brazil

Thomas Jaenisch
Department for Infectious Disease
Heidelberg University Hospital
Heidelberg, Germany

Cristina Hofer
Instituto de Puericultura e Pediatria Martagão Gesteira
Universidade Federal do Rio de Janeiro
Rio de Janeiro, Brazil

Paul Gustafson

For peer review only

For peer review only - http://bmjopen.bmj.com/site/about/guidelines.xhtml

Department of Statistics
University of British Columbia
Vancouver, Canada

Patrick Gérardin
Centre for Clinical Investigation (CIC1410)
Centre Hospitalier Universitaire de La Réunion
Saint Pierre, Réunion, France

Jucelia S. Ganz
Children's Hospital Juvencio Matos
São Luís, Brazil

Vanessa Elias
Sustainable Development and Environmental Health
Pan American Health Organization
Washington, D.C., USA

Thomas Paul Alfons Debray
Department of Epidemiology
University Medical Center Utrecht
Utrecht, Netherlands

María Luisa Cafferata
Mother and Children Health Research Department
Institute for Clinical Effectiveness and Health Policy
Buenos Aires, Argentina

Pierre Buekens
School of Public Health and Tropical Medicine
Tulane University
Louisiana, USA

Nathalie Broutet
Department of Reproductive Health and Research
World Health Organization
Geneva, Switzerland

Elizabeth B. Brickely
Department of Infectious Disease Epidemiology
London School of Hygiene and Tropical Medicine
London, England

Patrícia Brasil
Fundação Oswaldo Cruz

For peer review only

For peer review only - http://bmjopen.bmj.com/site/about/guidelines.xhtml

Instituto de pesquisa Clínica Evandro Chagas
Rio de Janeiro, Brazil

Sarah Bethencourt
Facultad de Ciencias de la Salud
Universidad de Carabobo
Valencia, Venezuela

Andrea Benedetti
McGill University Health Centre
McGill University
Montréal, Canada

Ricardo Arraes de Alencar Ximenes
Department of Tropical Medicine
Federal University of Pernambuco
Recife, Brazil

Antonio Alves da Cunha
Department of Pediatrics
Federal University of Rio de Janeiro
Rio de Janeiro, Brazil

Jackeline Alger
Facultad de Ciencias Médicas
Universidad Nacional Autónoma de Honduras
Tegucigalpa, Honduras

Word Count: 7701

Abstract

Introduction

Zika virus (ZIKV) infection during pregnancy is a known cause of microcephaly and other congenital and developmental anomalies. In the absence of a ZIKV vaccine or prophylactics, principal investigators (PIs) and international leaders in ZIKV research have formed the ZIKV Individual Participant Data (IPD) Consortium to identify, collect, and synthesize IPD from longitudinal studies of pregnant women that measure ZIKV infection during pregnancy and fetal, infant, or child outcomes.

Methods and analysis

We will identify eligible studies through the ZIKV IPD Consortium membership and a systematic review and invite study PIs to participate in the IPD-MA. We will use the combined dataset to estimate the relative and absolute risk of congenital Zika syndrome (CZS), including microcephaly and late symptomatic congenital infections; identify and explore sources of heterogeneity in those estimates;

For peer review only - http://bmjopen.bmj.com/site/about/guidelines.xhtml

and develop and validate a risk prediction model to identify the pregnancies at the highest risk of CZS or adverse developmental outcomes. The variable accuracy of diagnostic assays and differences in exposure and outcome definitions means that included studies will have a higher level of systematic variability, a component of measurement error, than an IPD-MA of studies of an established pathogen. We will use expert testimony, existing internal and external diagnostic accuracy validation studies, and laboratory external quality assessments to inform the distribution of measurement error in our models. We will apply both Bayesian and frequentist methods to directly account for these and other sources of uncertainty.

Ethics and dissemination
The IPD-MA was deemed exempt from ethical review. We will convene a group of patient advocates to evaluate the ethical implications and utility of the risk stratification tool. Findings from these analyses will be shared via national and international conferences and through publication in open access, peer-reviewed journals.

Registration: PROSPERO International prospective register of systematic reviews (CRD42017068915)

Keywords: individual participant data meta-analysis, risk prediction model, Zika virus, microcephaly, congenital Zika syndrome, prognosis, Bayesian methods, data sharing

Strengths and limitations of this study

- This is one of the first applications of an IPD-MA to address public health concerns in the context of an emerging pathogen. Lessons learned from this IPD-MA may facilitate the formation of research collaborations to inform the public health response to future epidemics.
- By using a diversity of populations to develop and validate the risk prediction tool that identifies pregnancies at the highest risk of CZS, the IPD-MA provides a real opportunity to help inform how clinicians and laboratory scientists communicate ZIKV results to pregnant women and their families.
- There is a high degree of variability in the accuracy of diagnostic assays for ZIKV, co-infection, and outcome ascertainment. Addressing this variability will be a challenge and ultimately a limitation of the accuracy of IPD-MA results.
- There is no gold standard diagnostic assay to detect ZIKV infection during pregnancy and few studies have been able to measure fetal infection. The statistical methods traditionally used to synthesize IPD across clinical studies and randomized controlled trials of need to be adapted to account for these myriad sources of uncertainty.

For peer review only - http://bmjopen.bmj.com/site/about/guidelines.xhtml

INTRODUCTION

Zika virus (ZIKV) infection during pregnancy is an acknowledged cause of microcephaly and other forms of fetal brain defects and disability.[1 2] ZIKV is an arbovirus in the genus Flavivirus that is usually transmitted through the female *Aedes aegypti* mosquito. *Aedes aegypti* is also the main vector for dengue (DENV), urban yellow fever (YF), and chikungunya viruses. The Asian strain of ZIKV has been shown to replicate in the placenta and fetal brain;[3] ZIKV transmitted from mother to fetus during pregnancy may have a detrimental effect on fetal brain development.[4-6] Microcephaly, generally defined as a 2-3 standard deviation reduction from the mean head circumference,[7 8] is caused by infections during pregnancy, maternal diet, drug abuse, genetic factors, or environmental exposures.[9 10] Microcephaly (congenital or acquired) may be associated with developmental delays; intellectual, hearing, and visual impairment; and epilepsy.[11] The causal relation between ZIKV and a spectrum of fetal anomalies that includes microcephaly, now known as congenital Zika syndrome (CZS),[12] has been supported through several case-control;[13 14] cohort;[15 16] and surveillance studies;[17] animal and cell studies;[18] and through two systematic reviews of the evidence for causality that considered all study designs.[1 2] The relation between ZIKV infection during pregnancy and miscarriage (pregnancy loss <20 weeks gestation) and fetal loss (pregnancy loss ≥20 weeks gestation) is still under investigation.

Prior to the 2013-16 epidemic waves, ZIKV infection was known clinically as a mild illness characterized by symptoms shared with other arboviruses, including: maculopapular rash; headache; fever; non-purulent conjunctivitis; and/or joint and muscle pain.[19] During the 2015-16 ZIKV outbreak in Brazil, which extended to a number of other Latin American countries, there was a sharp increase in reports of microcephaly and other neonatal neurological conditions and in Guillain-Barré syndrome (GBS),[20-22] an autoimmune neurologic disorder. Subsequent analysis of medical records collected during and after the 2013-2014 ZIKV outbreak in French Polynesia identified several ZIKV-linked pregnancies that had not been recorded earlier because they ended in elective abortion or stillbirth. The re-analysis of medical records indicated that the prevalence of both microcephaly and GBS had increased in the wake of the outbreak in French Polynesia.[23 24] The Pan American Health Organization (PAHO) issued a ZIKV Epidemiological Alert for Member States on May 7, 2015,[25] the Brazilian Ministry of Health (MOH) declared a national public health emergency due to the time and cluster of microcephaly cases identified in Northeastern Brazil on November 12, 2015,[26] and the World Health Organization (WHO) declared that the clusters of microcephaly and related neurological complications represented a Public Health Emergency of International Concern on February 1, 2016.[27]

Zika virus presents myriad challenges from an epidemiological, virological, diagnostic, and outbreak control perspective. Diagnosing ZIKV infection is complicated by the absence of symptoms in most cases or the presence of non-specific symptoms; cross-reactivity with DENV;[28 29] the short window for diagnosing acute infection; and the lack of point-of-care diagnostics.[30] Recent research suggests that the relation between ZIKV infection during pregnancy and fetopathology may vary by virus genotype or lineage; primary versus secondary infection;[31] and DENV-immune status and genotype in the presence of coinfection[29 32 33] The unequal spatial distribution of microcephaly cases has been discussed extensively.[34-36] These differences may be related to population-level differences in baseline risk of

For peer review only - http://bmjopen.bmj.com/site/about/guidelines.xhtml

adverse fetal outcomes (clinically important heterogeneity), differences in study design (e.g. inclusion criteria; measurement of important co-factors), or to measurement error, defined as the difference between the observed and actual level of a given variable. Laboratory confirmation of ZIKV infection and co-infection differs by diagnostic algorithms (e.g. definition of positive and negative ZIKV diagnostic assay results); factors that affect the regularity of testing (e.g. provision of incentives, distance from testing center, differences across protocols); population-specific distribution of related co-infections; differing levels of training of laboratory staff; and the accessibility of materials and technology (e.g., ultrasound, immunoassays, reliability panels), among other factors. In addition to documented difficulties in accurately measuring infant head circumference, measurement standards for identifying microcephaly differ across populations and standards themselves may not appropriately classify reduced or enlarged head circumference.[37 38]

Our limited understanding of the absolute risk of adverse fetal, infant, and child outcomes in ZIKV-infected mothers led to calls from several governments suggesting that women avoid becoming pregnant for as long as two years.[39 40] ZIKV disproportionately affects low-income populations residing in areas with poor living conditions.[41] The impetus placed on women to delay pregnancy as a ZIKV control measure is complicated by the limited access to contraception and safe abortion in many of the countries and regions with the highest burden of ZIKV-related microcephaly.[42 43] Identifying the risk factors for CZS is a global health priority and central for prioritizing resource allocation for vector control and effective and targeted family planning interventions, and for improving risk counseling for ZIKV-infected pregnant women or women planning a pregnancy in endemic areas.

Rationale for the individual participant data meta-analysis of longitudinal studies of pregnant women

Individual participant data meta-analysis (IPD-MA) is the quantitative synthesis of participant-level data from included studies, while appropriately accounting for the clustering of information at the study level. The proposed IPD-MA will combine de-identified, participant-level cohort data from different populations of pregnant women to identify and quantify the relative importance of different predictors of CZS. Individual participant data (IPD) have a number of analytic benefits over aggregate data meta-analysis (AD-MA), a form of knowledge synthesis that combines study-level measures of effect.[44 45] Individual participant data facilitates the assessment of effect measure modification, the development and validation of risk prediction models, and the application of a unified analytic approach. In addition to using the same statistical model across studies, with IPD we can apply the same or similar exclusion criteria, diagnostic algorithms, methods for addressing missing data and confounding, and conduct the same types of sensitivity analyses needed to explore unexplained within- and between-study heterogeneity.

Increased precision of estimates

Timely, accurate, and reliable predictions are predicated on well-designed studies that minimize the risk of bias, adequate sample size, and the inclusion of a diversity of populations. Adequate sample size is

For peer review only - http://bmjopen.bmj.com/site/about/guidelines.xhtml

crucial for precise estimation of the risk of CZS within important subgroups (e.g. women infected during the first trimester; pregnant women with previous or concurrent DENV, CHIKV, and STORCH pathogen exposure). Vector control measures, including pesticides, public education campaigns, the use of drones to detect standing water, and the introduction of sterilized male vectors to reduce *Aedes aegypti* populations, have been implemented in the wake of the 2015/2016 ZIKV epidemics.[46-48] Fortunately, these measures, in combination with other factors that are currently being investigated, seem to have reduced the numbers of ZIKV infections during the 2017/2018 epidemic cycle. While many studies have followed infants to the end of their first year, certain developmental milestones can only be assessed after age two[49] or when a child reaches school age. Leveraging limited data from studies with extended follow-up of ZIKV-infected and non-infected women will be essential for estimating the risk of more subtle, long-term effects of ZIKV infection during pregnancy. By combining data from individual studies, the proposed IPD-MA will improve the precision of risk estimates.

Identify and quantify the relative importance of effect measure modifiers

The benefits of using IPD rather than AD to assess effect measure modification and interaction are myriad.[50] IPD can be analyzed in either a one- or a two-stage meta-analysis while AD can only be meta-analyzed using a two-stage approach. In a one-stage analysis with IPD, subject level data are meta-analyzed using the exact binomial distribution; in a two-stage analysis of IPD or AD, study-level outcome measures are combined assuming asymptomatic normality.[51] In a one-stage analysis of IPD, study- and individual-level sources of heterogeneity can be assessed concurrently and IPD are better able to identify heterogeneity in the context of rare events or small studies.[50 52] Individual studies are often powered to detect the overall effect of the exposure rather than subgroup effects. Due to variations in the characteristics of the affected populations and in the potential confounders and effect modifiers measured by different studies, it is unlikely that individual studies will be powered to definitively quantify the importance of different sources of heterogeneity in the relation between ZIKV infection during pregnancy and adverse fetal, infant, or child outcomes.

Clinical risk prediction to inform decision-making and resource allocation

While there are a number of vaccine trials underway,[53] the development of a ZIKV vaccine is complicated by the necessity of testing the vaccine in pregnant women; assessing whether the vaccine is associated with development of GBS; the difficulties inherent in developing an arbovirus vaccine;[46 54-56] findings from *in vivo* studies that indicate cross-reactivity between ZIKV and DENV or West Nile virus is related to antibody-dependent enhancement of ZIKV infection;[55 57 58] and by the potential use of prevention of infection as a vaccine efficacy endpoint.[59] In this context, identifying the pregnancies at the highest risk of adverse neonatal and later developmental outcomes is critical for effective resource allocation and prevention strategies. We will use participant-level data to develop and externally validate clinical risk prediction models to facilitate the identification of pregnancies that are most likely to result in ZIKV-related adverse fetal or infant outcomes and longer-term developmental delays.

Standardization and cross-national partnerships to inform the public health response to emerging pathogens

For peer review only - http://bmjopen.bmj.com/site/about/guidelines.xhtml

Formation of the ZIKV IPD Consortium

The ZIKV IPD Consortium is a global collaboration designed to streamline the international response to ZIKV. To facilitate cross-country analyses and a coordinated response to ZIKV, representatives from WHO, PAHO, the US Centers for Disease Control and Prevention (CDC), the National Institutes of Health (NIH), the National Institute of Allergy and Infectious Diseases (NIAID), Institut national de la santé et de la recherche médicale (INSERM), Institut Pasteur, and the networks of Fundação Oswaldo Cruz (Fiocruz), Grupo de Pesquisa da Epidemia da Microcefalia (MERG)/ZikaPlan, ZIKAlliance, ZIKAction, the Consortium for the Standardization of Influenza Seroepidemiology (CONSISE), and International Severe Acute Respiratory and Emerging Infection Consortium (ISARIC) have developed a standardized protocol for cohorts of pregnant women and their infants exposed to ZIKV to facilitate the proposed IPD-MA; identified existing or planned cohorts; and prospectively introduced cohort principal investigators (PI)s and MOH officials to the methodological and public health benefits related to IPD-MA in the context of Zika. Many of the longitudinal studies and surveillance systems identified to date through the review of country-level registries, existing literature reviews, and ZIKV IPD Consortium membership have agreed to contribute de-identified, participant level data to the analysis. A complete list of the studies and surveillance systems who have agreed to contribute data to the ZIKV IPD Consortium led IPD-MA is included in **Supplementary Table 1**.

Standardized protocols for cohorts of pregnant women and their infants

A multiplicity of mechanisms for exposure and outcome ascertainment as well as differences in the measurement of important cofactors are known challenges for the meta-analysis of data from individual research studies. To minimize the potential for heterogeneity caused by differences in study inclusion criteria and the measurement of ZIKV, infant outcomes, and important cofactors, WHO/PAHO, Institut Pasteur, Fiocruz, CONSISE, and ISARIC convened an international meeting of ZIKV researchers and MOH officials in June of 2016 to develop standardized protocols and data collection instruments for cohort studies of pregnant women and newborns and other ZIKV-related studies.[60] Standardization of protocols and data collection instruments was intended to minimize differences in case ascertainment and data collection methods to facilitate data synthesis and the identification of sources of heterogeneity in the relation between congenital Zika infection and adverse fetal, infant, and child outcomes. The protocols were made available on WHO website in October 2016 (http://www.who.int/reproductivehealth/zika/en). The standardized protocols do not include detailed guidance on laboratory methods, but testing algorithms were developed by an expert panel and made available on the WHO website in March 2016 (http://www.who.int/csr/resources/publications/zika/laboratory-testing/en/). The IPD-MA will need to account for the between- and within-study differences in diagnostic assays and testing algorithms.

For peer review only - http://bmjopen.bmj.com/site/about/guidelines.xhtml

OBJECTIVES OF THE IPD-MA

1. Estimate the absolute and relative risks of fetal infection; miscarriage (<20 weeks gestation), fetal loss (≥ 20 weeks gestation), microcephaly, and other manifestations of CZS and later developmental delays for women who do and do not experience ZIKV infection during pregnancy.

2. Identify factors that modify women's risk of adverse ZIKV-related fetal, infant, and child outcomes and infants' risk of infection (e.g. gestational age at time of infection, clinical or subclinical illness, concurrent or prior arbovirus exposure, other congenital infections, and other posited effect measure modifiers).

3. Use information on the relative importance of different effect measure modifiers identified in Objective 2 to decompose the total effect of ZIKV infection during pregnancy on adverse fetal, infant, and child outcomes into 1) the direct effect of ZIKV; 2) the indirect effect of ZIKV as mediated by the effect measure modifier of interest (e.g. DENV, CHIKV, or STORCH pathogens); and 3) the effect of the interaction between ZIKV and the mediator of interest.

4. Develop and validate a risk prediction tool to identify pregnant women at a high risk of an adverse ZIKV-related outcome and to inform couples planning a pregnancy, healthcare providers, and/or resource mobilization (e.g. vector control strategies; antenatal care; open access to contraception).

METHODS & ANALYSIS

This protocol has been drafted in accordance with the PRISMA-P Statement (**Supplementary Table 2**).[61] The proposed systematic review and meta-analysis will follow the PRISMA-IPD guidelines for the systematic review of non-randomized studies.[62]

Step 1. Study identification

Eligibility criteria

Eligible studies will use a longitudinal design where ZIKV infection is measured in pregnant women prior to outcome ascertainment. Eligible studies may include cohort studies, case-cohort studies, randomized control trials, or active surveillance systems. Studies may enroll symptomatic and/or asymptomatic women prior to or following a confirmed pregnancy. Included studies will test women for ZIKV infection during pregnancy, follow women until the end of pregnancy, and assess for CZS or related fetal, infant, or child outcomes (see Table 1). We will exclude studies with fewer than 10 participants and limit included surveillance systems to those that capture country or territory-level active surveillance data (i.e. individual hospital active surveillance data will not be included). Before

For peer review only - http://bmjopen.bmj.com/site/about/guidelines.xhtml

sharing participant-level data, research studies will be asked to provide documentation of ethics review.

Information sources

1. ZIKV IPD Consortium

We anticipate that most eligible studies will have been identified through the efforts of the ZIKV IPD Consortium. The Consortium is an international initiative that is meant to include the PIs from all planned, ongoing, or completed ZIKV longitudinal studies at the time of this review. We have searched clinical trials and ZIKV-related databases[63] (**Supplementary Table 3**) to identify existing or planned longitudinal studies. We have circulated the list of ongoing or planned ZIKV-related longitudinal studies of pregnant women to MOH Officials in countries with autochthone ZIKV transmission and to PIs of ZIKV cohorts and asked them to update the list as necessary.

2. Systematic review

We will perform a systematic search of biomedical databases for published longitudinal studies and protocols. The search strategy is based on Medical Subject Headings (MeSH) and text-based search terms for ZIKV, pregnant women, infants, and children. The search strategy was developed in collaboration with an information scientist and adapted for the following electronic databases: Embase(Medline), Embase(Ovid), and SCOPUS (see **Supplementary Text 1** for the search strategy for Embase (Medline and Ovid). We also will search the additional databases listed in **Supplementary Table 3** and review the reference lists of published systematic reviews and the list of studies produced by a living systematic review of ZIKV studies conducted by the University of Bern[64] to identify additional studies. After removing duplicates from the list of identified studies, two reviewers will independently screen the title and abstracts of included studies to identify longitudinal studies or active surveillance systems that measure ZIKV infection during pregnancy and subsequent fetal, infant, or child outcomes. Disagreements about study inclusion will be resolved by consensus.

Collection of study-level data

We will contact the PIs of eligible studies identified through either the ZIKV IPD Consortium or the electronic searches to invite them to take part in the IPD-MA and ask them to provide a copy of their study protocol. We will develop and pilot an electronic data extraction form to record study-level characteristics for all eligible studies, regardless of whether study PIs agree to participate in the IPD-MA. Two reviewers will independently review protocols and study-related publications to extract data on study design; study population; enrollment, follow-up and laboratory procedures; assay and specimen type; criteria used to define ZIKV infection and timing of infection; and exposure, cofactor and outcome ascertainment for all eligible studies. We will ask study PIs for clarification if there are outstanding questions or disagreements regarding study-level data.

Step 2. Collection, review, and synthesis of de-identified, participant-level data

For peer review only - http://bmjopen.bmj.com/site/about/guidelines.xhtml

We will contact the PIs and authors of studies that meet our inclusion criteria to request de-identified, participant-level data on select variables and the associated surveys and data dictionaries or codebooks. If study data have been imputed, we will request both the original and imputed data so that we can apply consistent imputation methods across studies and review the imputed dataset for validation purposes. To reduce the burden on individual studies and ensure clear documentation of all steps in the creation of the synthesized dataset, we will use the study codebooks or data dictionaries to develop study-specific code in the statistical language used by the study data manager that selects only the study variables required for the proposed analyses and removes information that could be used to identify individual participants. The study's data manager will apply the code to the original dataset. The de-identified, participant-level data will be transferred from the study site to Emory University, which will serve as the WHO data synthesis partner center, using secure file transfer protocol and will be protected on a secure server with standard encryption and by the Emory University firewall. Data synthesis-related decisions will be reviewed by a ZIKV IPD Consortium membership and will be recorded using Jupyter Notebook.[65] Researchers that are unable or unwilling to provide their participant data after at least four attempts at contact by the project team over a period of six months will be excluded from the IPD-MA and we will report the reason for their exclusion. When IPD are not available for a given study, we will extract study-level effect estimates from any publications to compare study-level estimates from all eligible studies, whether or not they provide data for the IPD-MA.

Variables of interest

Despite efforts to develop protocols that can be applied across studies, there will be significant cross-study heterogeneity in how congenital Zika infection, cofactors, and outcomes are measured and reported. Exposure, outcome variables, and posited confounders and effect measure modifiers are listed in Table 1. Given that the case definitions for microcephaly have changed over time (and may change during the course of included studies), we will allow for the coding of variables with different definitions (i.e. WHO fetal growth chart,[66] Fenton scale[67], INTERGROWTH 21st Project[49]). Definitions for miscarriage, fetal loss, and other pregnancy outcomes vary across countries. We will explore the sensitivity of project findings to different outcome definitions.

Table 1. Participant-level variables of interest

Exposure	Maternal ZIKV infection (binary; categorical: confirmed, probable, unlikely; primary, secondary, naïve; continuous: viral load)
	Fetal or placental ZIKV infection (binary; categorical: confirmed, probable, unlikely; primary, secondary, naïve; continuous: viral load)*
Primary outcomes	Miscarriage (binary: <20 weeks gestation)
	Fetal loss (binary: ≥20 weeks gestation)
	Microcephaly (binary; categorical: severe microcephaly, microcephaly, normocephaly, macrocephaly; continuous: Z-score)
	CZS (binary; categorical: confirmed, probable, unlikely)
Secondary fetal outcomes†	Induced abortion with microcephaly (categorical: confirmed, probable, unlikely)
	Early fetal death (binary: 20-27 weeks gestation)

For peer review only - http://bmjopen.bmj.com/site/about/guidelines.xhtml

	Late fetal death (binary: ≥28 weeks gestation)
	Late fetal death (≥28 weeks gestation) with microcephaly (binary)
	Placental insufficiency (binary; categorical: confirmed, probable, unlikely)‡
	Intrauterine growth restriction (binary)
Secondary infant outcomes†	Postnatal microcephaly (binary; categorical: severe microcephaly, microcephaly, normocephaly, macrocephaly; continuous: Z-score)
	Gestational age at birth (continuous)
	Birth weight (categorical: normal birth weight; low birth weight; very low birth weight; extremely low birth weight; continuous: Z-score)
	Craniofacial disproportion (binary)
	Neuroimaging abnormalities (binary: intracranial calcification, lissencephaly, hydranencephaly, porencephaly, ventriculomegaly, posterior fossa abnormalities, cerebellar hypoplasia, corpus callosal and vermian dysgenesis; focal cortical dysplasia)
	Postnatal intraventricular hemorrhage (binary)
	Motor abnormalities (binary: hypotonia, hypertonia, hyperreflexia, spasticity, clonus, extrapyramidal symptoms)§
	Seizures, epilepsy (binary)§
	Ocular abnormalities (binary: blindness, other)§
	Congenital deafness or hearing loss (binary)§
	Congenital contractures (binary: arthrogryposis, uni or bilateral clubfoot)
	Other non-neurologic congenital abnormalities (binary)
Secondary outcomes detected after the infant period**	Cortical auditory processing
	Neurodevelopment (expressive and receptive language, fine and gross motor skills, attention and executive function, memory and learning, socioemotional development, overall neurodevelopmental score)
	Vision (Cardiff test)
Posited confounders	Demographic factors (age, education, marital status, racial/ethnic group; BMI)
	Socioeconomic factors
	Maternal smoking, illicit drug and alcohol use
	Maternal prescription drug use, vaccination
	Maternal experience of violence during pregnancy; infant or child exposure to intimate partner violence [68]
	Workplace or environmental exposures to teratogenic substances (e.g. maternal exposure to lead, mercury)
Potential effect measure	Genetic anomalies, metabolic disorders, perinatal brain injury

For peer review only - http://bmjopen.bmj.com/site/about/guidelines.xhtml

modifiers	
	Gestational age, term at birth
	Timing of infection during pregnancy
	Clinical/subclinical illness
	Viral genotype and load
	Concurrent or prior flavi- or alphavirus infection
	Maternal history of YF or JE vaccination
	Maternal immunosuppressive conditions, disorders, comorbidities (e.g. chronic hypertension, diabetes), or pregnancy-related conditions (e.g. pre-eclampsia, gestational diabetes)
	Intrauterine exposure to STORCH pathogens
	Maternal malnutrition
	Presence and severity of maternal and infant clinical symptoms

CZS=congenital Zika syndrome, JE=Japanese encephalitis; STORCH=syphilis, toxoplasmosis, rubella, cytomegalovirus, and herpes; YF=yellow fever virus; ZIKV=Zika virus
*Fetal ZIKV infection will be considered as both an exposure and an outcome; definition of fetal infection will be based on clinical and radiological criteria defined by an expert panel
†Both with and without microcephaly
‡As estimated by antenatal consequences of placental insufficiency, including fetal growth restriction, oligohydramnios, non-reassuring fetal heart rate tracing or small for gestational age at birth as markers of placental insufficiency.
§May also be detected after the infant period
** As measured by the Bayley Scale;[69] Ages and Stages;[70] INTERGROWTH-21st Neurodevelopmental Assessment[49]

Assessing the integrity of de-identified, participant-level data

We will review the distribution of variables to identify potential outliers and to assess the proportion missing within each study. We will discuss the distribution of key variables with the study data manager to identify and address any inconsistencies. If there has been a publication related to a given longitudinal study, we will attempt to replicate the Table 1 presented in the publication and will resolve any inconsistencies with the data manger.

Synthesis of participant-level data

Given that these longitudinal studies and active surveillance systems are part of the global research response to an emerging pathogen, there is a high degree of variability in the data that have been collected across studies and the algorithms that have been applied to define ZIKV exposure, symptoms, components of CZS, etc. Where possible, we will ask studies for the individual factors (i.e. fever, rash) that were used to define certain parameters (i.e. clinical infection) to ensure cross-study consistency in composite markers. Similarly, we will combine the data inputs for exposure, cofactor, and outcome classification algorithms to reduce cross-study differences in the classification of important factors.

Critical review of study quality

We will use the Cochrane Methodological Quality Assessment of Observational Studies[71] and the Q-Coh tool[72] to help describe the risk of bias within non-randomized studies and will apply the Cochrane Risk of

For peer review only - http://bmjopen.bmj.com/site/about/guidelines.xhtml

Bias 2.0 tool to assess the risk of bias in randomized controlled trails.[73] Rather than using a score-based bias assessment, a panel that includes experts on the evaluation of laboratory assays and external quality assessment (EQA); obstetrics; and perinatal epidemiology will provide a detailed description of the role of selection, confounding, and measurement bias within studies.

Step 3. Statistical analyses

***Objectives 1 & 2**. Estimate the absolute and relative risks of adverse ZIKV-related fetal, infant, and child outcomes; identify and quantify relative importance of sources of heterogeneity*

Estimating the absolute risk of CZS by the gestational age of the fetus at the time of infection is as important as it is difficult. Early in the outbreak, cohort studies limited enrollment to symptomatic pregnant women. While an estimated 50-70% of infections are subclinical, when symptoms are detected they generally appear 3-14 days after infection.[74] For asymptomatic infections, the gestational age of infection is interval censored because it is defined by the last negative and first positive tests for ZIKV. Rather than using the midpoint between the last negative and first positive ZIKV test, which is known to be biased, we will impute the trimester or week that asymptomatic infections occurred using methods that are routinely applied in studies with interval censored covariates in the field of perinatal research.[75] [76] In Table 2, we present sample definitions for the absolute risk of fetal and infant outcomes. These definitions will be reviewed prior to analysis and publication and we will assess the sensitivity of our results to the definition applied.

Table 2. Definitions applied to estimation of absolute risk of primary fetal and infant outcomes

Outcome	Numerator	Denominator
Miscarriage	number of miscarriages (pregnancy loss prior to 20 weeks gestation)	total number of pregnancies
Early fetal death	number of pregnancies lost between 20-27 weeks gestation	total number of pregnancies carried to 20 weeks gestation
Late fetal death	number or pregnancies lost at or following 28 weeks gestation	total number of pregnancies carried to 28 weeks gestation
Microcephaly	number of microcephaly cases	total number of pregnancies carried to ≥24 weeks gestation, when microcephaly can be assessed by ultrasound in ZIKV-infected mothers,[38] we will consider all pregnancies regardless of whether the pregnancy results in a live birth.

We will apply mixed binomial models for binary outcomes, and multinomial models for categorical outcomes, with a logit link to provide estimates for each measure of absolute risk by week or trimester

For peer review only - http://bmjopen.bmj.com/site/about/guidelines.xhtml

of congenital infection. Because of the differences in baseline risks across populations, pooling measures of absolute risk across studies may not be clinically relevant and can even be misleading.[77] We will combine study-level estimates of absolute risk through: 1) a one-stage meta-analysis (mixed binomial or multinomial model with a log link) that includes study-level sources of heterogeneity and a separate intercept for each study to account for additional cross-study differences in baseline risk; and 2) a forest plot of study-level estimates of absolute risk that does not include a summary meta-analytic estimate.

Absolute measures of effect are considered more important for informing clinical practice than relative measures.[78] We will conduct both 1) a one-stage meta-analysis where we estimate the relative risk of the aforementioned outcomes of interest by congenital Zika infection across studies and 2) a two-stage meta-analysis where we estimate the relative risk in each study and combine study-level measures using random effects meta-analysis to allow the underlying true effect to vary across studies.[79] In the one-stage models, we will include study-specific intercepts to quantify and account for between-study variation in baseline risk. We will use random slopes to allow the relation between certain cofactors and the risk of CZS to vary across populations.

Combining absolute measures of effect, like the risk difference, across studies may mask important differences in the baseline risk. We will present estimates of the risk difference in a forest plot of study-level estimates without presenting a summary meta-analytic estimate. In both the one- and two-stage analyses, we will use log binomial regression models to estimate the relative risk of each binary outcome and will use log Poisson regression to estimate the relative risk if log binomial models fail to converge.[80] [81] In the two-stage models, we will assess the potential for non-linear relationships between continuous exposures (viral load) and covariates (e.g. gestational age, maternal age) by using the Akaike information critiera to compare restricted cubic splines with 3 knots to exponential, quadratic, and linear terms. In the one-stage models, we will use generalized additive mixed models (GAMMs) to assess potential non-linearities as the GAMM random smoothing parameter addresses the bias/variance trade-off by penalizing the added complexity from non-linear terms while accounting for between-study variation in non-linear effects.[82]

Joint estimation of multiple nested or otherwise related outcomes (multivariate meta-analysis)

Not all studies will have measured all primary or secondary outcomes of interest. For example, most studies will have measured ventriculomegaly, but may not include values for intracranial calcification or ocular abnormalities.[9] This analysis is intended to increase the precision of estimates of the spectrum of CZS abnormalities. Studies that do not include the measurement of a given outcome will necessarily be excluded from univariate estimates of that outcome, but will be included in multivariate models that estimate the joint probability of related outcomes. In the multivariate models, we will assume that the outcomes that are excluded from certain studies are missing at random and will incorporate studies by setting the missing observations and within-study correlations between outcomes to zero and will set the within-study variance to a very high number such that the artificial value that acts as a substitute for the missing outcome will have a negligible effect on the meta-analytic estimate from the multivariate model.[83] Alternatively, under a Bayesian framework, we will model a joint distribution for studies

For peer review only - http://bmjopen.bmj.com/site/about/guidelines.xhtml

providing multiple outcomes and a univariate distribution for studies providing a single outcome without needing to address the missing within-study correlations and variance for studies with only one outcome.[84] The secondary outcomes that will be included in the multivariate analysis are listed in Table 1.

We will compare generalized linear mixed models (GLMMs) where we use one model to analyze nested or otherwise related outcomes to the standard univariate approach where we apply a separate model to analyze each outcome. Multivariate meta-analysis allows for the estimation of joint probabilities across multiple outcomes and accounts for cross- and within-study correlation between related outcomes.[83] Modelling several outcomes simultaneously improves the precision over univariate models by sharing information about heterogeneity and the average effect of the treatment which may facilitate inference about the relation between different CZS-related outcomes[83] (i.e. vermian dysgenesis and ocular abnormalities).

Multivariate model to combine estimates from fully and partially adjusted studies

A number of longitudinal studies will not include the minimal sufficient set of confounders. Estimates from partially adjusted studies (that are missing values for important confounders) will be combined with fully adjusted estimates in a one-stage multivariate meta-analysis. The one-stage multivariate model allows us to borrow information from partially adjusted studies with different sets of confounders while ensuring that we control for important confounders.[83]

Special considerations for the meta-analysis of cohort studies with rare events

Two-stage meta-analytic methods are based on large sample approximations, and may be unsuitable in the context of CZS, which can be considered a rare event.[85 86] Two-stage meta-analysis may be biased when small studies are included, the effect of an exposure is very large, or the outcome is rare, all of which may affect this analysis.[87] We will highlight any instances when the two-stage meta-analytic estimates may be biased by the aforementioned issues and will limit our inference to one-stage analyses in those cases. If we have a number of longitudinal studies with zero events, we will focus our inference on a one-stage approach to avoid reliance on large sample approximations.

Assessment of study- and participant-level heterogeneity

Separating within- and between- study heterogeneity is central to assessing participant-level heterogeneity and to understanding the relative importance of different potential effect measure modifiers.[50] We are only able to separate within- and between-study heterogeneity across studies that include both levels of the effect measure modifier of interest. The presence of clinical illness may be related to disease course through viral load or be a marker for the strength of the immune system's response to infection. We will conduct a one-stage analysis of longitudinal studies that include both symptomatic and asymptomatic women to assess whether the risk of CZS or of the most severe effects of congenital infection (miscarriage, fetal loss) differs for clinical and subclinical infections. Between-

For peer review only - http://bmjopen.bmj.com/site/about/guidelines.xhtml

study heterogeneity is reflective of study-level differences, while within-study heterogeneity may be indicative of clinically important differences. We will mean center covariates included in the interaction terms at the study level to separate between- and within-study heterogeneity in our one-stage meta-analytic estimates of how prior or co-infection with alpha or flaviviruses or STORCH pathogens modifies the effect of ZIKV infection.[88]

Heterogeneity in effect estimates will arise from clinically important differences between congenital infections or women (effect measure modification) and from study-level differences in exposure and outcome ascertainment (measurement bias). With IPD, we are able to jointly assess study- and participant-level heterogeneity.[52] We will incorporate participant-level interaction terms in a one-stage analysis that includes random intercepts to account for unmeasured study-level factors. We will consider random slopes for certain covariates to allow for between-study variation in covariate effects across studies. Given the difficulty in assessing the total degrees of freedom in mixed models, we will apply bootstrapping to assess the approximate confidence intervals of the pooled interaction terms. We will present the analysis of effect measure modifiers in accordance with the revised STROBE guidelines.[89]

Based on our review of research protocols for planned or ongoing cohort studies, we expect to include data from longitudinal studies with different enrollment criteria, exposure and outcome ascertainment, diagnostic assays for prior- or co-infections, and measurement of important cofactors. We will include measures of study-level sources of heterogeneity (e.g. diagnostic assay, outcome definitions) as covariates in the one-stage regression to assess the variance explained by these factors. We will perform a sensitivity analysis where we limit our inference to studies with similar inclusion criteria and exposure, cofactor, and outcome ascertainment to reduce spurious cross-study heterogeneity. While two-stage analyses of interaction effects are subject to ecological bias and our inference about the importance of interaction terms will primarily be derived from one-stage analyses, we will use a two-stage analysis to compare the magnitude of the interaction effects across studies. The interaction between certain cofactors and ZIKV exposure may not be consistent across studies. In the first stage of the two-stage analysis, we will use the likelihood ratio test (P-value < 0.05) to assess the importance of including interaction terms within each study. Individual cohort studies may not have the sample size needed to detect clinically important interactions between ZIKV and important cofactors. We will also assess whether a certain interaction is consistent across studies, while not necessarily statistically significant within individual studies.

Meta-regression and subgroup analyses have limited power to detect interactions and can only be used to make inference about the relation between the exposure and study-level, average values of participant characteristics.[87 90] Studies that are not willing or able to provide participant-level data may differ importantly from longitudinal studies whose data is included in the IPD-MA. We will apply subgroup analysis to a two-stage analysis of effect estimates from studies included in the IPD-MA and published estimates from studies that did not participate in the IPD-MA to assess whether study-level variation in recruitment and enrollment criteria, exposure and outcome ascertainment, and measurement of co-infections and other cofactors are important sources of heterogeneity in the pooled

For peer review only - http://bmjopen.bmj.com/site/about/guidelines.xhtml

estimates. Some sources of heterogeneity (e.g. vector density and feeding patterns; DENV serotype) may not be measured and should be considered in sensitivity analyses.

Objective 3. *Use information on the relative importance of different effect measure modifiers identified in Objective 2 to decompose the total effect of ZIKV infection during pregnancy on adverse fetal, infant, and child outcomes.*

Some studies suggest that antibody-dependent enhancement related to concurrent or prior DENV infection or Japanese encephalitis vaccination may modify the effect of ZIKV infection on fetal development. Both the timing of exposure to DENV and DENV serotype may contribute to regional differences in the strength of the relation between ZIKV infection and CZS.[28 32] We will apply inverse probability of treatment weighted-marginal structural models to decompose the total effect of concurrent or prior DENV infection into the direct effects of ZIKV infection, the effect of ZIKV infection mediated by DENV, and the effect of the interaction between ZIKV and DENV.[91 92] If warranted, we will conduct a causal mediation analysis with additional effect measure modifiers identified through Objective 2-related analyses.

Objective 4. *Develop and validate a risk prediction tool to inform decision making by pregnant women, couples planning a pregnancy, and healthcare providers, and/or resource mobilization*

We will fit one-stage logistic regression models with random intercepts to account for differences in the baseline risk within each study. We will apply group Lasso regression[93] to identify the prognostic variables that predict progression to miscarriage, fetal loss, and microcephaly. Lasso regression is implemented using L1-penalized estimation. The application of group Lasso ensures that the algorithm selects all levels of categorical variables by treating corresponding dummy variables as a group instead of allowing the model to only select certain levels of categorical variables.[94 95] The L-1 penalty term allows for concurrent consideration of predictors and shrinkage, which facilitates variable selection in the context of high dimensional data.[96] We will standardize included variables so that all variables use the same scale. We will adopt cross-validation on the study level to select the optimal tuning parameter (λ) and will adopt restricted maximum likelihood (REML) to estimate the variance-covariance matrix of the study-level random effects.

Not all studies will have the resources to implement the most accurate and reliable ZIKV-related diagnostic tools. As part of the data synthesis, we will identify the exposure and cofactor diagnostic methods that are most commonly applied. As a sensitivity analysis, we will use these diagnostic methods to develop a risk prediction model so that the model can be applied in regular clinical practice.

Development and external validation of the prediction model

We will apply internal-external cross-validation wherein we rotate the cohort that is used for external validation to improve the model's predictive ability.[97] For example, given k cohort studies, we will

For peer review only - http://bmjopen.bmj.com/site/about/guidelines.xhtml

use $k-1$ cohort studies to develop the prediction model and will validate model performance by applying the prediction model to a cohort that was not used to develop the prediction model. Internal-external cross-validation allows for the use of all available data for model development and validation which improves model performance and generalizability.[98]

Evaluation of model performance

We will generate receiver operating characteristic (ROC) curves[99 100] in the cohort that was not used to develop the prediction model to estimate the model's true-positive (sensitivity) versus false-positive (1-specificity) rate for each binary outcome. These curves will then be summarized using the area under the ROC curve (AUC). In some instances, the pregnant woman or couple planning a pregnancy may prefer a more sensitive rather than a more specific model. We will present a range of cut-off values that maximize sensitivity, specificity, or both sensitivity and specificity to facilitate decision making by pregnant women or couples planning a pregnancy. We will assess the extent to which these thresholds yield consistent sensitivity and specificity across different regions and populations. We will use calibration plots to compare the observed and predicted probability of the outcome of interest within risk quintiles, and summarize these plots by calculating the total ratio of observed versus expected events (O:E ratio) and the calibration slope. Internal-external cross-validation of k studies will result in k AUCs, O:E ratios, and calibration slopes. We will apply random effects meta-analysis to combine estimates of the discrimination and calibration of the k predictive models. We will assess model calibration and discrimination and choose the model with the best properties.[97 101] We will use bootstrap validation to evaluate model optimism and will follow the TRIPOD statement guidelines for reporting the final prediction models.[102]

Step 4. Quantitative bias analysis

Given the complexity and level of measurement error, we will conduct a quantitative bias analysis under a Bayesian framework where we use a combination of expert opinion, laboratory EQA, and external and internal assessment of the relative accuracy of diagnostic assays and other methods for cofactor and outcome ascertainment to inform the prior distributions of bias parameters. We will use the GRADE criteria[103] to compare the quality of the evidence from Bayesian and frequentist models, with a focus on how imprecision, inconsistency, indirectness, magnitude of effect differ in the Bayesian and frequentist approaches.

Selection bias

Studies or surveillance systems that only recruit or test symptomatic pregnant women or studies that only enrolled pregnant women who tested positive for ZIKV infection are affected by selection bias because selection into the study is associated with the exposure.[63] This situation is similar to the inclusion of a single treatment arm in a randomized controlled trial. Although data from studies that only enroll pregnant women who test positive for ZIKV cannot directly inform estimates of the causal effect of ZIKV, these data can inform the development of prediction models because they contain

For peer review only - http://bmjopen.bmj.com/site/about/guidelines.xhtml

information on the prognosis of ZIKV positive women. Longitudinal studies that restrict enrollment to ZIKV positive pregnant women may also increase the precision of relative treatment effects by providing more events within ZIKV-exposed pregnant women. Longitudinal studies have reported that women who perceive their infants as unaffected by CZS are less likely to participate in follow-up. We will consider matching on the propensity score or the use of inverse probability of censoring weights[104] and prognostic score analysis[105] to account for measured determinants of differential loss to follow-up in the etiologic and prognostic models, respectively. Selection bias can be induced when we inappropriately adjust for a time-varying confounder affected by prior exposure (a confounder that also acts to mediate the relation between Zika virus infection and adverse fetal, infant, or child outcomes). We will use G-computation methods to appropriately adjust for time-dependent confounders affected by prior exposure.[106]

Confounding bias

We will adjust for confounders that are unlikely to mediate the causal relation between infection during pregnancy and adverse infant outcomes (Table 1). We will estimate each participant's likelihood of being infected during pregnancy, conditional on the study group and important confounders, to identify possible violations of the positivity assumption. In sensitivity analyses, we will apply propensity score matching within studies to ensure that important confounders are adequately balanced across exposure groups. Despite the prospective, collaborative development of a standardized research protocol for ZIKV cohort studies of pregnant women, confounders and effect measure modifiers may be defined differently across studies or not measured in certain studies. We will develop a detailed codebook that reflects the heterogeneity in confounder definitions and report on this heterogeneity in our analyses.

Measurement (i.e. detection, misclassification) bias

Despite efforts to harmonize case definitions across studies with the prospective development of a standardized protocol for cohorts of pregnant women and their infants,[60] the case definitions, diagnostic tools, and algorithms used to ascertain ZIKV infection, cofactors, and CZS-associated outcomes vary across studies.[107] The literature on the accuracy of ZIKV- and DENV-related assays is evolving rapidly.[30 108] Prior to initiating our analyses, we will synthesize the current evidence on the sensitivity and specificity of different assays for ZIKV diagnosis, for the assessment of concurrent or prior DENV infections, and for estimating the time of infection, amongst other relevant factors. The WHO standardized protocol for ZIKV-related cohorts of pregnant women includes WHO recommendations on the screening and assessment of neonates and infants with intrauterine ZIKV exposure;[109] we will compare study-level outcome definitions with the standardized WHO definitions. The role of heterogeneity related to case definitions and diagnostic tools will be explored through both frequentist and Bayesian methods. In the frequentist approach, we will: 1) include categorical or continuous markers of sensitivity and specificity of diagnostic tools as study-level covariates in the one-stage analyses and 2) apply diagnostic tool specific-subgroup analysis to both the one- and two-stage meta-analysis of effect measures from different studies. In the Bayesian approach, we will use a combination of expert opinion and data from external and internal validation studies to inform the probability distributions of bias parameters.[110]

For peer review only - http://bmjopen.bmj.com/site/about/guidelines.xhtml

Missing data

Missing data at the study level, as when confounders are not measured in certain studies, is a well-known challenge of IPD-MA[111 112] and a likely source of residual confounding. In keeping with current recommendations for addressing missingness in IPD-MA, we will apply new methods for multilevel multiple imputation to account for missing values.[113] As a sensitivity analysis, we will impute missing participant-level data in each study separately and use multivariate meta-analysis to combine data across studies that have and have not measured important host- and environmental-level cofactors.

Publication bias

IPD-MA may have a lower risk of publication bias than AD-MA because they include data from unpublished studies.[111] We have tried to ensure that the ZIKV IPD Consortium includes representatives from all of the academic and government institutions responsible for planned or ongoing ZIKV-related longitudinal studies of pregnant women and their infants. We expect that Consortium members will identify most ZIKV longitudinal studies and active surveillance systems of pregnant women and their infants, regardless of publication status, and we will conduct a systematic review to identify additional longitudinal studies and active surveillance systems. The degree of publication bias will be assessed visually by reviewing the asymmetry of study-level estimates from published and unpublished studies using funnel plots that compare log RR to the corresponding studies' sample size.[114]

We will convene a group of patient advocates to evaluate the ethical implications and utility of the risk stratification tool.

DISCUSSION

The application of IPD-MA to an emerging pathogen presents an important opportunity to harness global collaboration to inform the development of recommendations for pregnant women, couples planning a pregnancy, and public health practitioners. While IPD-MA offers real benefits compared to AD-MA or to the inference possible with individual cohort studies, the ability of IPD-MA to inform public health practice is directly related to the quality of the exposure, cofactor, and outcome ascertainment in the original cohort studies. Statistical methods for IPD-MA were developed in the context of clinical research and randomized control trials. These methods needs to be adapted to account for the myriad sources of uncertainty and bias that affect observational research, especially for field epidemiology studies conducted as part of the research response to unknown or emerging pathogens.

Historically, arboviruses and other neglected tropical diseases have been understudied because the burden of disease falls on under resourced populations in the Global South[115] In the context of ZIKV, the unequal distribution of risk is coupled with inequities in access to preventative measures like modern contraception and to critical clinical and therapeutic care for infants affected by microcephaly and ZIKV-related neurological disorders. Each case of microcephaly is associated with a loss of 29.95 DALYs and treatment costs ranging from 91K to 1 million USD.[116] To put these figures into perspective, the yearly

For peer review only - http://bmjopen.bmj.com/site/about/guidelines.xhtml

per capita income in Pernambuco, the Brazilian state with one of the highest burdens of CZS, is 3,471 USD.[117]

There is no vaccine for ZIKV and the only treatment is supportive.[58] There have been numerous calls for data sharing[118 119] and cooperation between governments and academic institutions,[54 120] and public and private charities have pledged significant financial support to improve our understanding of ZIKV epidemiology and to develop a vaccine or small molecule prophylaxis to decrease the risk of infection. In the wake of the Ebola epidemic, the global response to ZIKV has been characterized by unprecedented levels of international cooperation. In the absence of a ZIKV vaccine or prophylaxis, international leaders in ZIKV research have formed the ZIKV IPD Consortium to identify, collect, and synthesize IPD from longitudinal studies of pregnant women that measure ZIKV infection during pregnancy and fetal, infant, and child outcomes. This data will be used to quantify the absolute risk of ZIKV-related pregnancy complications with the goal of aiding women and their families in making difficult reproductive decisions and with helping public health systems prevent and quantify the burden of congenital Zika infection.

Challenges of developing and conducting an individual participant data-meta-analysis in the context of an emerging pathogen

Ideally, researchers pre-specify confounders, effect measure modifiers and plans for subgroup or sensitivity analyses in their research protocol. In the context of Zika, our understanding of the virus is changing so rapidly that analysis plans may change significantly despite our best efforts to review the latest evidence on transmission, immunological response, diagnostic assays, vector biology, and basic ZIKV epidemiology. Our ability to appropriately account for measurement error will play a critical role in the accuracy of estimates for the risk of CZS and other adverse fetal, infant, and child outcomes. This is one of the first instances where an IPD-MA has been used to address public health concerns in the context of an emerging pathogen. We expect that best practices and lessons learned from this IPD-MA can be used to facilitate the formation of research collaborations to streamline the public health response to future epidemics.

Patient and Public Involvement

In keeping with guidelines for public involvement in research,[121] knowledge users (i.e. women of reproductive age and their families, clinicians) will be consulted at each stage of this research. The research question and protocol were designed with feedback from clinicians who treat pregnant women in ZIKV-endemic areas and infants and children affected by CZS. Focus groups that include women of reproductive age in ZIKV-endemic areas will be used to evaluate the ethical implications and utility of the risk stratification tool in three countries.

ETHICS AND DISSEMINATION

This IPD-MA protocol has been deemed exempt from ethical review by the WHO Ethics Review Committee and the Emory University Institutional Review Board. Individual longitudinal studies will

For peer review only - http://bmjopen.bmj.com/site/about/guidelines.xhtml

provide documentation of ethics review prior to sharing their de-identified, participant-level data. The WHO has developed guidance for data sharing in public health emergencies or in the context of emerging pathogens.[122] Sharing de-identified data for IPD-MA is generally considered exempt from ethical review if the objectives of the IPD-MA are in keeping with the objectives of the original studies.[123] Individual research studies and consortia will secure additional ethics review and/or legal guidance on the sharing of de-identified, subject-level data as needed. The results of this analysis will be published under the ZIKV IPD Consortium name and will include a list of the names of key investigators from each study that contributed data for that analysis and researchers who contributed to the analysis or writing at the end of the publication. Findings from the proposed analysis will be shared via national and international conferences; existing platforms for dissemination of ZIKV-related research (e.g. The Global Health Network); and through publication in open access, peer-reviewed journals.

Contributors

NB, CBH, TJ, NL, LM, JPS, LR contributed to the initial conception of the study. AB, TD, PG, NL, LM, YW made substantial contributions to the statistical methodology proposed for the IPD-MA. LM wrote the first draft of the manuscript. All authors revised the manuscript and approved the final version of the manuscript.

Acknowledgments

The authors would like to acknowledge the valuable contributions of Rosangela Batista, Ana Paula Bertozzi, Gabriel Carles, María Manoela Duarte Rodrigues, Cassia F. Estofolete, Maria Isabel Fragoso da Silveira Gouvêa, Vicky Fumadó-Pérez, Rosa Estela Gazeta, Neely Kaydos-Daniels, Suzanne Gilboa, Amy Krystosik, Véronique Lambert, Milagros García López-Hortelano, Christina Nelson, Denise M. Oliani, Marizelia Ribeiro, Barry Rockx, Laura C. Rodrigues, Katia Silveira, Elena Sulleiro, Van Tong, Diana Valencia, Wayner Vieira de Souza, Luis Angel Villar Centeno, and Andrea Zin to the review of the ZIKV IPD Consortium IPD-MA Protocol.

Funding

The development of the IPD-MA protocol was supported by a Wellcome Trust grant to the WHO Department of Reproductive Health and Research Human Reproduction Programme, grant number 206532/Z/17/Z.

Disclaimer

The manuscript contents are the responsibility of the authors and do not reflect the views of the Wellcome Trust.

Competing Interests

None declared

Provenance and peer review

Not commissioned; externally peer reviewed.

Data sharing statement

For peer review only - http://bmjopen.bmj.com/site/about/guidelines.xhtml

Not all investigators are willing to share study for analyses beyond what has been proposed here. Governance issues related to sharing the de-identified, participant-level data used in the proposed analyses will be described in the manuscripts that present the results of the proposed analyses.

References

1. Krauer F, Riesen M, Reveiz L, et al. Zika Virus Infection as a Cause of Congenital Brain Abnormalities and Guillain–Barré Syndrome: Systematic Review. *PLOS Medicine* 2017;14(1):e1002203. doi: 10.1371/journal.pmed.1002203
2. Rasmussen SA, Jamieson DJ, Honein MA, et al. Zika Virus and Birth Defects — Reviewing the Evidence for Causality. *New England Journal of Medicine* 2016;374(20):1981-87. doi: doi:10.1056/NEJMsr1604338
3. Bhatnagar J, Rabeneck D, Martines R, et al. Zika Virus RNA Replication and Persistence in Brain and Placental Tissue. *Emerging Infectious Diseases* 2017;23(3)
4. Garcez PP, Loiola EC, Madeiro da Costa R, et al. Zika virus impairs growth in human neurospheres and brain organoids. *Science* 2016 doi: 10.1126/science.aaf6116
5. Dang J, Tiwari SK, Lichinchi G, et al. Zika Virus Depletes Neural Progenitors in Human Cerebral Organoids through Activation of the Innate Immune Receptor TLR3. *Cell Stem Cell* 2016;19(2):258-65. doi: https://doi.org/10.1016/j.stem.2016.04.014
6. Tang H, Hammack C, Ogden Sarah C, et al. Zika Virus Infects Human Cortical Neural Progenitors and Attenuates Their Growth. *Cell Stem Cell* 2016;18(5):587-90. doi: https://doi.org/10.1016/j.stem.2016.02.016
7. Opitz J, Holt M. Microcephaly: general considerations and aids to nosology. *Journal of craniofacial genetics and developmental biology* 1990;10(2):175-204.
8. Passemard S, Kaindl AM, Verloes A. Microcephaly. Handbook of clinical neurology: Elsevier 2013:129-41.
9. Melo A, Aguiar R, Amorim M, et al. Congenital zika virus infection: Beyond neonatal microcephaly. *JAMA Neurology* 2016 doi: 10.1001/jamaneurol.2016.3720
10. Coyne CB, Lazear HM. Zika virus [mdash] reigniting the TORCH. *Nat Rev Micro* 2016;14(11):707-15. doi: 10.1038/nrmicro.2016.125
11. Kaindl AM, Passemard S, Kumar P, et al. Many roads lead to primary autosomal recessive microcephaly. *Progress in Neurobiology* 2010;90(3):363-83. doi: http://dx.doi.org/10.1016/j.pneurobio.2009.11.002
12. Chan JFW, Choi GKY, Yip CCY, et al. Zika fever and congenital Zika syndrome: An unexpected emerging arboviral disease. *Journal of Infection* 2016;72(5):507-24. doi: http://dx.doi.org/10.1016/j.jinf.2016.02.011
13. de Araújo TVB, Ximenes RAdA, Miranda-Filho DdB, et al. Association between microcephaly, Zika virus infection, and other risk factors in Brazil: final report of a case-control study. *The Lancet Infectious Diseases* 2017 doi: https://doi.org/10.1016/S1473-3099(17)30727-2
14. Krow-Lucal ER, de Andrade MR, Cananéa JNA, et al. Association and birth prevalence of microcephaly attributable to Zika virus infection among infants in Paraíba, Brazil, in 2015–16: a case-control study. *The Lancet Child & Adolescent Health* 2018 doi: https://doi.org/10.1016/S2352-4642(18)30020-8
15. Brasil P, Pereira JPJ, Moreira ME, et al. Zika Virus Infection in Pregnant Women in Rio de Janeiro. *New England Journal of Medicine* 2016;375(24):2321-34. doi: doi:10.1056/NEJMoa1602412

For peer review only - http://bmjopen.bmj.com/site/about/guidelines.xhtml

16. Hoen B, Schaub B, Funk AL, et al. Pregnancy Outcomes after ZIKV Infection in French Territories in the Americas. *New England Journal of Medicine* 2018;378(11):985-94. doi: 10.1056/NEJMoa1709481
17. Honein MA, Dawson AL, Petersen EE, et al. Birth defects among fetuses and infants of us women with evidence of possible zika virus infection during pregnancy. *JAMA* 2017;317(1):59-68. doi: 10.1001/jama.2016.19006
18. Osuna CE, Whitney JB. Nonhuman Primate Models of Zika Virus Infection, Immunity, and Therapeutic Development. *The Journal of Infectious Diseases* 2017;216(suppl_10):S928-S34. doi: 10.1093/infdis/jix540
19. Musso D, Cao-Lormeau VM, Gubler DJ. Zika virus: following the path of dengue and chikungunya? *The Lancet* 2015;386(9990):243-44. doi: 10.1016/S0140-6736(15)61273-9
20. Araujo LM, Ferreira MLB, Nascimento OJ. Guillain-Barré syndrome associated with the Zika virus outbreak in Brazil. *Arquivos de Neuro-Psiquiatria* 2016;74:253-55.
21. Parra B, Lizarazo J, Jiménez-Arango JA, et al. Guillain–Barré Syndrome Associated with Zika Virus Infection in Colombia. *New England Journal of Medicine* 2016;375(16):1513-23. doi: 10.1056/NEJMoa1605564
22. Pacheco O, Beltrán M, Nelson CA, et al. Zika Virus Disease in Colombia — Preliminary Report. *New England Journal of Medicine* 2016;0(0) doi: 10.1056/NEJMoa1604037
23. Cauchemez S, Besnard M, Bompard P, et al. Association between Zika virus and microcephaly in French Polynesia, 2013–15: a retrospective study. *The Lancet* 2016;387(10033):2125-32. doi: http://dx.doi.org/10.1016/S0140-6736(16)00651-6
24. Cao-Lormeau V-M, Blake A, Mons S, et al. Guillain-Barré Syndrome outbreak associated with Zika virus infection in French Polynesia: a case-control study. *The Lancet* 2016;387(10027):1531-39. doi: http://dx.doi.org/10.1016/S0140-6736(16)00562-6
25. Epidemiological Alert: Zika virus infection: Pan American Health Organization, 2016:1-8.
26. Lowe R, Barcellos C, Brasil P, et al. The Zika Virus Epidemic in Brazil: From Discovery to Future Implications. *International Journal of Environmental Research and Public Health* 2018;15(1):96. doi: 10.3390/ijerph15010096
27. Zika virus infection: global update on epidemiology and potentially associated clinical manifestations. Wkly Epidemiol Rec: WHO Press, 2016:73-81.
28. Cruz MM, Puerta-Guardo HN, Schildhauer S, et al. Immune cross-reactivity between Dengue and Zika viruses in two pediatric studies in Nicaragua. *The Journal of Immunology* 2017;198(1 Supplement):210.5-10.5.
29. Castanha PMS, Nascimento EJM, Braga C, et al. Dengue Virus–Specific Antibodies Enhance Brazilian Zika Virus Infection. *The Journal of Infectious Diseases* 2017;215(5):781-85. doi: 10.1093/infdis/jiw638
30. Goncalves A, Peeling RW, Chu MC, et al. Innovative and New Approaches to Laboratory Diagnosis of Zika and Dengue: A Meeting Report. *The Journal of Infectious Diseases* 2017:jix678-jix78. doi: 10.1093/infdis/jix678
31. Turner LH, Kinder JM, Wilburn A, et al. Preconceptual Zika virus asymptomatic infection protects against secondary prenatal infection. *PLoS Pathogens* 2017;13(11):e1006684. doi: 10.1371/journal.ppat.1006684
32. Andrade DV, Harris E. Recent advances in understanding the adaptive immune response to Zika virus and the effect of previous flavivirus exposure. *Virus Research* 2017 doi: https://doi.org/10.1016/j.virusres.2017.06.019
33. Andrade PE, Coloma J, Michlmayr D, et al. Zika and Dengue Virus Specific and Cross-reactive Memory B Cell Responses. *The Journal of Immunology* 2017;198(1 Supplement):214.7-14.7.

For peer review only - http://bmjopen.bmj.com/site/about/guidelines.xhtml

34. Jaenisch T, Rosenberger KD, Brito C, et al. Estimating the risk for microcephaly after Zika virus infection in Brazil. *Bulletin of the World Health Organization* 2016 doi: http://dx.doi.org/10.2471/BLT.16.178608
35. Butler D. First Zika-linked birth defects detected in Colombia. *Nature* 2016;531(7593):153.
36. Costa F, Ko AI. Zika virus and microcephaly: where do we go from here? *The Lancet Infectious Diseases* 2018;18(3):236-37. doi: https://doi.org/10.1016/S1473-3099(17)30697-7
37. YKY C, TY L, TTH L, et al. Impact of replacing Chinese ethnicity-specific fetal biometry charts with the INTERGROWTH-21st standard. *BJOG: An International Journal of Obstetrics & Gynaecology* 2016;123(S3):48-55. doi: doi:10.1111/1471-0528.14008
38. Leibovitz Z, Daniel-Spiegel E, Malinger G, et al. Prediction of microcephaly at birth using three reference ranges for fetal head circumference: can we improve prenatal diagnosis? *Ultrasound in Obstetrics & Gynecology* 2016;47(5):586-92. doi: doi:10.1002/uog.15801
39. Barchfield J. Officials in Brazil urge women to avoid pregnancy due to Zika virus: Boston Globe; 2016 [updated January 26, 2016. Available from: https://www.bostonglobe.com/news/world/2016/01/26/officials-brazil-urge-women-avoid-pregnancy-due-zika-virus/Ask7wAjozV0G6SCv80uK8N/story.html accessed June 4, 2018 2018.
40. Brodzinsky S. Rights groups denounce Zika advice to avoid pregnancy in Latin America London, England: Guardian; 2016 [updated January 27, 2016. Available from: https://www.theguardian.com/global-development/2016/jan/27/rights-groups-denounce-zika-advice-to-avoid-pregnancy-in-latin-america accessed June 4, 2018 2018.
41. Wilder-Smith A, Gubler DJ, Weaver SC, et al. Epidemic arboviral diseases: priorities for research and public health. *The Lancet Infectious Diseases* 2017;17(3):e101-e06. doi: https://doi.org/10.1016/S1473-3099(16)30518-7
42. Aiken ARA, Scott JG, Gomperts R, et al. Requests for Abortion in Latin America Related to Concern about Zika Virus Exposure. *New England Journal of Medicine* 2016;375(4):396-98. doi: 10.1056/NEJMc1605389
43. Stern AM. Zika and reproductive justice. *Cadernos de Saúde Pública* 2016;32
44. Debray TPA, Riley RD, Rovers MM, et al. Individual Participant Data (IPD) Meta-analyses of Diagnostic and Prognostic Modeling Studies: Guidance on Their Use. *PLoS Med* 2015;12(10):e1001886. doi: 10.1371/journal.pmed.1001886
45. Riley RD, Ensor J, Snell KIE, et al. External validation of clinical prediction models using big datasets from e-health records or IPD meta-analysis: opportunities and challenges. *BMJ* 2016;353 doi: 10.1136/bmj.i3140
46. Weaver SC, Costa F, Garcia-Blanco MA, et al. Zika virus: History, emergence, biology, and prospects for control. *Antiviral Research* 2016;130:69-80. doi: http://dx.doi.org/10.1016/j.antiviral.2016.03.010
47. Alphey L, Benedict M, Bellini R, et al. Sterile-insect methods for control of mosquito-borne diseases: an analysis. *Vector-Borne and Zoonotic Diseases* 2010;10(3):295-311.
48. Yakob L, Walker T. Zika virus outbreak in the Americas: the need for novel mosquito control methods. *The Lancet Global Health* 2016;4(3):e148-e49. doi: http://dx.doi.org/10.1016/S2214-109X(16)00048-6
49. Fernandes M, Stein A, Newton CR, et al. The INTERGROWTH-21st Project Neurodevelopment Package: A Novel Method for the Multi-Dimensional Assessment of Neurodevelopment in Pre-School Age Children. *PLOS ONE* 2014;9(11):e113360. doi: 10.1371/journal.pone.0113360
50. Riley RD, Lambert PC, Staessen JA, et al. Meta-analysis of continuous outcomes combining individual patient data and aggregate data. *Statistics in Medicine* 2008;27(11):1870-93. doi: 10.1002/sim.3165

For peer review only - http://bmjopen.bmj.com/site/about/guidelines.xhtml

51. Debray TPA, Moons KGM, Abo-Zaid GMA, et al. Individual Participant Data Meta-Analysis for a Binary Outcome: One-Stage or Two-Stage? *PLoS ONE* 2013;8(4):e60650. doi: 10.1371/journal.pone.0060650
52. Debray TPA, Moons KGM, van Valkenhoef G, et al. Get real in individual participant data (IPD) meta-analysis: a review of the methodology. *Research Synthesis Methods* 2015;6(4):293-309. doi: 10.1002/jrsm.1160
53. Morabito KM, Graham BS. Zika Virus Vaccine Development. *The Journal of Infectious Diseases* 2017;216(suppl_10):S957-S63. doi: 10.1093/infdis/jix464
54. Hotez PJ. What does zika virus mean for the children of the Americas? *JAMA Pediatrics* 2016;170(8):787-89. doi: 10.1001/jamapediatrics.2016.1465
55. Dejnirattisai W, Supasa P, Wongwiwat W, et al. Dengue virus sero-cross-reactivity drives antibody-dependent enhancement of infection with zika virus. *Nat Immunol* 2016;17(9):1102-08. doi: 10.1038/ni.3515
56. Malone RW, Homan J, Callahan MV, et al. Zika Virus: Medical Countermeasure Development Challenges. *PLoS Negl Trop Dis* 2016;10(3):e0004530. doi: 10.1371/journal.pntd.0004530
57. Bardina SV, Bunduc P, Tripathi S, et al. Enhancement of Zika virus pathogenesis by preexisting antiflavivirus immunity. *Science* 2017;356(6334):175-80. doi: 10.1126/science.aal4365
58. Poland GA, Kennedy RB, Ovsyannikova IG, et al. Development of vaccines against Zika virus. *The Lancet Infectious Diseases* 2018;18(7):e211-e19. doi: 10.1016/S1473-3099(18)30063-X
59. Durbin AP, Whitehead SS. Zika Vaccines: Role for Controlled Human Infection. *The Journal of Infectious Diseases* 2017;216(suppl_10):S971-S75. doi: 10.1093/infdis/jix491
60. Van Kerkhove MD, Reveiz L, Souza JP, et al. Harmonisation of Zika virus research protocols to address key public health concerns. *The Lancet Global Health* 2016;4(12):e911-e12. doi: 10.1016/S2214-109X(16)30255-8
61. Moher D, Shamseer L, Clarke M, et al. Preferred reporting items for systematic review and meta-analysis protocols (PRISMA-P) 2015 statement. *Systematic Reviews* 2015;4(1):1.
62. Stewart LA, Clarke M, Rovers M, et al. Preferred reporting items for a systematic review and meta-analysis of individual participant data: The prisma-ipd statement. *JAMA* 2015;313(16):1657-65. doi: 10.1001/jama.2015.3656
63. Reveiz L, Haby MM, Martínez-Vega R, et al. Risk of bias and confounding of observational studies of Zika virus infection: A scoping review of research protocols. *PLOS ONE* 2017;12(7):e0180220. doi: 10.1371/journal.pone.0180220
64. Counotte M, Egli-Gany D, Riesen M, et al. Zika virus infection as a cause of congenital brain abnormalities and Guillain-Barré syndrome: From systematic review to living systematic review [version 1; referees: 1 approved with reservations]2018.
65. Jupyter Notebooks-a publishing format for reproducible computational workflows. ELPUB; 2016.
66. Kiserud T, Piaggio G, Carroli G, et al. The World Health Organization fetal growth charts: a multinational longitudinal study of ultrasound biometric measurements and estimated fetal weight. *PLoS Medicine* 2016:1-79.
67. Fenton TR, Kim JH. A systematic review and meta-analysis to revise the Fenton growth chart for preterm infants. *BMC Pediatrics* 2013;13(1):59. doi: 10.1186/1471-2431-13-59
68. Silverman JG, Decker MR, Reed E, et al. Intimate partner violence victimization prior to and during pregnancy among women residing in 26 U.S. states: Associations with maternal and neonatal health. *American Journal of Obstetrics and Gynecology* 2006;195(1):140-48. doi: https://doi.org/10.1016/j.ajog.2005.12.052
69. Bayley N. Bayley scales of infant development: Manual: Psychological Corporation 1993.
70. Squires J, Bricker DD, Twombly E. Ages & stages questionnaires: Paul H. Brookes Baltimore, MD 2009.

For peer review only - http://bmjopen.bmj.com/site/about/guidelines.xhtml

71. Reeves BC, Deeks JJ, Higgins JP. Chapter 13: Including non-randomized studies. In: Higgins JG, S, ed. Cochrane handbook for systematic reviews of interventions. Chichester (UK): John Wiley & Sons 2008.
72. Jarde A, Losilla J-M, Vives J, et al. Q-Coh: A tool to screen the methodological quality of cohort studies in systematic reviews and meta-analyses. *International Journal of Clinical and Health Psychology* 2013;13(2):138-46. doi: http://dx.doi.org/10.1016/S1697-2600(13)70017-6
73. Higgins J, Sterne J, Savović J, et al. A revised tool for assessing risk of bias in randomized trials. *Cochrane Database Syst Rev* 2016;10(Suppl 1):29-31.
74. Zika Virus: Information for Clinicians Atlanta, GA: Centers for Disease Control and Prevention; 2016 [updated 13 June 2016; cited 2016. Available from: https://www.cdc.gov/zika/pdfs/clinicianppt.pdf accessed 29 December 2016 2016.
75. Gómez G, Espinal A, W. Lagakos S. Inference for a linear regression model with an interval-censored covariate. *Statistics in Medicine* 2003;22(3):409-25. doi: 10.1002/sim.1326
76. Cortina-Borja M, Tan HK, Wallon M, et al. Prenatal Treatment for Serious Neurological Sequelae of Congenital Toxoplasmosis: An Observational Prospective Cohort Study. *PLOS Medicine* 2010;7(10):e1000351. doi: 10.1371/journal.pmed.1000351
77. Marx A, Bucher HC. Numbers needed to treat derived from meta-analysis: a word of caution. *Evidence Based Medicine* 2003;8(2):36-37. doi: 10.1136/ebm.8.2.36
78. Schulz KF, Altman DG, Moher D. CONSORT 2010 Statement: updated guidelines for reporting parallel group randomised trials. *BMC Medicine* 2010;8(1):1-9. doi: 10.1186/1741-7015-8-18
79. Riley RD, Higgins JP, Deeks JJ. Interpretation of random effects meta-analyses. *BMJ* 2011;342 doi: 10.1136/bmj.d549
80. Yelland LN, Salter AB, Ryan P. Performance of the Modified Poisson Regression Approach for Estimating Relative Risks From Clustered Prospective Data. *American Journal of Epidemiology* 2011;174(8):984-92. doi: 10.1093/aje/kwr183
81. Richardson DB, Kinlaw AC, MacLehose RF, et al. Standardized binomial models for risk or prevalence ratios and differences. *International journal of epidemiology* 2015;44(5):1660-72. doi: https://doi.org/10.1093/ije/dyv137
82. Wood SN. Low-Rank Scale-Invariant Tensor Product Smooths for Generalized Additive Mixed Models. *Biometrics* 2006;62(4):1025-36. doi: doi:10.1111/j.1541-0420.2006.00574.x
83. Riley RD, Price MJ, Jackson D, et al. Multivariate meta-analysis using individual participant data. *Research Synthesis Methods* 2015;6(2):157-74. doi: 10.1002/jrsm.1129
84. Wei Y, Higgins JPT. Bayesian multivariate meta-analysis with multiple outcomes. *Statistics in Medicine* 2013;32(17):2911-34. doi: doi:10.1002/sim.5745
85. Higgins J, Deeks JJ, Altman DG. 16.9.1 Meta-analysis of rare events. Cochrane handbook for systematic reviews of interventions: Cochrane book series2008:481-529.
86. Stijnen T, Hamza TH, Özdemir P. Random effects meta-analysis of event outcome in the framework of the generalized linear mixed model with applications in sparse data. *Statistics in Medicine* 2010;29(29):3046-67. doi: 10.1002/sim.4040
87. Tierney JF, Vale C, Riley R, et al. Individual Participant Data (IPD) Meta-analyses of Randomised Controlled Trials: Guidance on Their Use. *PLOS Medicine* 2015;12(7):e1001855. doi: 10.1371/journal.pmed.1001855
88. Fisher DJ, Copas AJ, Tierney JF, et al. A critical review of methods for the assessment of patient-level interactions in individual participant data meta-analysis of randomized trials, and guidance for practitioners. *J Clin Epidemiol* 2011;64(9):949-67. doi: 10.1016/j.jclinepi.2010.11.016 [published Online First: 2011/03/16]
89. Knol MJ, VanderWeele TJ. Recommendations for presenting analyses of effect modification and interaction. *International Journal of Epidemiology* 2012;41(2):514-20. doi: 10.1093/ije/dyr218

For peer review only - http://bmjopen.bmj.com/site/about/guidelines.xhtml

90. Koopman L, van der Heijden GJMG, Hoes AW, et al. Empirical comparison of subgroup effects in conventional and individual patient data meta-analyses. *International Journal of Technology Assessment in Health Care* 2008;24(3):358-61. doi: 10.1017/S0266462308080471
91. VanderWeele TJ. Marginal Structural Models for the Estimation of Direct and Indirect Effects. *Epidemiology* 2009;20(1):18-26 10.1097/EDE.0b013e31818f69ce.
92. VanderWeele TJ, Tchetgen Tchetgen EJ. Attributing effects to interactions. *Epidemiology (Cambridge, Mass)* 2014;25(5):711-22. doi: 10.1097/EDE.0000000000000096
93. Groll A, Groll MA. Package 'glmmLasso'. 2016
94. Yuan M, Lin Y. Model selection and estimation in regression with grouped variables. *Journal of the Royal Statistical Society: Series B (Statistical Methodology)* 2006;68(1):49-67. doi: 10.1111/j.1467-9868.2005.00532.x
95. Meier L, Van De Geer S, Bühlmann P. The group lasso for logistic regression. *Journal of the Royal Statistical Society: Series B (Statistical Methodology)* 2008;70(1):53-71. doi: 10.1111/j.1467-9868.2007.00627.x
96. Groll A, Tutz G. Variable selection for generalized linear mixed models by L 1-penalized estimation. *Statistics and Computing* 2014;24(2):137-54. doi: 10.1007/s11222-012-9359-z
97. Debray TPA, Moons KGM, Ahmed I, et al. A framework for developing, implementing, and evaluating clinical prediction models in an individual participant data meta-analysis. *Statistics in Medicine* 2013;32(18):3158-80. doi: 10.1002/sim.5732
98. Ahmed I, Debray TP, Moons KG, et al. Developing and validating risk prediction models in an individual participant data meta-analysis. *BMC Medical Research Methodology* 2014;14(1):3. doi: 10.1186/1471-2288-14-3
99. Hanley JA, McNeil BJ. The meaning and use of the area under a receiver operating characteristic (ROC) curve. *Radiology* 1982;143(1):29-36. doi: doi:10.1148/radiology.143.1.7063747
100. Steyerberg EW, Vickers AJ, Cook NR, et al. Assessing the performance of prediction models: a framework for some traditional and novel measures. *Epidemiology (Cambridge, Mass)* 2010;21(1):128-38. doi: 10.1097/EDE.0b013e3181c30fb2
101. Royston P, Parmar MKB, Sylvester R. Construction and validation of a prognostic model across several studies, with an application in superficial bladder cancer. *Statistics in Medicine* 2004;23(6):907-26. doi: doi:10.1002/sim.1691
102. Moons KM, Altman DG, Reitsma JB, et al. Transparent reporting of a multivariable prediction model for individual prognosis or diagnosis (tripod): Explanation and elaboration. *Annals of Internal Medicine* 2015;162(1):W1-W73. doi: 10.7326/M14-0698
103. Balshem H, Helfand M, Schünemann HJ, et al. GRADE guidelines: 3. Rating the quality of evidence. *Journal of clinical epidemiology* 2011;64(4):401-06.
104. Hernán MA, Hernandez-Diaz S, Robins JM. A structural approach to selection bias. *Epidemiology* 2004;15(5):615-25.
105. Hansen BB. The prognostic analogue of the propensity score. *Biometrika* 2008;95(2):481-88. doi: 10.1093/biomet/asn004
106. Mansournia MA, Etminan M, Danaei G, et al. Handling time varying confounding in observational research. *BMJ* 2017;359 doi: 10.1136/bmj.j4587
107. Sorvillo FJ, Morrison AC, Berlin OGW. Vector-Borne Transmission. In: Thomas JC, Weber DJ, eds. Epidemiologic methods for the study of infectious diseases. Oxford: Oxford University Press 2001:249-65.
108. Balmaseda A, Zambrana JV, Collado D, et al. Comparison of four serological methods and two RT-PCR assays for diagnosis and surveillance of Zika. *Journal of Clinical Microbiology* 2018 doi: 10.1128/JCM.01785-17

For peer review only - http://bmjopen.bmj.com/site/about/guidelines.xhtml

109. Screening, assessment and management of neonates and infants with complications associated with Zika virus exposure in utero: Interim guidance. 2016 30 August 2016. http://origin.who.int/csr/resources/publications/zika/assessment-infants/en/ (accessed 17 January 2017).
110. Gelman A, Carlin JB, Stern HS, et al. Bayesian data analysis: CRC press Boca Raton, FL 2014.
111. Riley RD, Lambert PC, Abo-Zaid G. Meta-analysis of individual participant data: rationale, conduct, and reporting. *BMJ: British Medical Journal* 2010;340(7745):521-25. doi: 10.2307/25674217
112. Burgess S, White IR, Resche-Rigon M, et al. Combining multiple imputation and meta-analysis with individual participant data. *Statistics in Medicine* 2013;32(26):4499-514. doi: 10.1002/sim.5844
113. Audigier V, White IR, Jolani S, et al. Multiple imputation for multilevel data with continuous and binary variables. *arXiv preprint arXiv:170200971* 2017
114. Peters JL, Sutton AJ, Jones DR, et al. Comparison of two methods to detect publication bias in meta-analysis. *JAMA* 2006;295(6):676-80. doi: 10.1001/jama.295.6.676
115. Hotez PJ, Fenwick A, Savioli L, et al. Rescuing the bottom billion through control of neglected tropical diseases. *The Lancet* 2009;373(9674):1570-75. doi: http://dx.doi.org/10.1016/S0140-6736(09)60233-6
116. Alfaro-Murillo JA, Parpia AS, Fitzpatrick MC, et al. A Cost-Effectiveness Tool for Informing Policies on Zika Virus Control. *PLoS Negl Trop Dis* 2016;10(5):e0004743. doi: 10.1371/journal.pntd.0004743
117. Pernambuco: Wikipedia; 2017 [updated 14 January 2017. Available from: https://en.wikipedia.org/wiki/Pernambuco accessed 17 January 2017 2017.
118. Sharing data during Zika and other global health emergencies: Wellcome Trust; 2016 [updated 10 February 2016. Available from: https://wellcome.ac.uk/news/sharing-data-during-zika-and-other-global-health-emergencies accessed 10 December 2016 2016.
119. Gérardin P, Randrianaivo H, Schaub B, et al. Congenital Zika syndrome: time to move from case series to case-control studies and data sharing. *BMJ* 2016;354 doi: 10.1136/bmj.i4850
120. The next steps on Zika. *Nature* 2016;530:5.
121. Popay J, Collins M, Group wtPS. The public involvement impact assessment framework guidance. *Universities of Lancaster, Liverpool and Exeter* 2014
122. Modjarrad K, Moorthy VS, Millett P, et al. Developing Global Norms for Sharing Data and Results during Public Health Emergencies. *PLOS Medicine* 2016;13(1):e1001935. doi: 10.1371/journal.pmed.1001935
123. Menikoff J. Letter from Jerry Menikoff, MD, JD, Director, Office for Human Research Protections, to ICMJE Secretariat. 7 March 2017, 2017.

For peer review only - http://bmjopen.bmj.com/site/about/guidelines.xhtml

Supplementary Table 1. List of longitudinal research studies and active surveillance programs that have agreed to contribute participant-level data to the ZIKV Consortium individual participant data meta-analysis of longitudinal studies of pregnant women and their infants and children

Country	City	Study Name	Coordinating Center(s)	Consortium Name*
Brazil	Campina Grande	Freqüência e evolução dos achados ultrassonograficos e de ressonäncia magnética em fetos de mães com sintomas de Zika virus e a associação com desfechos neonatais em Campina Grande - Paraíba: Estudo de coorte	Instituto do Cérebro, Rio de Janeiro; Instituto D´Or, Rio de Janeiro	
Brazil	Goiânia	Cohort of Pregnant women with rash from Goiânia, Goiás State, Brazil and Cohort of children vertically exposed to Zika virus in Goiania	Institute of Tropical Pathology and Public Health<br><br>Federal University of Goiás, Brazil	ZikaPLAN
Brazil	Jundiaí	Infecção Vertical pelo vírus ZIKA e suas repercussões na área materno-infantil	Faculdade de Medicina de Jundiaí	
Brazil	São Luís, Maranhão	Monitoramento da microcefalia em recém-nascidos e acompanhamento clínico e de crescimento e desenvolvimento de uma coorte de crianças com provável infecção congênita pelo virus da Zika	Hospital Universitário da Universidade Federal do Maranhão/HU/UFMA	

For peer review only

For peer review only - http://bmjopen.bmj.com/site/about/guidelines.xhtml

Country	City	Study Name	Coordinating Center(s)	Consortium Name*
Brazil	Metropolitan region of Recife, Pernambuco	Coorte de gestantes com exantema no estado de Pernambuco	Universidade Federal de Pernambuco and Centro de Pesquisas Aggeu Magalhães-Fiocruz-PE	MERG/Fiocruz, ZikaPLAN
Brazil	Pernambuco	Coorte de gestanes com exantema no estado de Pernambuco	Fundação Oswaldo Cruz (Fiocruz)	MERG/Fiocruz, ZikaPlan
Brazil	Pernambuco	Coorte clínica de crianças com microcefalia em Pernambuco	Universidade Federal de Pernambuco and Centro de Pesquisas Aggeu Magalhães-Fiocruz-PE	MERG/Fiocruz, ZikaPlan
Brazil	Ribeirão Preto	Natural history of Zika virus infection in pregnant and consequences for pregnancy, fetus and child (Zika Project in Pregnancy - ZIG)	Universidade de São Paulo	
Brazil	Rio de Janeiro	Infecção pelo vírus Zika em uma coorte de gestantes e seus conceptos	Maternidade Escola da Universidade Federal do Rio de Janeiro	
Brazil	Rio de Janeiro	Estudo de coorte de gestantes e criancas expostas e infectadas intrautero pelo Zika virus	Instituto de Puericultura e Pediatria Martagão Gesteira, Rio de Janeiro; Hospital Universitário Pedro Ernesto	
Brazil	Rio de Janeiro	Zika Virus Infection in Pregnant Women in Rio de Janeiro	Fundação Oswaldo Cruz (Fiocruz), Rio de Janeiro	Fiocruz
Brazil	Rio de Janeiro	Zika virus coinfection among HIV infected pregnant women in a Brazilian cohort	Hospital dos Servidores do Estado	

For peer review only - http://bmjopen.bmj.com/site/about/guidelines.xhtml

Country	City	Study Name	Coordinating Center(s)	Consortium Name*
Brazil	São José do Rio Preto	Diagnóstico de arboviroses brasileiras e emergentes em pacientes e mosquitos em duas regiões distintas do Brasil	Faculdade de Medicina de São José do Rio Preto, Secretaria de Desenvolvimento, Econômico, Ciência e Tecnologia, São Paulo State	
Brazil	Vitoria	Epidemia de Zika virus no estado do Espirito Santo: estudo de impacto da infeccao sobre o feto em uma coorte de gestantes, com sintomas da doenca e confirmacao virologica da infeccao	Hospital Universitário Cassiano Antônio de Moraes	
Brazil<br>Colombia<br>Guatemala<br>Nicaragua<br>Puerto Rico<br>Mexico		Zika in Infants and Pregnancy (ZIP)	RTI International; Eunice Kennedy Shriver National Institute of Child Health and Human Development; National Institute of Allergy and Infectious Disease, National Institute of Environmental Health Sciences; Fundação Oswaldo Cruz (Fiocruz)	NIH/NIAID
Colombia	Baranquilla, Soledad, Bucaramanga, Tuluá	Zika en Embarazadas y Niños (ZEN)		CDC/INS
Colombia	Santander	Neurodevelopment outcome of newborns exposed to Zika virus in utero (ZEN)	UNC-CH, Michigan State University, Universidad Industrial de Santander	

For peer review only - http://bmjopen.bmj.com/site/about/guidelines.xhtml

Country	City	Study Name	Coordinating Center(s)	Consortium Name*
Colombia	Barranquilla<br>Cali<br>Cúcuta	Vigilancia de Embarazadas con Zika (VEZ; Surveillance cohort)		CDC
Ecuador<br>Cuba<br>Mexico (IMSS, MOH)<br>Venezuela: Valencia<br>Brazil: Fortaleza, Recife, Rio de Janeiro<br>Colombia: Bucaramanga		Pregnant Women Cohort for evaluation of absolute and relative risk of congenital malformations after Zika virus infection – developmental milestones of children born to women exposed to Zika virus during pregnancy	Heidelberg University	ZIKAlliance, Fiocruz, IDAMS
Grenada		The Spectrum of Zika Disease in Grenada - Pregnancy Cohort	St. George's University, Stanford University, Windward Islands Research and Education Foundation	
Guadaloupe, Martinique, French Guyana, St Martin		Zika Virus Infection's Pregnancy Consequences in French Department of America (ZIKA-DFA-FE)		INSERM
French Guyana		Zika Virus Infection's Neonatal and Pediatric Consequences in French Department of America (ZIKA-DFA-BB)		INSERM
Honduras		Zika Virus Infection in Pregnant Women in Honduras (ZIPH case-cohort study)	Tulane	
La Réunion		ZikaRun: an integrative mother-infant inception cohort study to anticipate	Cellule Régionale de l'Institut de Veille Sanitaire océan Indien	INSERM

For peer review only - http://bmjopen.bmj.com/site/about/guidelines.xhtml

Country	City	Study Name	Coordinating Center(s)	Consortium Name*
		the introduction of Zika virus in the at-risk La Reunion island, Indian Ocean	2Département de Médecine Générale, UFR santé, Université de la Réunion, Saint Denis 3INSERM CIC1410, CHU Reunion, Saint Denis - Saint Pierre 4CH Gabriel Martin, Saint Paul 5Centre d'Etudes Périnatales de l'océan Indien (CEPOI), EA7388, Université de la Réunion, CHU Reunion, Saint Pierre 6UM 134 Processus Infectieux en Milieu Insulaire Tropical (PIMIT), Université de La Réunion, INSERM U1187, CNRS 9192, IRD 249 7UMR Diabète AthéroThRombose Océan Indien (DéTROI), INSERM U188, Sainte Clotilde, La Réunion"	
Jamaica, Haiti		ZIKAction: Mother to child transmission of Chikungunya, Dengue, and Zika Virus Infection: A prospective observational cohort study of pregnant women and their infants		ZIKAction

For peer review only - http://bmjopen.bmj.com/site/about/guidelines.xhtml

For peer review only

Country	City	Study Name	Coordinating Center(s)	Consortium Name*
Panama<br>El Salvador		Panama/El Salvador Influenza Birth Cohort Study with Added Zika Component		CDC
Spain		pedZIKARed/gestZIKARed Spanish Zika database for pregnant women and children	Barceola University Hospital Vall d'Hebron	ZIKAction
Suriname		A symptomatic cohort study in Zika infected pregnant women	Acadamic Hospital Paramaribo	
Western French Guiana		Association between Zika virus and foetopathy: a prospective cohort study in French Guiana	Centre Hospitalier de l'Ouest Guyanais Saint-Laurent du Maroni	

CDC=Centers for Disease Control and Prevention; IDAMS=International Research Consortium on Dengue Risk Assessment, Management, and Surveillance; INSERM=Institut National de la Santé Et de la Recherche Médicale; NIAID=National Institutes of Allergy and Infectious Disease; NIH=National Institutes of Health

For peer review only

Supplementary Table 2. PRISMA-P 2015 Checklist

This checklist has been adapted for use with protocol submissions to *Systematic Reviews* from Table 3 in Moher D et al: Preferred reporting items for systematic review and meta-analysis protocols (PRISMA-P) 2015 statement. *Systematic Reviews* 2015 **4**:1

Section/topic	#	Checklist item	Information reported		Page number(s)
			Yes	No	
**ADMINISTRATIVE INFORMATION**					
**Title**					
Identification	1a	Identify the report as a protocol of a systematic review	☒	☐	1
Update	1b	If the protocol is for an update of a previous systematic review, identify as such	☐	☒	
**Registration**	2	If registered, provide the name of the registry (e.g., PROSPERO) and registration number in the Abstract	☒	☐	1
**Authors**					
Contact	3a	Provide name, institutional affiliation, and e-mail address of all protocol authors; provide physical mailing address of corresponding author	☒	☐	1
Contributions	3b	Describe contributions of protocol authors and identify the guarantor of the review	☒	☐	26
**Amendments**	4	If the protocol represents an amendment of a previously completed or published protocol, identify as such and list changes; otherwise, state plan for documenting important protocol amendments	☐	☒	
**Support**					
Sources	5a	Indicate sources of financial or other support for the review	☒	☐	26
Sponsor	5b	Provide name for the review funder and/or sponsor	☒	☐	26
Role of sponsor/funder	5c	Describe roles of funder(s), sponsor(s), and/or institution(s), if any, in developing the protocol	☒	☐	26
**INTRODUCTION**					
**Rationale**	6	Describe the rationale for the review in the context of what is already known	☒	☐	9
**Objectives**	7	Provide an explicit statement of the question(s) the review will address with reference to participants, interventions, comparators, and outcomes (PICO)	☒	☐	12

For peer review only

BioMed Central The Open Access Publisher

For peer review only - http://bmjopen.bmj.com/site/about/guidelines.xhtml

Section/topic	#	Checklist item	Information reported Yes	Information reported No	Page number(s)
**METHODS**					
**Eligibility criteria**	8	Specify the study characteristics (e.g., PICO, study design, setting, time frame) and report characteristics (e.g., years considered, language, publication status) to be used as criteria for eligibility for the review	☒	☐	12
**Information sources**	9	Describe all intended information sources (e.g., electronic databases, contact with study authors, trial registers, or other grey literature sources) with planned dates of coverage	☒	☐	13
**Search strategy**	10	Present draft of search strategy to be used for at least one electronic database, including planned limits, such that it could be repeated	☒	☐	13
STUDY RECORDS					
Data management	11a	Describe the mechanism(s) that will be used to manage records and data throughout the review	☒	☐	14
Selection process	11b	State the process that will be used for selecting studies (e.g., two independent reviewers) through each phase of the review (i.e., screening, eligibility, and inclusion in meta-analysis)	☒	☐	13
Data collection process	11c	Describe planned method of extracting data from reports (e.g., piloting forms, done independently, in duplicate), any processes for obtaining and confirming data from investigators	☒	☐	13
**Data items**	12	List and define all variables for which data will be sought (e.g., PICO items, funding sources), any pre-planned data assumptions and simplifications	☒	☐	14
**Outcomes and prioritization**	13	List and define all outcomes for which data will be sought, including prioritization of main and additional outcomes, with rationale	☒	☐	14
**Risk of bias in individual studies**	14	Describe anticipated methods for assessing risk of bias of individual studies, including whether this will be done at the outcome or study level, or both; state how this information will be used in data synthesis	☒	☐	17
DATA					
**Synthesis**	15a	Describe criteria under which study data will be quantitatively synthesized	☒	☐	17
	15b	If data are appropriate for quantitative synthesis, describe planned summary measures, methods of handling data, and methods of combining data from studies, including any planned exploration of consistency (e.g., I^2, Kendall's tau)	☒	☐	18
	15c	Describe any proposed additional analyses (e.g., sensitivity or subgroup analyses, meta-regression)	☒	☐	17-22
	15d	If quantitative synthesis is not appropriate, describe the type of summary planned	☐	☒	

BioMed Central The Open Access Publisher

Section/topic	#	Checklist item	Information reported		Page number(s)
			Yes	No	
**Meta-bias(es)**	16	Specify any planned assessment of meta-bias(es) (e.g., publication bias across studies, selective reporting within studies)	☒	☐	22-24
**Confidence in cumulative evidence**	17	Describe how the strength of the body of evidence will be assessed (e.g., GRADE)	☒	☐	22

For peer review only

BioMed Central
The Open Access Publisher

Supplementary Table 3. Zika virus-related and general clinical trial databases (adapted from Reveiz, et al [1])

Data base name	Link
Clinical Trails.gov	https://clinicaltrials.gov/ct2/search
World Health Organization (WHO) International Clinical Trials Registry Platform (ICTRP)	http://apps.who.int/trialsearch/
United States Centers for Disease Control and Prevention (US-CDC)	https://www.cdc.gov/publications/
European Centers for Disease Control (E-CDC)	https://ecdc.europa.eu/en/publications-data
Pan American Health Organization (PAHO) Zika research portal	https://www.paho.org/zika-research/
Fiocruz Research portal	https://portal.fiocruz.br/
Sistema Nacional de Ética em Pesquisa (SISNEP)	http://portal2.saude.gov.br/sisnep/pesquisador/
Registro peruano de ensayos clínicos y de estudios observacionales (REPEC)	http://www.ensayosclinicos-repec.ins.gob.pe/acerca-del-repec/busqueda-de-ensayos-clinicos
Registro nacional de investigaciones en salud (ReNIS)	https://sisa.msal.gov.ar/sisa/#Renis
Registro nacional de ensayos clínicos (RNEC)	http://189.254.115.252/Resoluciones/Consultas/ConWebRegEnsayosClinicos.asp

Reference

1. Reveiz L, Haby MM, Martínez-Vega R, Pinzón-Flores CE, Elias V, Smith E, et al. Risk of bias and confounding of observational studies of Zika virus infection: A scoping review of research protocols. PLOS ONE. 2017;12(7):e0180220. doi: 10.1371/journal.pone.0180220.

For peer review only

Supplementary Text 1. ZIKV IPD-MA search strategy

PICO Question:

Population	Exposure	Comparator	Outcome (open)
Pregnant women and her fetus, infant, or child	ZIKV infection during pregnancy	No ZIKV infection during pregnancy	Primary: microcephaly, miscarriage, fetal loss. Secondary: early/late fetal death, ocular abnormalities, hearing loss, neuroimaging abnormalities, etc.

Medline (through Ovid):

1. exp Zika Virus Infection/ or exp ZIKA VIRUS/

2. (zika or ZIKV).ti,ab,kf.

3. 1 or 2

4. exp Pregnancy/ or exp Maternal Exposure/ or exp "Embryonic and Fetal Development"/ or exp "Congenital, Hereditary, and Neonatal Diseases and Abnormalities"/ or exp Infant/ or exp Child/

5. (pregnan* or matern* or gestation* or perinatal* or birth* or congenital* or newborn* or fetal or fetus* or foetal or foetus* or neonat* or infan* or toddler* or child*).ti,ab,kf.

6. 4 or 5

7. 3 and 6

8. 7 not (exp Animals/ not exp Humans/)

Embase (through Ovid):

1. exp Zika virus/ or exp Zika fever/

2. (zika or ZIKV).ti,ab,kw.

3. 1 or 2

4. exp pregnancy/ or exp pregnancy outcome/ or exp high risk pregnancy/ or exp pregnancy complication/ or exp maternal exposure/ or exp fetus/ or exp "functions of embryonic, fetal and placental structures"/ or exp Infant/ or exp infant disease/ or exp child/ or exp childhood disease/

5. (pregnan* or matern* or gestation* or perinatal* or birth* or congenital* or newborn* or fetal or fetus* or foetal or foetus* or neonat* or infan* or toddler* or child*).ti,ab,kw.

6. 4 or 5

7. 3 and 6

8. 7 not ((exp animal/ or exp nonhuman/) not exp human/)

For peer review only

BMJ Open

Understanding the relation between Zika virus infection during pregnancy and adverse fetal, infant, and child outcomes: a protocol for a systematic review and individual participant data meta-analysis of longitudinal studies of pregnant women and their infants and children

Journal:	*BMJ Open*
Manuscript ID	bmjopen-2018-026092.R1
Article Type:	Protocol
Date Submitted by the Author:	11-Feb-2019
Complete List of Authors:	Zika Virus Individual Participant Data Consortium, -<br>Wilder-Smith, Annelies; Nanyang Technological University, Lee Kong Chian School of Medicine<br>Wei, Yinghui; University of Plymouth, Centre for Mathematical Sciences<br>Velho Barreto de Araújo, Thalia; Universidade Federal de Pernambuco, Department of Social Medicine<br>VanKerkhove, Maria; Organisation mondiale de la Sante, Health Emergencies Programme<br>Turchi Martelli, Celina Maria; Oswaldo Cruz Foundation , Department of Collective Health, Institute Aggeu Magalhães (CPqAM)<br>Turchi, Marília Dalva; Federal University of Goias, Institute of Tropical Pathology and Public Health<br>Teixeira, Mauro; Universidade Federal de Minas Gerais<br>Tami, Adriana; University Medical Center Groningen, Department of Medical Microbiology<br>Souza, João ; University of São Paulo<br>Sousa, Patricia; State Department of Health of Maranhão, Reference Center for Neurodevelopment, Assistance, and Rehabilitation of Children<br>Soriano-Arandes, Antoni; University Hospital Vall d'Hebron, Department of Pediatrics<br>Soria-Segarra, Carmen; SOSECALI C. Ltda<br>Sanchez Clemente, Nuria; University of São Paulo, Department of Epidemiology<br>Rosenberger, Kerstin Daniela; UniversitatsKlinikum Heidelberg, Department of Infectious Diseases, Section Clinical Tropical Medicine<br>Reveiz, L; Pan American Health Organization, Evidence and Intelligence for Action in Health<br>Prata-Barbosa, Arnaldo; D'Or Institute for Research & Education, Department of Pediatrics<br>Pomar, Léo; Centre Hospitalier de l'Ouest Guyanais, Department of Obstetrics and Gynecology<br>Pelá Rosado, Luiza Emylce ; Goiânia State Health Secretary, Hospital Materno Infantil de Goiânia<br>Perez, Freddy; Pan American Health Organization, Communicable Diseases and Environmental Determinants of Health Department<br>Passos, Saulo; FMJ, Department of Pediatrics<br>Nogueira, Mauricio; Faculdade de Medicina de Sao Jose do Rio Preto,

Department of Dermatologic Diseases
Noel, Trevor P.; St. George’s University, Windward Islands Research and Education Foundation
Moura da Silva , Antônio ; Universidade Federal do Maranhão - São Luís , Department of Public Health
Moreira , Maria Elisabeth; Oswaldo Cruz Foundation (Fiocruz), Department of Neonatology
Morales, Ivonne; UniversitatsKlinikum Heidelberg, Department of Infectious Diseases, Section Clinical Tropical Medicine
Miranda Montoya, Maria Consuelo ; Universidad Industrial de Santander, Facultad de Salud
Miranda-Filho, Demócrito de Barros; University of Pernambuco, Faculty of Medical Sciences
Maxwell, Lauren; World Health Organization, Reproductive Health and Research; Emory University, Hubert Department of Global Health
Macpherson, Calum; St George's University, Windward Islands Research and Education Foundation
Low, Nicola; University of Bern, Bern, Switzerland, Institute of Social and Preventive Medicine
Lan , Zhiyi; McGill University, McGill University Health Centre
LaBeaud, Angelle Desiree ; Stanford Hospital, Pediatric Infectious Diseases
Koopmans, M; Erasmus Medical Center, Rotterdam, Netherlands, Department of Virology
Kim, Caron; World Health Organization, Department of Reproductive Health and Research
João, Esaú; Hospital Federal dos Servidores do Estado, Department of Infectious Diseases
Jaenisch, Thomas ; UniversitatsKlinikum Heidelberg, Department of Infectious Diseases, Section Clinical Tropical Medicine;
Hofer, C. B.; Universidade Federal do Rio de Janeiro, Instituto de Puericultura e Pediatria Martagão Gesteira
Gustafson, Paul; University of British Columbia, Statistics
Gérardin, Patrick; CHU La Réunion, INSERM CIC1410 Clinical Epidemiology; Universite de la Reunion, UM 134 PIMIT (CNRS 9192, INSERM U1187, IRD 249, Université de la Réunion)
Ganz, Jucelia S; Children’s Hospital Juvencio Matos
Fialho Dias, Ana Carolina; Universidade Federal de Minas Gerais
Elias, Vanessa; Pan American Health Organization, Sustainable Development and Environmental Health
Duarte, Geraldo; University of São Paulo, Department of Gynecology and Obstetrics
Debray, Thomas; University Medical Center Utrecht, Julius Center for Health Sciences and Primary Care
Cafferata, Maria Luisa; Instituto de Efectividad Clinica y Sanitaria, Mother and Children Health Research Department
buekens, pierre; Tulane University, School of Public Health and Tropical Medicine
Broutet, Nathalie; World Health Organization, Dept of Reproductive Health and Research
Brickley, Elizabeth B.; London School of Hygiene and Tropical Medicine, Department of Infectious Disease Epidemiology
Brasil, Patrícia ; Fundacao Oswaldo Cruz, Instituto de pesquisa Clínica Evandro Chagas
Brant, Fátima; Universidade Federal de Minas Gerais
Bethencourt, Sarah ; Universidad de Carabobo, Facultad de Ciencias de la Salud
Benedetti, Andrea; McGill University, Departments of Medicine and of Epidemiology, Biostatistics & Occupational Health
Avelino-Silva, Vivian ; Faculdade de Medicina da Universidade de Sao Paulo, Department of Infectious and Parasitic Diseases

	Arraes de Alencar Ximenes, Ricardo; Federal University of Pernambuco, Department of Tropical Medicine<br>Alves da Cunha, Antonio; Federal University of Rio de Janeiro, Department of Pediatrics<br>Alger, Jackeline; Universidad Nacional Autónoma de Honduras, Facultad de Ciencias Médicas
**Primary Subject Heading**:	Epidemiology
Secondary Subject Heading:	Global health, Infectious diseases, Research methods
Keywords:	individual participant data meta-analysisis, prognosis, congenital Zika syndrome, Zika virus, microcephaly, risk prediction model

SCHOLARONE™
Manuscripts

TITLE: Understanding the relation between Zika virus infection during pregnancy and adverse fetal, infant, and child outcomes: a protocol for a systematic review and individual participant data meta-analysis of longitudinal studies of pregnant women and their infants and children

Registration: PROSPERO international prospective register of systematic reviews record number (CRD42017068915)

Author:
Zika Virus Individual Participant Data Consortium

Individual authors listed in descending alphabetical order at the end of the publication:
Annelies Wilder-Smith
Lee Kong Chian School of Medicine
Nanyang Technological University
Singapore

Yinghui Wei
Centre for Mathematical Sciences
University of Plymouth
Plymouth, England

Thalia Velho Barreto de Araújo
Department of Social Medicine
Federal University of Pernambuco
Recife, Brazil

Maria VanKerkhove
Health Emergencies Programme
World Health Organization
Geneva, Switzerland

Celina Maria Turchi Martelli
Department of Collective Health
Institute Aggeu Magalhães (CPqAM)
Fundação Oswaldo Cruz (Fiocruz)
Recife, Brazil

Marília Dalva Turchi
Institute of Tropical Pathology and Public Health
Federal University of Goiás
Goiânia, Brazil

Mauro Teixeira
Instituto de Ciências Biológicas

For peer review only

For peer review only - http://bmjopen.bmj.com/site/about/guidelines.xhtml

Universidade Federal de Minas Gerais
Belo Horizonte, Brazil

Adriana Tami
Department of Medical Microbiology
University Medical Center Groningen
University of Groningen
Groningen, Netherlands

João Paulo Souza
Department of Social Medicine
University of São Paulo
São Paulo, Brazil

Patricia Sousa
Reference Center for Neurodevelopment, Assistance, and Rehabilitation of Children
State Department of Health of Maranhão
São Luís, Brazil

Antoni Soriano-Arandes
Department of Pediatrics
University Hospital Vall d'Hebron
Barcelona, Spain

Carmen Soria-Segarra
SOSECALI C. Ltda
Guayas, Ecuador

Nuria Sanchez Clemente
Department of Epidemiology
University of São Paulo
São Paulo, Brazil

Kerstin Daniela Rosenberger
Department for Infectious Diseases
Heidelberg University Hospital
Heidelberg, Germany

Ludovic Reveiz
Evidence and Intelligence for Action in Health
Pan American Health Organization
Washington, D.C., USA

Arnaldo Prata-Barbosa

For peer review only

For peer review only - http://bmjopen.bmj.com/site/about/guidelines.xhtml

Department of Pediatrics
D'Or Institute for Research & Education
Rio de Janeiro, Brazil

Léo Pomar
Department of Obstetrics and Gynecology
Centre Hospitalier de l'Ouest Guyanais
Saint-Laurent du Maroni, French Guiana

Luiza Emylce Pelá Rosado
Hospital Materno Infantil de Goiânia
Goiânia State Health Secretary
Goiás, Brazil

Freddy Perez
Communicable Diseases and Environmental Determinants of Health Department
Pan American Health Organization
Washington, D.C., USA

Saulo Passos
Department of Pediatrics
Faculty of Medicine of Jundiai
São Paulo, Brazil

Mauricio Nogueira
Department of Dermatologic Diseases
Faculdade de Medicina de São José do Rio Preto
São José do Rio Preto, Brazil

Trevor P. Noel
Windward Islands Research and Education Foundation
St. George's University
True Blue Point, Grenada

Antônio Moura da Silva
Department of Public Health
Federal University of Maranhão
São Luís, Brazil

Maria Elisabeth Moreira
Department of Neonatology
Fundação Oswaldo Cruz (Fiocruz)
Rio de Janeiro, Brazil

Ivonne Morales

For peer review only

For peer review only - http://bmjopen.bmj.com/site/about/guidelines.xhtml

Department for Infectious Diseases
Heidelberg University Hospital
Heidelberg, Germany

María Consuelo Miranda Montoya
Facultad de Salud
Universidad Industrial de Santander
Santander, Colombia

Demócrito de Barros Miranda-Filho
Faculty of Medical Sciences
University of Pernambuco
Recife, Brazil

*corresponding author
Lauren Maxwell
Department of Reproductive Health and Research
World Health Organization
Geneva, Switzerland
maxwelll@who.int
404.728.2017

Calum N.L. Macpherson
Windward Islands Research and Education Foundation
St. George's University
True Blue Point, Grenada

Nicola Low
Institute of Social and Preventive Medicine
University of Bern
Bern, Switzerland

Zhiyi Lan
McGill University Health Centre
McGill University
Montréal, Canada

Angelle Desiree LaBeaud
Pediatric Infectious Diseases
Stanford Hospital
California, USA

Marion Koopmans
Department of Virology
Erasmus MC

For peer review only

Rotterdam, Netherlands

Caron Kim
Department of Reproductive Health and Research
World Health Organization
Geneva, Switzerland

Esaú João
Department of Infectious Diseases
Hospital Federal dos Servidores do Estado
Rio de Janeiro, Brazil

Thomas Jaenisch
Department for Infectious Diseases
Heidelberg University Hospital
Heidelberg, Germany

Cristina Hofer
Instituto de Puericultura e Pediatria Martagão Gesteira
Universidade Federal do Rio de Janeiro
Rio de Janeiro, Brazil

Paul Gustafson
Department of Statistics
University of British Columbia
Vancouver, Canada

Patrick Gérardin
Centre for Clinical Investigation (CIC1410)
Centre Hospitalier Universitaire de La Réunion
Saint Pierre, Réunion, France

Jucelia S. Ganz
Children's Hospital Juvencio Matos
São Luís, Brazil

Ana Carolina Fialho Dias
Instituto de Ciências Biológicas
Universidade Federal de Minas Gerais
Belo Horizonte, Brazil

Vanessa Elias
Sustainable Development and Environmental Health
Pan American Health Organization
Washington, D.C., USA

For peer review only - http://bmjopen.bmj.com/site/about/guidelines.xhtml

Geraldo Duarte
Universidade de São Paulo
Ribeirão Preto, Brazil

Thomas Paul Alfons Debray
Department of Epidemiology
University Medical Center Utrecht
Utrecht, Netherlands

María Luisa Cafferata
Mother and Children Health Research Department
Institute for Clinical Effectiveness and Health Policy
Buenos Aires, Argentina

Pierre Buekens
School of Public Health and Tropical Medicine
Tulane University
Louisiana, USA

Nathalie Broutet
Department of Reproductive Health and Research
World Health Organization
Geneva, Switzerland

Elizabeth B. Brickley
Department of Infectious Disease Epidemiology
London School of Hygiene and Tropical Medicine
London, England

Patrícia Brasil
Fundação Oswaldo Cruz
Instituto de pesquisa Clínica Evandro Chagas
Rio de Janeiro, Brazil

Fátima Brant
Instituto de Ciências Biológicas
Universidade Federal de Minas Gerais
Belo Horizonte, Brazil

Sarah Bethencourt
Facultad de Ciencias de la Salud
Universidad de Carabobo
Valencia, Venezuela

For peer review only - http://bmjopen.bmj.com/site/about/guidelines.xhtml

Andrea Benedetti
McGill University Health Centre
McGill University
Montréal, Canada

Vivian I. Avelino-Silva
Departamento de Moléstias Infecciosas e Parasitárias
University of São Paul
São Paulo, Brazil

Ricardo Arraes de Alencar Ximenes
Department of Tropical Medicine
Federal University of Pernambuco
Recife, Brazil

Antonio Alves da Cunha
Department of Pediatrics
Federal University of Rio de Janeiro
Rio de Janeiro, Brazil

Jackeline Alger
Facultad de Ciencias Médicas
Universidad Nacional Autónoma de Honduras
Tegucigalpa, Honduras

Word Count: 7900

Abstract

Introduction

Zika virus (ZIKV) infection during pregnancy is a known cause of microcephaly and other congenital and developmental anomalies. In the absence of a ZIKV vaccine or prophylactics, principal investigators (PIs) and international leaders in ZIKV research have formed the ZIKV Individual Participant Data (IPD) Consortium to identify, collect, and synthesize IPD from longitudinal studies of pregnant women that measure ZIKV infection during pregnancy and fetal, infant, or child outcomes.

Methods and analysis

We will identify eligible studies through the ZIKV IPD Consortium membership and a systematic review and invite study PIs to participate in the IPD-MA. We will use the combined dataset to estimate the relative and absolute risk of congenital Zika syndrome (CZS), including microcephaly and late symptomatic congenital infections; identify and explore sources of heterogeneity in those estimates; and develop and validate a risk prediction model to identify the pregnancies at the highest risk of CZS or adverse developmental outcomes. The variable accuracy of diagnostic assays and differences in exposure and outcome definitions means that included studies will have a higher level of systematic

For peer review only - http://bmjopen.bmj.com/site/about/guidelines.xhtml

variability, a component of measurement error, than an IPD-MA of studies of an established pathogen. We will use expert testimony, existing internal and external diagnostic accuracy validation studies, and laboratory external quality assessments to inform the distribution of measurement error in our models. We will apply both Bayesian and frequentist methods to directly account for these and other sources of uncertainty.

Ethics and dissemination
The IPD-MA was deemed exempt from ethical review. We will convene a group of patient advocates to evaluate the ethical implications and utility of the risk stratification tool. Findings from these analyses will be shared via national and international conferences and through publication in open access, peer-reviewed journals.

Registration: PROSPERO International prospective register of systematic reviews (CRD42017068915)

Keywords: individual participant data meta-analysis, risk prediction model, Zika virus, microcephaly, congenital Zika syndrome, prognosis, Bayesian methods, data sharing

Strengths and limitations of this study

- This is one of the first applications of an IPD-MA to address public health concerns in the context of an emerging pathogen. Lessons learned from this IPD-MA may facilitate the formation of research collaborations to inform the public health response to future epidemics.
- By using a diversity of populations to develop and validate the risk prediction tool that identifies pregnancies at the highest risk of CZS, the IPD-MA provides a real opportunity to help inform how clinicians and laboratory scientists communicate ZIKV results to pregnant women and their families.
- There is a high degree of variability in the accuracy of diagnostic assays for ZIKV, co-infection, and outcome ascertainment. Addressing this variability will be a challenge and ultimately a limitation of the accuracy of IPD-MA results.
- There is no gold standard diagnostic assay to detect ZIKV infection during pregnancy and few studies have been able to measure fetal infection. The statistical methods traditionally used to account for measurement error in IPD-MA need to be adapted to account for the myriad, correlated sources of uncertainty that arise in the synthesis of participant-level data from studies that arise in the context of an emerging pathogen.

INTRODUCTION

Zika virus (ZIKV) infection during pregnancy is an acknowledged cause of microcephaly and other forms of fetal brain defects and disability.[1 2] ZIKV is an arbovirus in the genus Flavivirus that is usually transmitted through the female *Aedes aegypti* mosquito. *Aedes aegypti* is also the main vector for dengue (DENV), urban yellow fever (YF), and chikungunya viruses. The Asian strain of ZIKV has been

For peer review only - http://bmjopen.bmj.com/site/about/guidelines.xhtml

shown to replicate in the placenta and fetal brain;[3] ZIKV transmitted from mother to fetus during pregnancy may have a detrimental effect on fetal brain development.[4-6] Microcephaly, generally defined as a 2-3 standard deviation reduction from the mean head circumference,[7 8] is caused by infections during pregnancy, maternal diet, drug abuse, genetic factors, or environmental exposures.[9 10] Microcephaly (congenital or acquired) may be associated with developmental delays; intellectual, hearing, and visual impairment; and epilepsy.[11] The causal relation between ZIKV and a spectrum of fetal anomalies that includes microcephaly, now known as congenital Zika syndrome (CZS),[12] has been supported through several case-control;[13 14] cohort;[15 16] and surveillance studies;[17] animal and cell studies;[18] and through two systematic reviews of the evidence for causality that considered all study designs.[1 2] The relation between ZIKV infection during pregnancy and miscarriage (pregnancy loss <20 weeks gestation) and fetal loss (pregnancy loss ≥20 weeks gestation) is still under investigation.

Prior to the 2013-16 epidemic waves, ZIKV infection was known clinically as a mild illness characterized by symptoms shared with other arboviruses, including: maculopapular rash; headache; fever; non-purulent conjunctivitis; and/or joint and muscle pain.[19] During the 2015-16 ZIKV outbreak in Brazil, which extended to a number of other Latin American countries, there was a sharp increase in reports of microcephaly and other neonatal neurological conditions and in Guillain-Barré syndrome (GBS),[20-22] an autoimmune neurologic disorder. Subsequent analysis of medical records collected during and after the 2013-2014 ZIKV outbreak in French Polynesia identified several ZIKV-linked pregnancies that had not been recorded earlier because they ended in elective abortion or stillbirth. The re-analysis of medical records indicated that the prevalence of both microcephaly and GBS had increased in the wake of the outbreak in French Polynesia.[23 24] The Pan American Health Organization (PAHO) issued a ZIKV Epidemiological Alert for Member States on May 7, 2015,[25] the Brazilian Ministry of Health (MOH) declared a national public health emergency due to the time and cluster of microcephaly cases identified in Northeastern Brazil on November 12, 2015,[26] and the World Health Organization (WHO) declared that the clusters of microcephaly and related neurological complications represented a Public Health Emergency of International Concern on February 1, 2016.[27]

Zika virus presents myriad challenges from an epidemiological, virological, diagnostic, and outbreak control perspective. Diagnosing ZIKV infection is complicated by the absence of symptoms in most cases or the presence of non-specific symptoms; cross-reactivity with DENV;[28 29] the short window for diagnosing acute infection; and the lack of point-of-care diagnostics.[30] Recent research suggests that the relation between ZIKV infection during pregnancy and fetopathology may vary by virus genotype or lineage; primary versus secondary infection;[31] and DENV-immune status and genotype in the presence of coinfection[29 32 33] The unequal spatial distribution of microcephaly cases has been discussed extensively.[34-36] These differences may be related to population-level differences in baseline risk of adverse fetal outcomes (clinically important heterogeneity), differences in study design (e.g. inclusion criteria; measurement of important co-factors), or to measurement error, defined as the difference between the observed and actual level of a given variable. Laboratory confirmation of ZIKV infection and co-infection differs by diagnostic algorithms (e.g. definition of positive and negative ZIKV diagnostic assay results); factors that affect the regularity of testing (e.g. provision of incentives, distance from testing center, differences across protocols); population-specific distribution of related co-infections;

For peer review only - http://bmjopen.bmj.com/site/about/guidelines.xhtml

differing levels of training of laboratory staff; and the accessibility of materials and technology (e.g., ultrasound, immunoassays, reliability panels), among other factors. In addition to documented difficulties in accurately measuring infant head circumference, measurement standards for identifying microcephaly differ across populations and standards themselves may not appropriately classify reduced or enlarged head circumference.[37 38]

Our limited understanding of the absolute risk of adverse fetal, infant, and child outcomes in ZIKV-infected mothers led to calls from several governments suggesting that women avoid becoming pregnant for as long as two years.[39 40] ZIKV disproportionately affects low-income populations residing in areas with poor living conditions.[41] The impetus placed on women to delay pregnancy as a ZIKV control measure is complicated by the limited access to contraception and safe abortion in many of the countries and regions with the highest burden of ZIKV-related microcephaly.[42 43] Identifying the risk factors for CZS is a global health priority and central for prioritizing resource allocation for vector control and effective and targeted family planning interventions, and for improving risk counseling for ZIKV-infected pregnant women or women planning a pregnancy in endemic areas.

Rationale for the individual participant data meta-analysis of longitudinal studies of pregnant women

Individual participant data meta-analysis (IPD-MA) is the quantitative synthesis of participant-level data from included studies, while appropriately accounting for the clustering of information at the study level. The proposed IPD-MA will combine de-identified, participant-level cohort data from different populations of pregnant women to identify and quantify the relative importance of different predictors of CZS. Individual participant data (IPD) have a number of analytic benefits over aggregate data meta-analysis (AD-MA), a form of knowledge synthesis that combines study-level measures of effect.[44 45] Individual participant data facilitates the assessment of effect measure modification, the development and validation of risk prediction models, and the application of a unified analytic approach. In addition to using the same statistical model across studies, with IPD we can apply the same or similar exclusion criteria, diagnostic algorithms, methods for addressing missing data and confounding, and conduct the same types of sensitivity analyses needed to explore unexplained within- and between-study heterogeneity.

Increased precision of estimates

Timely, accurate, and reliable predictions are predicated on well-designed studies that minimize the risk of bias, adequate sample size, and the inclusion of a diversity of populations. Adequate sample size is crucial for precise estimation of the risk of CZS within important subgroups (e.g. women infected during the first trimester; pregnant women with previous or concurrent DENV, CHIKV, and STORCH pathogen exposure). Vector control measures, including pesticides, public education campaigns, the use of drones to detect standing water, and the introduction of sterilized male vectors to reduce *Aedes aegypti* populations, have been implemented in the wake of the 2015/2016 ZIKV epidemics.[46-48] Fortunately, these measures, in combination with other factors that are currently being investigated, seem to have

For peer review only - http://bmjopen.bmj.com/site/about/guidelines.xhtml

reduced the numbers of ZIKV infections during the 2017/2018 epidemic cycle. While many studies have followed infants to the end of their first year, certain developmental milestones can only be assessed after age two[49] or when a child reaches school age. Leveraging limited data from studies with extended follow-up of ZIKV-infected and non-infected women will be essential for estimating the risk of more subtle, long-term effects of ZIKV infection during pregnancy. By combining data from individual studies, the proposed IPD-MA will improve the precision of risk estimates.

Identify and quantify the relative importance of effect measure modifiers

The benefits of using IPD rather than AD to assess effect measure modification and interaction are myriad.[50] In a one-stage analysis with IPD, subject level data are meta-analyzed using the exact binomial distribution; in a two-stage analysis of IPD or AD, study-level outcome measures are combined assuming asymptomatic normality.[51] In a one-stage analysis of IPD, study- and individual-level sources of heterogeneity can be assessed concurrently and IPD are better able to identify heterogeneity in the context of rare events or small studies.[50 52] Individual studies are often powered to detect the overall effect of the exposure rather than subgroup effects. Due to variations in the characteristics of the affected populations and in the potential confounders and effect modifiers measured by different studies, it is unlikely that individual studies will be powered to definitively quantify the importance of different sources of heterogeneity in the relation between ZIKV infection during pregnancy and adverse fetal, infant, or child outcomes.

Clinical risk prediction to inform decision-making and resource allocation

While there are a number of vaccine trials underway,[53] the development of a ZIKV vaccine is complicated by the necessity of testing the vaccine in pregnant women; assessing whether the vaccine is associated with development of GBS; the difficulties inherent in developing an arbovirus vaccine;[46 54-56] findings from *in vivo* studies that indicate cross-reactivity between ZIKV and DENV or West Nile virus is related to antibody-dependent enhancement of ZIKV infection;[55 57 58] and by the potential use of prevention of infection as a vaccine efficacy endpoint.[59] In this context, identifying the pregnancies at the highest risk of adverse neonatal and later developmental outcomes is critical for effective resource allocation and prevention strategies. We will use participant-level data to develop and externally validate clinical risk prediction models to facilitate the identification of pregnancies that are most likely to result in ZIKV-related adverse fetal or infant outcomes and longer-term developmental delays.

Standardization and cross-national partnerships to inform the public health response to emerging pathogens

Formation of the ZIKV IPD Consortium

The ZIKV IPD Consortium is a global collaboration designed to streamline the international response to ZIKV. To facilitate cross-country analyses and a coordinated response to ZIKV, representatives from WHO, PAHO, the US Centers for Disease Control and Prevention (CDC), the National Institutes of Health (NIH), the National Institute of Allergy and Infectious Diseases (NIAID), Institut national de la santé et de

For peer review only - http://bmjopen.bmj.com/site/about/guidelines.xhtml

la recherche médicale (INSERM), Institut Pasteur, and the networks of Fundação Oswaldo Cruz (Fiocruz), Grupo de Pesquisa da Epidemia da Microcefalia (MERG)/ZikaPlan, ZIKAlliance, ZIKAction, the Consortium for the Standardization of Influenza Seroepidemiology (CONSISE), and International Severe Acute Respiratory and Emerging Infection Consortium (ISARIC) have developed a standardized protocol for cohorts of pregnant women and their infants exposed to ZIKV to facilitate the proposed IPD-MA; identified existing or planned cohorts; and prospectively introduced cohort principal investigators (PI)s and MOH officials to the methodological and public health benefits related to IPD-MA in the context of Zika. Many of the longitudinal studies and surveillance systems identified to date through the review of country-level registries, existing literature reviews, and ZIKV IPD Consortium membership have agreed to contribute de-identified, participant level data to the analysis. A complete list of the studies and surveillance systems who have agreed to contribute data to the ZIKV IPD Consortium led IPD-MA is included in **Supplementary Table 1**.

Standardized protocols for cohorts of pregnant women and their infants

A multiplicity of mechanisms for exposure and outcome ascertainment as well as differences in the measurement of important cofactors are known challenges for the meta-analysis of data from individual research studies. To minimize the potential for heterogeneity caused by differences in study inclusion criteria and the measurement of ZIKV, infant outcomes, and important cofactors, WHO/PAHO, Institut Pasteur, Fiocruz, CONSISE, and ISARIC convened an international meeting of ZIKV researchers and MOH officials in June of 2016 to develop standardized protocols and data collection instruments for cohort studies of pregnant women and newborns and other ZIKV-related studies.[60] Standardization of protocols and data collection instruments was intended to minimize differences in case ascertainment and data collection methods to facilitate data synthesis and the identification of sources of heterogeneity in the relation between congenital Zika infection and adverse fetal, infant, and child outcomes. The protocols were made available on WHO website in October 2016 (http://www.who.int/reproductivehealth/zika/en). The standardized protocols do not include detailed guidance on laboratory methods, but testing algorithms were developed by an expert panel and made available on the WHO website in March 2016 (http://www.who.int/csr/resources/publications/zika/laboratory-testing/en/). The IPD-MA will need to account for the between- and within-study differences in diagnostic assays and testing algorithms.

OBJECTIVES OF THE IPD-MA

1. Estimate the absolute and relative risks of fetal infection; miscarriage (<20 weeks gestation), fetal loss (≥ 20 weeks gestation), microcephaly, and other manifestations of CZS and later developmental delays for women who do and do not experience ZIKV infection during pregnancy.

2. Identify factors that modify women's risk of adverse ZIKV-related fetal, infant, and child outcomes and infants' risk of infection (e.g. gestational age at time of infection, clinical or

For peer review only - http://bmjopen.bmj.com/site/about/guidelines.xhtml

subclinical illness, concurrent or prior arbovirus exposure, other congenital infections, and other posited effect measure modifiers).

3. Use information on the relative importance of different effect measure modifiers identified in Objective 2 to decompose the total effect of ZIKV infection during pregnancy on adverse fetal, infant, and child outcomes into 1) the direct effect of ZIKV; 2) the indirect effect of ZIKV as mediated by the effect measure modifier of interest (e.g. DENV, CHIKV, or STORCH pathogens); and 3) the effect of the interaction between ZIKV and the mediator of interest.

4. Develop and validate a risk prediction tool to identify pregnant women at a high risk of an adverse ZIKV-related outcome and to inform couples planning a pregnancy, healthcare providers, and/or resource mobilization (e.g. vector control strategies; antenatal care; open access to contraception).

METHODS & ANALYSIS

This protocol has been drafted in accordance with the PRISMA-P Statement (**Supplementary Table 2**).[61] The proposed systematic review and meta-analysis will follow the PRISMA-IPD guidelines for the systematic review of non-randomized studies.[62]

Step 1. Study identification

Eligibility criteria

Eligible studies will use a longitudinal design where ZIKV infection is measured in pregnant women prior to outcome ascertainment. Eligible studies may include cohort studies, case-cohort studies, randomized control trials, or active surveillance systems, regardless of publication status. Studies may enroll symptomatic and/or asymptomatic women prior to or following a confirmed pregnancy. Included studies and active surveillance systems will test women for ZIKV infection during pregnancy, follow women until the end of pregnancy, and assess for CZS or related fetal, infant, or child outcomes (see Table 1). We will exclude studies with fewer than 10 participants and limit included surveillance systems to those that capture country or territory-level active surveillance data (i.e. individual hospital active surveillance data will not be included). Before sharing participant-level data, research studies will be asked to provide documentation of ethics review.

Information sources

1. ZIKV IPD Consortium

We anticipate that most eligible studies will have been identified through the efforts of the ZIKV IPD Consortium. The Consortium is an international initiative that is meant to include the PIs from all planned, ongoing, or completed ZIKV longitudinal studies at the time of this review. We have searched clinical trials and ZIKV-related databases[63] (**Supplementary Table 3**) to identify existing or planned

For peer review only - http://bmjopen.bmj.com/site/about/guidelines.xhtml

longitudinal studies. We have circulated the list of ongoing or planned ZIKV-related longitudinal studies of pregnant women to MOH Officials in countries with autochthone ZIKV transmission and to PIs of ZIKV cohorts and asked them to update the list as necessary.

2. Systematic review

We will perform a systematic search of biomedical databases for published longitudinal studies and protocols. The search strategy is based on Medical Subject Headings (MeSH) and text-based search terms for ZIKV, pregnant women, infants, and children. The search strategy was developed in collaboration with an information scientist and adapted for the following electronic databases: Embase(Medline), Embase(Ovid), and SCOPUS (see **Supplementary Text 1** for the search strategy for Embase (Medline and Ovid). We also will search the additional databases listed in **Supplementary Table 3** and review the reference lists of published systematic reviews and the list of studies produced by a living systematic review of ZIKV studies conducted by the University of Bern[64] to identify additional studies. After removing duplicates from the list of identified studies, two reviewers will independently screen the title and abstracts of included studies to identify longitudinal studies or active surveillance systems that measure ZIKV infection during pregnancy and subsequent fetal, infant, or child outcomes. Disagreements about study inclusion will be resolved by consensus.

Collection of study-level data

We will contact the PIs of eligible studies identified through either the ZIKV IPD Consortium or the electronic searches to invite them to take part in the IPD-MA and ask them to provide a copy of their study protocol. We will develop and pilot an electronic data extraction form to record study-level characteristics for all eligible studies, regardless of whether study PIs agree to participate in the IPD-MA. Two reviewers will independently review protocols and study-related publications to extract data on study design; study population; enrollment, follow-up and laboratory procedures; assay and specimen type; criteria used to define ZIKV infection and timing of infection; and exposure, cofactor and outcome ascertainment for all eligible studies. We will ask study PIs for clarification if there are outstanding questions or disagreements regarding study-level data.

Step 2. Collection, review, and synthesis of de-identified, participant-level data

We will contact the PIs and authors of studies that meet our inclusion criteria to request de-identified, participant-level data on select variables and the associated surveys and data dictionaries or codebooks. If study data have been imputed, we will request both the original and imputed data so that we can apply consistent imputation methods across studies and review the imputed dataset for validation purposes. To reduce the burden on individual studies and ensure clear documentation of all steps in the creation of the synthesized dataset, we will use the study codebooks or data dictionaries to develop study-specific code in the statistical language used by the study data manager that selects only the study variables required for the proposed analyses and removes information that could be used to identify individual participants. The study's data manager will apply the code to the original dataset. The de-identified, participant-level data will be transferred from the study site to Emory University, which will serve as the WHO data synthesis partner center, using secure file transfer protocol and will be protected

For peer review only - http://bmjopen.bmj.com/site/about/guidelines.xhtml

on a secure server with standard encryption and by the Emory University firewall. Data synthesis-related decisions will be reviewed by a ZIKV IPD Consortium membership and will be recorded using Jupyter Notebook.[65] Researchers that are unable or unwilling to provide their participant data after at least four attempts at contact by the project team over a period of six months will be excluded from the IPD-MA and we will report the reason for their exclusion. When IPD are not available for a given study, we will extract study-level effect estimates from any publications to compare study-level estimates from all eligible studies, whether or not they provide data for the IPD-MA.

Variables of interest

Despite efforts to develop protocols that can be applied across studies, there will be significant cross-study heterogeneity in how congenital Zika infection, cofactors, and outcomes are measured and reported. Exposure, outcome variables, and posited confounders and effect measure modifiers are listed in Table 1. Where possible, ZIKV and other infections (e.g. DENV, CHIKV, STORCH pathogens) will be modelled as time-varying, rather than time-fixed covariates. Given that the case definitions for microcephaly have changed over time (and may change during the course of included studies), we will allow for the coding of variables with different definitions (i.e. WHO fetal growth chart,[66] Fenton scale[67], INTERGROWTH 21st Project[49]). We will ask studies for data on the continuous measures used to make diagnoses (e.g. viral load; head circumference) rather than just the diagnoses themselves (e.g. maternal ZIKV infection, microcephaly). Using continuous variables will allow us to test the sensitivity of results to the application of different cutoffs and the reference standards used to generate Z-scores. Definitions for miscarriage, fetal loss, and other pregnancy outcomes vary across countries. We will explore the sensitivity of project findings to different outcome definitions.

Table 1. Participant-level variables of interest

Exposure	Maternal ZIKV infection (diagnosis: confirmed, probable, unlikely; primary, secondary, naïve; viral load)
	Fetal or placental ZIKV infection (diagnosis: confirmed, probable, unlikely; primary, secondary, naïve; viral load)*
Primary outcomes	Miscarriage (<20 weeks gestation)
	Fetal loss (≥20 weeks gestation)
	Microcephaly (diagnosis: severe microcephaly, microcephaly, normocephaly, macrocephaly; Z-score)
	CZS (diagnosis: confirmed, probable, unlikely)
Secondary fetal outcomes†	Induced abortion with microcephaly (diagnosis: confirmed, probable, unlikely)
	Early fetal death (20-27 weeks gestation)
	Late fetal death (≥28 weeks gestation)
	Late fetal death (≥28 weeks gestation) with microcephaly
	Placental insufficiency (diagnosis: confirmed, probable, unlikely)‡
	Intrauterine growth restriction
Secondary infant outcomes†	Postnatal microcephaly (diagnosis: severe microcephaly, microcephaly, normocephaly, macrocephaly; Z-score)

For peer review only - http://bmjopen.bmj.com/site/about/guidelines.xhtml

	Gestational age at birth
	Birth weight (diagnosis: normal birth weight; low birth weight; very low birth weight; extremely low birth weight; Z-score)
	Craniofacial disproportion
	Neuroimaging abnormalities (intracranial calcification, lissencephaly, hydranencephaly, porencephaly, ventriculomegaly, posterior fossa abnormalities, cerebellar hypoplasia, corpus callosal and vermian dysgenesis; focal cortical dysplasia)
	Postnatal intraventricular hemorrhage
	Motor abnormalities (hypotonia, hypertonia, hyperreflexia, spasticity, clonus, extrapyramidal symptoms)§
	Seizures, epilepsy§
	Ocular abnormalities (blindness, other)§
	Congenital deafness or hearing loss§
	Congenital contractures (arthrogryposis, uni or bilateral clubfoot)
	Other non-neurologic congenital abnormalities
Secondary outcomes detected after the infant period**	Cortical auditory processing
	Neurodevelopment (expressive and receptive language, fine and gross motor skills, attention and executive function, memory and learning, socioemotional development, overall neurodevelopmental score)
	Vision (Cardiff test)
Posited confounders	Demographic factors (age, education, marital status, racial/ethnic group; BMI)
	Socioeconomic factors
	Maternal smoking, illicit drug and alcohol use
	Maternal prescription drug use, vaccination
	Maternal experience of violence during pregnancy; infant or child exposure to intimate partner violence[68]
	Workplace or environmental exposures to teratogenic substances (e.g. maternal exposure to lead, mercury)
Potential effect measure modifiers	Genetic anomalies, metabolic disorders, perinatal brain injury
	Gestational age, term at birth
	Timing of infection during pregnancy
	Clinical/subclinical illness
	Viral genotype and load
	Concurrent or prior flavi- or alphavirus infection
	Maternal history of YF or JE vaccination

For peer review only - http://bmjopen.bmj.com/site/about/guidelines.xhtml

	Maternal immunosuppressive conditions, disorders, comorbidities (e.g. chronic hypertension, diabetes), or pregnancy-related conditions (e.g. pre-eclampsia, gestational diabetes)
	Intrauterine exposure to STORCH pathogens
	Maternal malnutrition
	Presence and severity of maternal and infant clinical symptoms

CZS=congenital Zika syndrome, JE=Japanese encephalitis; STORCH=syphilis, toxoplasmosis, rubella, cytomegalovirus, and herpes; YF=yellow fever virus; ZIKV=Zika virus
*Fetal ZIKV infection will be considered as both an exposure and an outcome; definition of fetal infection will be based on clinical and radiological criteria defined by an expert panel
†Both with and without microcephaly
‡As estimated by antenatal consequences of placental insufficiency, including fetal growth restriction, oligohydramnios, non-reassuring fetal heart rate tracing or small for gestational age at birth as markers of placental insufficiency.
§May also be detected after the infant period
** As measured by the Bayley Scale;[69] Ages and Stages;[70] INTERGROWTH-21st Neurodevelopmental Assessment[49]

Assessing the integrity of de-identified, participant-level data

We will review the distribution of variables to identify potential outliers and to assess the proportion missing within each study. We will discuss the distribution of key variables with the study data manager to identify and address any inconsistencies. If there has been a publication related to a given longitudinal study, we will attempt to replicate the Table 1 presented in the publication and will resolve any inconsistencies with the data manager.

Synthesis of participant-level data

Given that these longitudinal studies and active surveillance systems are part of the global research response to an emerging pathogen, there is a high degree of variability in the data that have been collected across studies and the algorithms that have been applied to define ZIKV exposure, symptoms, components of CZS, etc. Where possible, we will ask studies for the individual factors (i.e. fever, rash) that were used to define certain parameters (i.e. clinical infection) to ensure cross-study consistency in composite markers. Similarly, we will combine the data inputs for exposure, cofactor, and outcome classification algorithms to reduce cross-study differences in the classification of important factors.

Critical review of study quality

We will use the Cochrane Methodological Quality Assessment of Observational Studies[71] and the Q-Coh tool[72] to help describe the risk of bias within non-randomized studies and will apply the Cochrane Risk of Bias 2.0 tool to assess the risk of bias in randomized controlled trails.[73] Rather than using a score-based bias assessment, a panel that includes experts on the evaluation of laboratory assays and external quality assessment (EQA); obstetrics; and perinatal epidemiology will provide a detailed description of the role of selection, confounding, and measurement-related biases within studies.

Step 3. Statistical analyses

For peer review only - http://bmjopen.bmj.com/site/about/guidelines.xhtml

***Objectives 1 & 2**. Estimate the absolute and relative risks of adverse ZIKV-related fetal, infant, and child outcomes; identify and quantify relative importance of sources of heterogeneity*

Estimating the absolute risk of CZS by the gestational age of the fetus at the time of infection is as important as it is difficult. Early in the outbreak, cohort studies limited enrollment to symptomatic pregnant women. While an estimated 50-70% of infections are subclinical, when symptoms are detected they generally appear 3-14 days after infection.[74] For asymptomatic infections, the gestational age of infection is interval censored because it is defined by the last negative and first positive tests for ZIKV. Rather than using the midpoint between the last negative and first positive ZIKV test, which is known to be biased, we will impute the trimester or week that asymptomatic infections occurred using methods that are routinely applied in studies with interval censored covariates in the field of perinatal research.[75] [76] In Table 2, we present sample definitions for the absolute risk of fetal and infant outcomes. These definitions will be reviewed prior to analysis and publication and we will assess the sensitivity of our results to the definition applied. Later developmental outcomes (e.g. neurodevelopment, cortical auditory processing), listed in Table 1 as secondary outcomes, will follow a fetuses-at-risk approach.[77] We will apply censoring to account for competing risks where necessary.

Table 2. Definitions applied to estimation of absolute risk of primary fetal and infant outcomes

Outcome	Numerator	Denominator
Miscarriage	number of miscarriages (pregnancy loss prior to 20 weeks gestation)	total number of pregnancies
Early fetal death	number of pregnancies lost between 20-27 weeks gestation	total number of pregnancies carried to 20 weeks gestation
Late fetal death	number or pregnancies lost at or following 28 weeks gestation	total number of pregnancies carried to 28 weeks gestation
Microcephaly	number of microcephaly cases	total number of pregnancies carried to ≥24 weeks gestation, when microcephaly can be assessed by ultrasound in ZIKV-infected mothers,[38] we will consider all pregnancies regardless of whether the pregnancy results in a live birth.

We will apply mixed binomial models for binary outcomes, and multinomial models for categorical outcomes, with a logit link to provide estimates for each measure of absolute risk by week or trimester of congenital infection. Because of the differences in baseline risks across populations, pooling measures of absolute risk across studies may not be clinically relevant and can even be misleading.[78] We will combine study-level estimates of absolute risk through: 1) a one-stage meta-analysis (mixed binomial or multinomial model with a log link) that includes study-level sources of heterogeneity and a separate

For peer review only - http://bmjopen.bmj.com/site/about/guidelines.xhtml

intercept for each study to account for additional cross-study differences in baseline risk; and 2) a forest plot of study-level estimates of absolute risk that does not include a summary meta-analytic estimate.

Absolute measures of effect are considered more important for informing clinical practice than relative measures.[79] We will conduct both 1) a one-stage meta-analysis where we estimate the relative risk of the aforementioned outcomes of interest by congenital Zika infection across studies and 2) a two-stage meta-analysis where we estimate the relative risk in each study and combine study-level measures using random effects meta-analysis to allow the underlying true effect to vary across studies.[80] In the one-stage models, we will include study-specific intercepts to quantify and account for between-study variation in baseline risk. We will use random slopes to allow the relation between certain cofactors and the risk of CZS to vary across populations.

Combining absolute measures of effect, like the risk difference, across studies may mask important differences in the baseline risk.[81] We will present estimates of the risk difference in a forest plot of study-level estimates without presenting a summary meta-analytic estimate. In both the one- and two-stage analyses, we will use log binomial regression models to estimate the relative risk of each binary outcome and will use log Poisson regression to estimate the relative risk if log binomial models fail to converge.[82 83] In the two-stage models, we will assess the potential for non-linear relationships between continuous exposures (viral load) and covariates (e.g. gestational age, maternal age) by using the Akaike information critiera to compare restricted cubic splines with 3 knots to exponential, quadratic, and linear terms. In the one-stage models, we will use generalized additive mixed models (GAMMs) to assess potential non-linearities as the GAMM random smoothing parameter addresses the bias/variance trade-off by penalizing the added complexity from non-linear terms while accounting for between-study variation in non-linear effects.[84]

Joint estimation of multiple nested or otherwise related outcomes (multivariate meta-analysis)

Not all studies will have measured all primary or secondary outcomes of interest. For example, most studies will have measured ventriculomegaly, but may not include values for intracranial calcification or ocular abnormalities.[9] This analysis is intended to increase the precision of estimates of the spectrum of CZS abnormalities. Studies that do not include the measurement of a given outcome will necessarily be excluded from univariate estimates of that outcome, but will be included in multivariate models that estimate the joint probability of related outcomes. In the multivariate models, we will assume that the outcomes that are excluded from certain studies are missing at random and will incorporate studies by setting the missing observations and within-study correlations between outcomes to zero and will set the within-study variance to a very high number such that the artificial value that acts as a substitute for the missing outcome will have a negligible effect on the meta-analytic estimate from the multivariate model.[85] Alternatively, under a Bayesian framework, we will model a joint distribution for studies providing multiple outcomes and a univariate distribution for studies providing a single outcome without needing to address the missing within-study correlations and variance for studies with only one outcome.[86] The secondary outcomes that will be included in the multivariate analysis are listed in Table 1.

For peer review only - http://bmjopen.bmj.com/site/about/guidelines.xhtml

We will compare generalized linear mixed models (GLMMs) where we use one model to analyze nested or otherwise related outcomes to the standard univariate approach where we apply a separate model to analyze each outcome. Multivariate meta-analysis allows for the estimation of joint probabilities across multiple outcomes and accounts for cross- and within-study correlation between related outcomes.[85 87] Modelling several outcomes simultaneously improves the precision over univariate models by sharing information about heterogeneity and the average effect of the treatment which may facilitate inference about the relation between different CZS-related outcomes[85 88 89] (i.e. vermian dysgenesis and ocular abnormalities).

Multivariate model to combine estimates from fully and partially adjusted studies

A number of longitudinal studies will not include the minimal sufficient set of confounders. Estimates from partially adjusted studies (that are missing values for important confounders) will be combined with fully adjusted estimates in a one-stage multivariate meta-analysis. The one-stage multivariate model allows us to borrow information from partially adjusted studies with different sets of confounders while ensuring that we control for important confounders.[85 88]

Special considerations for the meta-analysis of cohort studies with rare events

Two-stage meta-analytic methods are based on large sample approximations, and may be unsuitable in the context of CZS, which can be considered a rare event.[90 91] Two-stage meta-analysis may be biased when small studies are included, the effect of an exposure is very large, or the outcome is rare, all of which may affect this analysis.[92] We will highlight any instances when the two-stage meta-analytic estimates may be biased by the aforementioned issues and will limit our inference to one-stage analyses in those cases. If we have a number of longitudinal studies with zero events, we will focus our inference on a one-stage approach to avoid reliance on large sample approximations.

Assessment of study- and participant-level heterogeneity

Separating within- and between- study heterogeneity is central to assessing participant-level heterogeneity and to understanding the relative importance of different potential effect measure modifiers.[50] We are only able to separate within- and between-study heterogeneity across studies that include both levels of the effect measure modifier of interest. The presence of clinical illness may be related to disease course through viral load or be a marker for the strength of the immune system's response to infection. We will conduct a one-stage analysis of longitudinal studies that include both symptomatic and asymptomatic women to assess whether the risk of CZS or of the most severe effects of congenital infection (miscarriage, fetal loss) differs for clinical and subclinical infections. Between-study heterogeneity is reflective of study-level differences, while within-study heterogeneity may be indicative of clinically important differences. We will mean center covariates included in the interaction terms at the study level to separate between- and within-study heterogeneity in our one-stage meta-

For peer review only - http://bmjopen.bmj.com/site/about/guidelines.xhtml

analytic estimates of how prior or co-infection with alpha or flaviviruses or STORCH pathogens modifies the effect of ZIKV infection.[93]

Heterogeneity in effect estimates will arise from clinically important differences between congenital infections or women (effect measure modification) and from study-level differences in exposure and outcome ascertainment (measurement error). With IPD, we are able to jointly assess study- and participant-level heterogeneity.[52] We will incorporate participant-level interaction terms in a one-stage analysis that includes random intercepts to account for unmeasured study-level factors. We will consider random slopes for certain covariates to allow for between-study variation in covariate effects across studies. Given the difficulty in assessing the total degrees of freedom in mixed models, we will apply bootstrapping to assess the approximate confidence intervals of the pooled interaction terms. We will present the analysis of effect measure modifiers in accordance with the revised STROBE guidelines.[94]

Based on our review of research protocols for planned or ongoing cohort studies, we expect to include data from longitudinal studies with different enrollment criteria, exposure and outcome ascertainment, diagnostic assays for prior- or co-infections, and measurement of important cofactors. We will include measures of study-level sources of heterogeneity (e.g. diagnostic assay, outcome definitions) as covariates in the one-stage regression to assess the variance explained by these factors. We will perform a sensitivity analysis where we limit our inference to studies with similar inclusion criteria and exposure, cofactor, and outcome ascertainment to reduce spurious cross-study heterogeneity. While two-stage analyses of interaction effects that fail to separate between- and within-study heterogeneity are subject to ecological bias[93] and our inference about the importance of interaction terms will primarily be derived from one-stage analyses, we will use a two-stage analysis to compare the magnitude of the interaction effects across studies. The interaction between certain cofactors and ZIKV exposure may not be consistent across studies. In the first stage of the two-stage analysis, we will use the likelihood ratio test (P-value < 0.05) to assess the importance of including interaction terms within each study. Individual cohort studies may not have the sample size needed to detect clinically important interactions between ZIKV and important cofactors. We will also assess whether a certain interaction is consistent across studies, while not necessarily statistically significant within individual studies.

Meta-regression and subgroup analyses have limited power to detect interactions and can only be used to make inference about the relation between the exposure and study-level, average values of participant characteristics.[92 95] Studies that are not willing or able to provide participant-level data may differ importantly from longitudinal studies whose data is included in the IPD-MA. We will apply subgroup analysis to a two-stage analysis of effect estimates from studies included in the IPD-MA and published estimates from studies that did not participate in the IPD-MA to assess whether study-level variation in recruitment and enrollment criteria, exposure and outcome ascertainment, and measurement of co-infections and other cofactors are important sources of heterogeneity in the pooled estimates. Some sources of heterogeneity (e.g. vector density and feeding patterns; DENV serotype) may not be measured and should be considered in sensitivity analyses.

For peer review only - http://bmjopen.bmj.com/site/about/guidelines.xhtml

Objective 3. *Use information on the relative importance of different effect measure modifiers identified in Objective 2 to decompose the total effect of ZIKV infection during pregnancy on adverse fetal, infant, and child outcomes.*

Some studies suggest that antibody-dependent enhancement related to concurrent or prior DENV infection or Japanese encephalitis vaccination may modify the effect of ZIKV infection on fetal development. Both the timing of exposure to DENV and DENV serotype may contribute to regional differences in the strength of the relation between ZIKV infection and CZS.[28 32] If we find evidence in the literature that the effect measure modifier identified in Objective 2 (e.g. DENV) may affect the outcome (e.g. CZS), we will apply inverse probability of treatment weighted-marginal structural models to decompose the total effect of ZIKV on the outcome of interest into the direct effects of ZIKV infection, the effect of ZIKV infection mediated by the posited effect measure modifier, and the effect of the interaction between ZIKV and the effect measure modifier.[96 97]

Objective 4. *Develop and validate a risk prediction tool to inform decision making by pregnant women, couples planning a pregnancy, and healthcare providers, and/or resource mobilization*

We will fit one-stage logistic regression models with random intercepts to account for differences in the baseline risk within each study. We will apply group Lasso regression[98] to identify the prognostic variables that predict progression to miscarriage, fetal loss, and microcephaly. Lasso regression is implemented using L1-penalized estimation. The application of group Lasso ensures that the algorithm selects all levels of categorical variables by treating corresponding dummy variables as a group instead of allowing the model to only select certain levels of categorical variables.[99 100] The L-1 penalty term allows for concurrent consideration of predictors and shrinkage, which facilitates variable selection in the context of high dimensional data.[101] We will standardize included variables so that all variables use the same scale. We will adopt cross-validation on the study level to select the optimal tuning parameter (λ) and will adopt restricted maximum likelihood (REML) to estimate the variance-covariance matrix of the study-level random effects.

Not all studies will have the resources to implement the most accurate and reliable ZIKV-related diagnostic tools. As part of the data synthesis, we will identify the exposure and cofactor diagnostic methods that are most commonly applied. As a sensitivity analysis, we will use these diagnostic methods to develop a risk prediction model so that the model can be applied in regular clinical practice.

Development and external validation of the prediction model

We will apply internal-external cross-validation wherein we rotate the cohort that is used for external validation to improve the model's predictive ability.[102] For example, given k cohort studies, we will use $k-1$ cohort studies to develop the prediction model and will validate model performance by applying the prediction model to a cohort that was not used to develop the prediction model. Internal-external

For peer review only - http://bmjopen.bmj.com/site/about/guidelines.xhtml

cross-validation allows for the use of all available data for model development and validation which improves model performance and generalizability.[103]

Evaluation of model performance

We will generate receiver operating characteristic (ROC) curves[104 105] in the cohort that was not used to develop the prediction model to estimate the model's true-positive (sensitivity) versus false-positive (1-specificity) rate for each binary outcome. These curves will then be summarized using the area under the ROC curve (AUC). In some instances, the pregnant woman or couple planning a pregnancy may prefer a more sensitive rather than a more specific model. We will present a range of cut-off values that maximize sensitivity, specificity, or both sensitivity and specificity to facilitate decision making by pregnant women or couples planning a pregnancy. We will assess the extent to which these thresholds yield consistent sensitivity and specificity across different regions and populations. We will use calibration plots to compare the observed and predicted probability of the outcome of interest within risk quintiles, and summarize these plots by calculating the total ratio of observed versus expected events (O:E ratio) and the calibration slope. Internal-external cross-validation of k studies will result in k AUCs, O:E ratios, and calibration slopes. We will apply random effects meta-analysis to combine estimates of the discrimination and calibration of the k predictive models. We will assess model calibration and discrimination and choose the model with the best properties.[102 106] We will use bootstrap validation to evaluate model optimism and will follow the TRIPOD statement guidelines for reporting the final prediction models.[107]

Step 4. Quantitative bias analysis

Given the complexity and level of measurement error, we will conduct a quantitative bias analysis under a Bayesian framework where we use a combination of expert opinion, laboratory EQA, and external and internal assessment of the relative accuracy of diagnostic assays and other methods for cofactor and outcome ascertainment to inform the prior distributions of bias parameters. Where possible, we will apply frequentist methods for quantitative bias analysis[108] as a sensitivity analysis and will use the GRADE criteria[109] to compare the quality of the evidence from Bayesian and frequentist models, with a focus on how imprecision, inconsistency, indirectness, magnitude of effect differ in the Bayesian and frequentist approaches to addressing the myriad sources of bias expected to affect these analyses.

Selection bias

Studies or surveillance systems that only recruit or test symptomatic pregnant women or studies that only enrolled pregnant women who tested positive for ZIKV infection are affected by selection bias because selection into the study is associated with the exposure.[63] This situation is similar to the inclusion of a single treatment arm in a randomized controlled trial. Although data from studies that only enroll pregnant women who test positive for ZIKV cannot directly inform estimates of the causal effect of ZIKV, these data can inform the development of prediction models because they contain information on the prognosis of ZIKV positive women. Longitudinal studies that restrict enrollment to

For peer review only - http://bmjopen.bmj.com/site/about/guidelines.xhtml

ZIKV positive pregnant women may also increase the precision of relative treatment effects by providing more events within ZIKV-exposed pregnant women. Longitudinal studies have reported that women who perceive their infants as unaffected by CZS are less likely to participate in follow-up. We will consider matching on the propensity score or the use of inverse probability of censoring weights[110] and prognostic score analysis[111] to account for measured determinants of differential loss to follow-up in the etiologic and prognostic models, respectively. Selection bias can be induced when we inappropriately adjust for a time-varying confounder affected by prior exposure (a confounder that also acts to mediate the relation between Zika virus infection and adverse fetal, infant, or child outcomes). We will use G-computation methods to appropriately adjust for time-dependent confounders affected by prior exposure.[112]

Confounding bias

We will adjust for confounders that are unlikely to mediate the causal relation between infection during pregnancy and adverse infant outcomes (Table 1). We will estimate each participant's likelihood of being infected during pregnancy, conditional on the study group and important confounders, to identify possible violations of the positivity assumption. In sensitivity analyses, we will apply propensity score matching within studies to ensure that important confounders are adequately balanced across exposure groups. Despite the prospective, collaborative development of a standardized research protocol for ZIKV cohort studies of pregnant women, confounders and effect measure modifiers may be defined differently across studies or not measured in certain studies. We will develop a detailed codebook that reflects the heterogeneity in confounder definitions and report on this heterogeneity in our analyses.

Measurement (i.e. detection, misclassification) bias

Despite efforts to harmonize case definitions across studies with the prospective development of a standardized protocol for cohorts of pregnant women and their infants,[60] the case definitions, diagnostic tools, and algorithms used to ascertain ZIKV infection, cofactors, and CZS-associated outcomes vary across studies.[113] The literature on the accuracy of ZIKV- and DENV-related assays is evolving rapidly.[30 114] Prior to initiating our analyses, we will synthesize the current evidence on the sensitivity and specificity of different assays for ZIKV diagnosis, for the assessment of concurrent or prior DENV infections, and for estimating the time of infection, amongst other relevant factors. The WHO standardized protocol for ZIKV-related cohorts of pregnant women includes WHO recommendations on the screening and assessment of neonates and infants with intrauterine ZIKV exposure;[115] we will compare study-level outcome definitions with the standardized WHO definitions. The role of heterogeneity related to case definitions and diagnostic tools will be explored through both frequentist and Bayesian methods. In the frequentist approach, we will: 1) include categorical or continuous markers of sensitivity and specificity of diagnostic tools as study-level covariates in the one-stage analyses and 2) apply diagnostic tool specific-subgroup analysis to both the one- and two-stage meta-analysis of effect measures from different studies. In the Bayesian approach, we will use a combination of expert opinion and data from external and internal validation studies to inform the probability distributions of bias parameters.[116]

Missing data

For peer review only - http://bmjopen.bmj.com/site/about/guidelines.xhtml

Missing data at the study level, as when confounders are not measured in certain studies, is a well-known challenge of IPD-MA[117 118] and a likely source of residual confounding. In keeping with current recommendations for addressing missingness in IPD-MA, we will apply new methods for multilevel multiple imputation to account for missing values.[119] As a sensitivity analysis, we will impute missing participant-level data in each study separately and use multivariate meta-analysis to combine data across studies that have and have not measured important host- and environmental-level cofactors.

Publication bias

IPD-MA may have a lower risk of publication bias than AD-MA because they include data from unpublished studies.[117] We have tried to ensure that the ZIKV IPD Consortium includes representatives from all of the academic and government institutions responsible for planned or ongoing ZIKV-related longitudinal studies of pregnant women and their infants. We expect that Consortium members will identify most ZIKV longitudinal studies and active surveillance systems of pregnant women and their infants, regardless of publication status, and we will conduct a systematic review to identify additional longitudinal studies and active surveillance systems. The degree of publication bias will be assessed visually by reviewing the asymmetry of study-level estimates from published and unpublished studies using funnel plots that compare log RR to the corresponding studies' sample size.[120]

We will convene a group of patient advocates to evaluate the ethical implications and utility of the risk stratification tool.

DISCUSSION

The application of IPD-MA to an emerging pathogen presents an important opportunity to harness global collaboration to inform the development of recommendations for pregnant women, couples planning a pregnancy, and public health practitioners. While IPD-MA offers real benefits compared to AD-MA or to the inference possible with individual cohort studies, the ability of IPD-MA to inform public health practice is directly related to the quality of the exposure, cofactor, and outcome ascertainment in the original cohort studies. Statistical methods for IPD-MA were developed in the context of clinical research and randomized control trials. These methods needs to be adapted to account for the myriad sources of uncertainty and bias that affect observational research, especially for field epidemiology studies conducted as part of the research response to unknown or emerging pathogens.

Historically, arboviruses and other neglected tropical diseases have been understudied because the burden of disease falls on under resourced populations in the Global South[121] In the context of ZIKV, the unequal distribution of risk is coupled with inequities in access to preventative measures like modern contraception and to critical clinical and therapeutic care for infants affected by microcephaly and ZIKV-related neurological disorders. Each case of microcephaly is associated with a loss of 29.95 DALYs and treatment costs ranging from 91K to 1 million USD.[122] To put these figures into perspective, the yearly

For peer review only - http://bmjopen.bmj.com/site/about/guidelines.xhtml

per capita income in Pernambuco, the Brazilian state with one of the highest burdens of CZS, is 3,471 USD.[123]

There is no vaccine for ZIKV and the only treatment is supportive.[58] There have been numerous calls for data sharing[124 125] and cooperation between governments and academic institutions,[54 126] and public and private charities have pledged significant financial support to improve our understanding of ZIKV epidemiology and to develop a vaccine or small molecule prophylaxis to decrease the risk of infection. In the wake of the Ebola epidemic, the global response to ZIKV has been characterized by unprecedented levels of international cooperation. In the absence of a ZIKV vaccine or prophylaxis, international leaders in ZIKV research have formed the ZIKV IPD Consortium to identify, collect, and synthesize IPD from longitudinal studies of pregnant women that measure ZIKV infection during pregnancy and fetal, infant, and child outcomes. This data will be used to quantify the absolute risk of ZIKV-related pregnancy complications with the goal of aiding women and their families in making difficult reproductive decisions and with helping public health systems prevent and quantify the burden of congenital Zika infection.

Challenges of developing and conducting an individual participant data-meta-analysis in the context of an emerging pathogen

Ideally, researchers pre-specify confounders, effect measure modifiers and plans for subgroup or sensitivity analyses in their research protocol. In the context of Zika, our understanding of the virus is changing so rapidly that analysis plans may change significantly despite our best efforts to review the latest evidence on transmission, immunological response, diagnostic assays, vector biology, and basic ZIKV epidemiology. Our ability to appropriately account for measurement error will play a critical role in the accuracy of estimates for the risk of CZS and other adverse fetal, infant, and child outcomes. This is one of the first instances where an IPD-MA has been used to address public health concerns in the context of an emerging pathogen. We expect that best practices and lessons learned from this IPD-MA can be used to facilitate the formation of research collaborations to streamline the public health response to future epidemics.

Patient and Public Involvement

In keeping with guidelines for public involvement in research,[127] knowledge users (i.e. women of reproductive age and their families, clinicians) will be consulted at each stage of this research. The research question and protocol were designed with feedback from clinicians who treat pregnant women in ZIKV-endemic areas and infants and children affected by CZS. Focus groups that include women of reproductive age in ZIKV-endemic areas will be used to evaluate the ethical implications and utility of the risk stratification tool in three countries.

ETHICS AND DISSEMINATION

This IPD-MA protocol has been deemed exempt from ethical review by the WHO Ethics Review Committee and the Emory University Institutional Review Board. Individual longitudinal studies will

For peer review only - http://bmjopen.bmj.com/site/about/guidelines.xhtml

provide documentation of ethics review prior to sharing their de-identified, participant-level data. The WHO has developed guidance for data sharing in public health emergencies or in the context of emerging pathogens.[128] Sharing de-identified data for IPD-MA is generally considered exempt from ethical review if the objectives of the IPD-MA are in keeping with the objectives of the original studies.[129] Individual research studies and consortia will secure additional ethics review and/or legal guidance on the sharing of de-identified, subject-level data as needed. The results of this analysis will be published under the ZIKV IPD Consortium name and will include a list of the names of key investigators from each study that contributed data for that analysis and researchers who contributed to the analysis or writing at the end of the publication. Findings from the proposed analysis will be shared via national and international conferences; existing platforms for dissemination of ZIKV-related research (e.g. The Global Health Network); and through publication in open access, peer-reviewed journals.

Contributors

NB, CH, TJ, NL, LM, JPS, LR contributed to the initial conception of the study. AB, TPAD, PG, NL, LM, YW made substantial contributions to the statistical methodology proposed for the IPD-MA. LM wrote the first draft of the protocol. AWS, YW, TVBA, MV, CMTM, MDT, MT, AT, PS, JPS, ASA, CSS, AMS, NSC, KDR, LR, APB, LP, LEPR, FP, SP, MN, TN, MEM, IM, MCMM, DBMF, LM, CM, NL, ZL, ADL, MK, CK, EJ, TJ, CH, PG, PG, JG, ACFD, VE, GD, TPAD, MLC, PB, NB, EB, PB, FB, SB, AB, VAS, RAAX, AAC, JA provided substantial revisions to the protocol. All authors approved the final version of the protocol.

Acknowledgments

The authors would like to acknowledge the valuable contributions of Liège Maria Abreu de Carvalho, Rosangela Batista, Ana Paula Bertozzi, Gabriel Carles, Denise Cotrim, Luana Damasceno, Lady Dimitrakis, María Manoela Duarte Rodrigues, Cassia F. Estofolete, Maria Isabel Fragoso da Silveira Gouvêa, Vicky Fumadó-Pérez, Rosa Estela Gazeta, Neely Kaydos-Daniels, Suzanne Gilboa, Amy Krystosik, Véronique Lambert, Milagros García López-Hortelano, Marisa Marcia Mussi-Pinhata, Christina Nelson, Karin Nielsen, Denise M. Oliani, Renata Rabello, Marizelia Ribeiro, Barry Rockx, Laura C. Rodrigues, Silvia Salgado, Katia Silveira, Elena Sulleiro, Van Tong, Diana Valencia, Wayner Vieira de Souza, Luis Angel Villar Centeno, and Andrea Zin to the review of the ZIKV IPD Consortium IPD-MA Protocol. In addition, the authors would like to acknowledge the BMJ Open reviewers for their thoughtful comments on the protocol.

Funding

The development of the IPD-MA protocol was supported by a Wellcome Trust grant to the WHO Department of Reproductive Health and Research Human Reproduction Programme, grant number 206532/Z/17/Z.

Disclaimer

The manuscript contents are the responsibility of the authors and do not reflect the views of the Wellcome Trust.

Competing Interests

None declared

For peer review only - http://bmjopen.bmj.com/site/about/guidelines.xhtml

Provenance and peer review

Not commissioned; externally peer reviewed.

Data sharing statement

Not all investigators are willing to share study for analyses beyond what has been proposed here. Governance issues related to sharing the de-identified, participant-level data used in the proposed analyses will be described in the manuscripts that present the results of the proposed analyses.

References

1. Krauer F, Riesen M, Reveiz L, et al. Zika Virus Infection as a Cause of Congenital Brain Abnormalities and Guillain–Barré Syndrome: Systematic Review. *PLOS Medicine* 2017;14(1):e1002203. doi: 10.1371/journal.pmed.1002203
2. Rasmussen SA, Jamieson DJ, Honein MA, et al. Zika Virus and Birth Defects — Reviewing the Evidence for Causality. *New England Journal of Medicine* 2016;374(20):1981-87. doi: doi:10.1056/NEJMsr1604338
3. Bhatnagar J, Rabeneck D, Martines R, et al. Zika Virus RNA Replication and Persistence in Brain and Placental Tissue. *Emerging Infectious Diseases* 2017;23(3)
4. Garcez PP, Loiola EC, Madeiro da Costa R, et al. Zika virus impairs growth in human neurospheres and brain organoids. *Science* 2016 doi: 10.1126/science.aaf6116
5. Dang J, Tiwari SK, Lichinchi G, et al. Zika Virus Depletes Neural Progenitors in Human Cerebral Organoids through Activation of the Innate Immune Receptor TLR3. *Cell Stem Cell* 2016;19(2):258-65. doi: https://doi.org/10.1016/j.stem.2016.04.014
6. Tang H, Hammack C, Ogden Sarah C, et al. Zika Virus Infects Human Cortical Neural Progenitors and Attenuates Their Growth. *Cell Stem Cell* 2016;18(5):587-90. doi: https://doi.org/10.1016/j.stem.2016.02.016
7. Opitz J, Holt M. Microcephaly: general considerations and aids to nosology. *Journal of craniofacial genetics and developmental biology* 1990;10(2):175-204.
8. Passemard S, Kaindl AM, Verloes A. Microcephaly. Handbook of clinical neurology: Elsevier 2013:129-41.
9. Melo A, Aguiar R, Amorim M, et al. Congenital zika virus infection: Beyond neonatal microcephaly. *JAMA Neurology* 2016 doi: 10.1001/jamaneurol.2016.3720
10. Coyne CB, Lazear HM. Zika virus [mdash] reigniting the TORCH. *Nat Rev Micro* 2016;14(11):707-15. doi: 10.1038/nrmicro.2016.125
11. Kaindl AM, Passemard S, Kumar P, et al. Many roads lead to primary autosomal recessive microcephaly. *Progress in Neurobiology* 2010;90(3):363-83. doi: http://dx.doi.org/10.1016/j.pneurobio.2009.11.002
12. Chan JFW, Choi GKY, Yip CCY, et al. Zika fever and congenital Zika syndrome: An unexpected emerging arboviral disease. *Journal of Infection* 2016;72(5):507-24. doi: http://dx.doi.org/10.1016/j.jinf.2016.02.011
13. de Araújo TVB, Ximenes RAdA, Miranda-Filho DdB, et al. Association between microcephaly, Zika virus infection, and other risk factors in Brazil: final report of a case-control study. *The Lancet Infectious Diseases* 2017 doi: https://doi.org/10.1016/S1473-3099(17)30727-2
14. Krow-Lucal ER, de Andrade MR, Cananéa JNA, et al. Association and birth prevalence of microcephaly attributable to Zika virus infection among infants in Paraíba, Brazil, in 2015–16: a case-control study. *The Lancet Child & Adolescent Health* 2018 doi: https://doi.org/10.1016/S2352-4642(18)30020-8

For peer review only - http://bmjopen.bmj.com/site/about/guidelines.xhtml

15. Brasil P, Pereira JPJ, Moreira ME, et al. Zika Virus Infection in Pregnant Women in Rio de Janeiro. *New England Journal of Medicine* 2016;375(24):2321-34. doi: doi:10.1056/NEJMoa1602412
16. Hoen B, Schaub B, Funk AL, et al. Pregnancy Outcomes after ZIKV Infection in French Territories in the Americas. *New England Journal of Medicine* 2018;378(11):985-94. doi: 10.1056/NEJMoa1709481
17. Honein MA, Dawson AL, Petersen EE, et al. Birth defects among fetuses and infants of us women with evidence of possible zika virus infection during pregnancy. *JAMA* 2017;317(1):59-68. doi: 10.1001/jama.2016.19006
18. Osuna CE, Whitney JB. Nonhuman Primate Models of Zika Virus Infection, Immunity, and Therapeutic Development. *The Journal of Infectious Diseases* 2017;216(suppl_10):S928-S34. doi: 10.1093/infdis/jix540
19. Musso D, Cao-Lormeau VM, Gubler DJ. Zika virus: following the path of dengue and chikungunya? *The Lancet* 2015;386(9990):243-44. doi: 10.1016/S0140-6736(15)61273-9
20. Araujo LM, Ferreira MLB, Nascimento OJ. Guillain-Barré syndrome associated with the Zika virus outbreak in Brazil. *Arquivos de Neuro-Psiquiatria* 2016;74:253-55.
21. Parra B, Lizarazo J, Jiménez-Arango JA, et al. Guillain–Barré Syndrome Associated with Zika Virus Infection in Colombia. *New England Journal of Medicine* 2016;375(16):1513-23. doi: 10.1056/NEJMoa1605564
22. Pacheco O, Beltrán M, Nelson CA, et al. Zika Virus Disease in Colombia — Preliminary Report. *New England Journal of Medicine* 2016;0(0) doi: 10.1056/NEJMoa1604037
23. Cauchemez S, Besnard M, Bompard P, et al. Association between Zika virus and microcephaly in French Polynesia, 2013–15: a retrospective study. *The Lancet* 2016;387(10033):2125-32. doi: http://dx.doi.org/10.1016/S0140-6736(16)00651-6
24. Cao-Lormeau V-M, Blake A, Mons S, et al. Guillain-Barré Syndrome outbreak associated with Zika virus infection in French Polynesia: a case-control study. *The Lancet* 2016;387(10027):1531-39. doi: http://dx.doi.org/10.1016/S0140-6736(16)00562-6
25. Epidemiological Alert: Zika virus infection: Pan American Health Organization, 2016:1-8.
26. Lowe R, Barcellos C, Brasil P, et al. The Zika Virus Epidemic in Brazil: From Discovery to Future Implications. *International Journal of Environmental Research and Public Health* 2018;15(1):96. doi: 10.3390/ijerph15010096
27. Zika virus infection: global update on epidemiology and potentially associated clinical manifestations. Wkly Epidemiol Rec: WHO Press, 2016:73-81.
28. Cruz MM, Puerta-Guardo HN, Schildhauer S, et al. Immune cross-reactivity between Dengue and Zika viruses in two pediatric studies in Nicaragua. *The Journal of Immunology* 2017;198(1 Supplement):210.5-10.5.
29. Castanha PMS, Nascimento EJM, Braga C, et al. Dengue Virus–Specific Antibodies Enhance Brazilian Zika Virus Infection. *The Journal of Infectious Diseases* 2017;215(5):781-85. doi: 10.1093/infdis/jiw638
30. Goncalves A, Peeling RW, Chu MC, et al. Innovative and New Approaches to Laboratory Diagnosis of Zika and Dengue: A Meeting Report. *The Journal of Infectious Diseases* 2017:jix678-jix78. doi: 10.1093/infdis/jix678
31. Turner LH, Kinder JM, Wilburn A, et al. Preconceptual Zika virus asymptomatic infection protects against secondary prenatal infection. *PLoS Pathogens* 2017;13(11):e1006684. doi: 10.1371/journal.ppat.1006684
32. Andrade DV, Harris E. Recent advances in understanding the adaptive immune response to Zika virus and the effect of previous flavivirus exposure. *Virus Research* 2017 doi: https://doi.org/10.1016/j.virusres.2017.06.019

For peer review only - http://bmjopen.bmj.com/site/about/guidelines.xhtml

33. Andrade PE, Coloma J, Michlmayr D, et al. Zika and Dengue Virus Specific and Cross-reactive Memory B Cell Responses. *The Journal of Immunology* 2017;198(1 Supplement):214.7-14.7.
34. Jaenisch T, Rosenberger KD, Brito C, et al. Estimating the risk for microcephaly after Zika virus infection in Brazil. *Bulletin of the World Health Organization* 2016 doi: http://dx.doi.org/10.2471/BLT.16.178608
35. Butler D. First Zika-linked birth defects detected in Colombia. *Nature* 2016;531(7593):153.
36. Costa F, Ko AI. Zika virus and microcephaly: where do we go from here? *The Lancet Infectious Diseases* 2018;18(3):236-37. doi: https://doi.org/10.1016/S1473-3099(17)30697-7
37. YKY C, TY L, TTH L, et al. Impact of replacing Chinese ethnicity-specific fetal biometry charts with the INTERGROWTH-21st standard. *BJOG: An International Journal of Obstetrics & Gynaecology* 2016;123(S3):48-55. doi: doi:10.1111/1471-0528.14008
38. Leibovitz Z, Daniel-Spiegel E, Malinger G, et al. Prediction of microcephaly at birth using three reference ranges for fetal head circumference: can we improve prenatal diagnosis? *Ultrasound in Obstetrics & Gynecology* 2016;47(5):586-92. doi: doi:10.1002/uog.15801
39. Barchfield J. Officials in Brazil urge women to avoid pregnancy due to Zika virus: Boston Globe; 2016 [updated January 26, 2016. Available from: https://www.bostonglobe.com/news/world/2016/01/26/officials-brazil-urge-women-avoid-pregnancy-due-zika-virus/Ask7wAjozV0G6SCv80uK8N/story.html accessed June 4, 2018 2018.
40. Brodzinsky S. Rights groups denounce Zika advice to avoid pregnancy in Latin America London, England: Guardian; 2016 [updated January 27, 2016. Available from: https://www.theguardian.com/global-development/2016/jan/27/rights-groups-denounce-zika-advice-to-avoid-pregnancy-in-latin-america accessed June 4, 2018 2018.
41. Wilder-Smith A, Gubler DJ, Weaver SC, et al. Epidemic arboviral diseases: priorities for research and public health. *The Lancet Infectious Diseases* 2017;17(3):e101-e06. doi: https://doi.org/10.1016/S1473-3099(16)30518-7
42. Aiken ARA, Scott JG, Gomperts R, et al. Requests for Abortion in Latin America Related to Concern about Zika Virus Exposure. *New England Journal of Medicine* 2016;375(4):396-98. doi: 10.1056/NEJMc1605389
43. Stern AM. Zika and reproductive justice. *Cadernos de Saúde Pública* 2016;32
44. Debray TPA, Riley RD, Rovers MM, et al. Individual Participant Data (IPD) Meta-analyses of Diagnostic and Prognostic Modeling Studies: Guidance on Their Use. *PLoS Med* 2015;12(10):e1001886. doi: 10.1371/journal.pmed.1001886
45. Riley RD, Ensor J, Snell KIE, et al. External validation of clinical prediction models using big datasets from e-health records or IPD meta-analysis: opportunities and challenges. *BMJ* 2016;353 doi: 10.1136/bmj.i3140
46. Weaver SC, Costa F, Garcia-Blanco MA, et al. Zika virus: History, emergence, biology, and prospects for control. *Antiviral Research* 2016;130:69-80. doi: http://dx.doi.org/10.1016/j.antiviral.2016.03.010
47. Alphey L, Benedict M, Bellini R, et al. Sterile-insect methods for control of mosquito-borne diseases: an analysis. *Vector-Borne and Zoonotic Diseases* 2010;10(3):295-311.
48. Yakob L, Walker T. Zika virus outbreak in the Americas: the need for novel mosquito control methods. *The Lancet Global Health* 2016;4(3):e148-e49. doi: http://dx.doi.org/10.1016/S2214-109X(16)00048-6
49. Fernandes M, Stein A, Newton CR, et al. The INTERGROWTH-21st Project Neurodevelopment Package: A Novel Method for the Multi-Dimensional Assessment of Neurodevelopment in Pre-School Age Children. *PLOS ONE* 2014;9(11):e113360. doi: 10.1371/journal.pone.0113360

For peer review only - http://bmjopen.bmj.com/site/about/guidelines.xhtml

50. Riley RD, Lambert PC, Staessen JA, et al. Meta-analysis of continuous outcomes combining individual patient data and aggregate data. *Statistics in Medicine* 2008;27(11):1870-93. doi: 10.1002/sim.3165
51. Debray TPA, Moons KGM, Abo-Zaid GMA, et al. Individual Participant Data Meta-Analysis for a Binary Outcome: One-Stage or Two-Stage? *PLoS ONE* 2013;8(4):e60650. doi: 10.1371/journal.pone.0060650
52. Debray TPA, Moons KGM, van Valkenhoef G, et al. Get real in individual participant data (IPD) meta-analysis: a review of the methodology. *Research Synthesis Methods* 2015;6(4):293-309. doi: 10.1002/jrsm.1160
53. Morabito KM, Graham BS. Zika Virus Vaccine Development. *The Journal of Infectious Diseases* 2017;216(suppl_10):S957-S63. doi: 10.1093/infdis/jix464
54. Hotez PJ. What does zika virus mean for the children of the Americas? *JAMA Pediatrics* 2016;170(8):787-89. doi: 10.1001/jamapediatrics.2016.1465
55. Dejnirattisai W, Supasa P, Wongwiwat W, et al. Dengue virus sero-cross-reactivity drives antibody-dependent enhancement of infection with zika virus. *Nat Immunol* 2016;17(9):1102-08. doi: 10.1038/ni.3515
56. Malone RW, Homan J, Callahan MV, et al. Zika Virus: Medical Countermeasure Development Challenges. *PLoS Negl Trop Dis* 2016;10(3):e0004530. doi: 10.1371/journal.pntd.0004530
57. Bardina SV, Bunduc P, Tripathi S, et al. Enhancement of Zika virus pathogenesis by preexisting antiflavivirus immunity. *Science* 2017;356(6334):175-80. doi: 10.1126/science.aal4365
58. Poland GA, Kennedy RB, Ovsyannikova IG, et al. Development of vaccines against Zika virus. *The Lancet Infectious Diseases* 2018;18(7):e211-e19. doi: 10.1016/S1473-3099(18)30063-X
59. Durbin AP, Whitehead SS. Zika Vaccines: Role for Controlled Human Infection. *The Journal of Infectious Diseases* 2017;216(suppl_10):S971-S75. doi: 10.1093/infdis/jix491
60. Van Kerkhove MD, Reveiz L, Souza JP, et al. Harmonisation of Zika virus research protocols to address key public health concerns. *The Lancet Global Health* 2016;4(12):e911-e12. doi: 10.1016/S2214-109X(16)30255-8
61. Moher D, Shamseer L, Clarke M, et al. Preferred reporting items for systematic review and meta-analysis protocols (PRISMA-P) 2015 statement. *Systematic Reviews* 2015;4(1):1.
62. Stewart LA, Clarke M, Rovers M, et al. Preferred reporting items for a systematic review and meta-analysis of individual participant data: The prisma-ipd statement. *JAMA* 2015;313(16):1657-65. doi: 10.1001/jama.2015.3656
63. Reveiz L, Haby MM, Martínez-Vega R, et al. Risk of bias and confounding of observational studies of Zika virus infection: A scoping review of research protocols. *PLOS ONE* 2017;12(7):e0180220. doi: 10.1371/journal.pone.0180220
64. Counotte M, Egli-Gany D, Riesen M, et al. Zika virus infection as a cause of congenital brain abnormalities and Guillain-Barré syndrome: From systematic review to living systematic review [version 1; referees: 1 approved with reservations]2018.
65. Jupyter Notebooks-a publishing format for reproducible computational workflows. ELPUB; 2016.
66. Kiserud T, Piaggio G, Carroli G, et al. The World Health Organization fetal growth charts: a multinational longitudinal study of ultrasound biometric measurements and estimated fetal weight. *PLoS Medicine* 2016:1-79.
67. Fenton TR, Kim JH. A systematic review and meta-analysis to revise the Fenton growth chart for preterm infants. *BMC Pediatrics* 2013;13(1):59. doi: 10.1186/1471-2431-13-59
68. Silverman JG, Decker MR, Reed E, et al. Intimate partner violence victimization prior to and during pregnancy among women residing in 26 U.S. states: Associations with maternal and neonatal health. *American Journal of Obstetrics and Gynecology* 2006;195(1):140-48. doi: https://doi.org/10.1016/j.ajog.2005.12.052

For peer review only - http://bmjopen.bmj.com/site/about/guidelines.xhtml

69. Bayley N. Bayley scales of infant development: Manual: Psychological Corporation 1993.
70. Squires J, Bricker DD, Twombly E. Ages & stages questionnaires: Paul H. Brookes Baltimore, MD 2009.
71. Reeves BC, Deeks JJ, Higgins JP. Chapter 13: Including non-randomized studies. In: Higgins JG, S, ed. Cochrane handbook for systematic reviews of interventions. Chichester (UK): John Wiley & Sons 2008.
72. Jarde A, Losilla J-M, Vives J, et al. Q-Coh: A tool to screen the methodological quality of cohort studies in systematic reviews and meta-analyses. *International Journal of Clinical and Health Psychology* 2013;13(2):138-46. doi: http://dx.doi.org/10.1016/S1697-2600(13)70017-6
73. Higgins J, Sterne J, Savović J, et al. A revised tool for assessing risk of bias in randomized trials. *Cochrane Database Syst Rev* 2016;10(Suppl 1):29-31.
74. Zika Virus: Information for Clinicians Atlanta, GA: Centers for Disease Control and Prevention; 2016 [updated 13 June 2016; cited 2016. Available from: https://www.cdc.gov/zika/pdfs/clinicianppt.pdf accessed 29 December 2016 2016.
75. Gómez G, Espinal A, W. Lagakos S. Inference for a linear regression model with an interval-censored covariate. *Statistics in Medicine* 2003;22(3):409-25. doi: 10.1002/sim.1326
76. Cortina-Borja M, Tan HK, Wallon M, et al. Prenatal Treatment for Serious Neurological Sequelae of Congenital Toxoplasmosis: An Observational Prospective Cohort Study. *PLOS Medicine* 2010;7(10):e1000351. doi: 10.1371/journal.pmed.1000351
77. Joseph KS, Kramer MS. The fetuses-at-risk approach: survival analysis from a fetal perspective. *Acta Obstetricia et Gynecologica Scandinavica* 2018;97(4):454-65. doi: doi:10.1111/aogs.13194
78. Marx A, Bucher HC. Numbers needed to treat derived from meta-analysis: a word of caution. *Evidence Based Medicine* 2003;8(2):36-37. doi: 10.1136/ebm.8.2.36
79. Schulz KF, Altman DG, Moher D. CONSORT 2010 Statement: updated guidelines for reporting parallel group randomised trials. *BMC Medicine* 2010;8(1):1-9. doi: 10.1186/1741-7015-8-18
80. Riley RD, Higgins JP, Deeks JJ. Interpretation of random effects meta-analyses. *BMJ* 2011;342 doi: 10.1136/bmj.d549
81. Egger M, Smith GD, Phillips AN. Meta-analysis: Principles and procedures. *BMJ* 1997;315(7121):1533-37. doi: 10.1136/bmj.315.7121.1533
82. Yelland LN, Salter AB, Ryan P. Performance of the Modified Poisson Regression Approach for Estimating Relative Risks From Clustered Prospective Data. *American Journal of Epidemiology* 2011;174(8):984-92. doi: 10.1093/aje/kwr183
83. Richardson DB, Kinlaw AC, MacLehose RF, et al. Standardized binomial models for risk or prevalence ratios and differences. *International journal of epidemiology* 2015;44(5):1660-72. doi: https://doi.org/10.1093/ije/dyv137
84. Wood SN. Low-Rank Scale-Invariant Tensor Product Smooths for Generalized Additive Mixed Models. *Biometrics* 2006;62(4):1025-36. doi: doi:10.1111/j.1541-0420.2006.00574.x
85. Riley RD, Price MJ, Jackson D, et al. Multivariate meta-analysis using individual participant data. *Research Synthesis Methods* 2015;6(2):157-74. doi: 10.1002/jrsm.1129
86. Wei Y, Higgins JPT. Bayesian multivariate meta-analysis with multiple outcomes. *Statistics in Medicine* 2013;32(17):2911-34. doi: doi:10.1002/sim.5745
87. Riley RD, Jackson D, Salanti G, et al. Multivariate and network meta-analysis of multiple outcomes and multiple treatments: rationale, concepts, and examples. *BMJ* 2017;358:j3932. doi: 10.1136/bmj.j3932
88. Debray TP, Schuit E, Efthimiou O, et al. An overview of methods for network meta-analysis using individual participant data: when do benefits arise? *Statistical Methods in Medical Research* 2018;27(5):1351-64. doi: 10.1177/0962280216660741

For peer review only - http://bmjopen.bmj.com/site/about/guidelines.xhtml

89. Jackson D, White IR, Price M, et al. Borrowing of strength and study weights in multivariate and network meta-analysis. *Statistical Methods in Medical Research* 2017;26(6):2853-68. doi: 10.1177/0962280215611702
90. Higgins J, Deeks JJ, Altman DG. 16.9.1 Meta-analysis of rare events. Cochrane handbook for systematic reviews of interventions: Cochrane book series2008:481-529.
91. Stijnen T, Hamza TH, Özdemir P. Random effects meta-analysis of event outcome in the framework of the generalized linear mixed model with applications in sparse data. *Statistics in Medicine* 2010;29(29):3046-67. doi: 10.1002/sim.4040
92. Tierney JF, Vale C, Riley R, et al. Individual Participant Data (IPD) Meta-analyses of Randomised Controlled Trials: Guidance on Their Use. *PLOS Medicine* 2015;12(7):e1001855. doi: 10.1371/journal.pmed.1001855
93. Fisher DJ, Copas AJ, Tierney JF, et al. A critical review of methods for the assessment of patient-level interactions in individual participant data meta-analysis of randomized trials, and guidance for practitioners. *J Clin Epidemiol* 2011;64(9):949-67. doi: 10.1016/j.jclinepi.2010.11.016 [published Online First: 2011/03/16]
94. Knol MJ, VanderWeele TJ. Recommendations for presenting analyses of effect modification and interaction. *International Journal of Epidemiology* 2012;41(2):514-20. doi: 10.1093/ije/dyr218
95. Koopman L, van der Heijden GJMG, Hoes AW, et al. Empirical comparison of subgroup effects in conventional and individual patient data meta-analyses. *International Journal of Technology Assessment in Health Care* 2008;24(3):358-61. doi: 10.1017/S0266462308080471
96. VanderWeele TJ. Marginal Structural Models for the Estimation of Direct and Indirect Effects. *Epidemiology* 2009;20(1):18-26 10.1097/EDE.0b013e31818f69ce.
97. VanderWeele TJ, Tchetgen Tchetgen EJ. Attributing effects to interactions. *Epidemiology (Cambridge, Mass)* 2014;25(5):711-22. doi: 10.1097/EDE.0000000000000096
98. Groll A, Groll MA. Package 'glmmLasso'. 2016
99. Yuan M, Lin Y. Model selection and estimation in regression with grouped variables. *Journal of the Royal Statistical Society: Series B (Statistical Methodology)* 2006;68(1):49-67. doi: 10.1111/j.1467-9868.2005.00532.x
100. Meier L, Van De Geer S, Bühlmann P. The group lasso for logistic regression. *Journal of the Royal Statistical Society: Series B (Statistical Methodology)* 2008;70(1):53-71. doi: 10.1111/j.1467-9868.2007.00627.x
101. Groll A, Tutz G. Variable selection for generalized linear mixed models by L 1-penalized estimation. *Statistics and Computing* 2014;24(2):137-54. doi: 10.1007/s11222-012-9359-z
102. Debray TPA, Moons KGM, Ahmed I, et al. A framework for developing, implementing, and evaluating clinical prediction models in an individual participant data meta-analysis. *Statistics in Medicine* 2013;32(18):3158-80. doi: 10.1002/sim.5732
103. Ahmed I, Debray TP, Moons KG, et al. Developing and validating risk prediction models in an individual participant data meta-analysis. *BMC Medical Research Methodology* 2014;14(1):3. doi: 10.1186/1471-2288-14-3
104. Hanley JA, McNeil BJ. The meaning and use of the area under a receiver operating characteristic (ROC) curve. *Radiology* 1982;143(1):29-36. doi: doi:10.1148/radiology.143.1.7063747
105. Steyerberg EW, Vickers AJ, Cook NR, et al. Assessing the performance of prediction models: a framework for some traditional and novel measures. *Epidemiology (Cambridge, Mass)* 2010;21(1):128-38. doi: 10.1097/EDE.0b013e3181c30fb2
106. Royston P, Parmar MKB, Sylvester R. Construction and validation of a prognostic model across several studies, with an application in superficial bladder cancer. *Statistics in Medicine* 2004;23(6):907-26. doi: doi:10.1002/sim.1691

For peer review only

For peer review only - http://bmjopen.bmj.com/site/about/guidelines.xhtml

107. Moons KM, Altman DG, Reitsma JB, et al. Transparent reporting of a multivariable prediction model for individual prognosis or diagnosis (tripod): Explanation and elaboration. *Annals of Internal Medicine* 2015;162(1):W1-W73. doi: 10.7326/M14-0698
108. Lash TL, Fox MP, Fink AK. Applying quantitative bias analysis to epidemiologic data. New York, New York: Springer Science & Business Media 2011.
109. Balshem H, Helfand M, Schünemann HJ, et al. GRADE guidelines: 3. Rating the quality of evidence. *Journal of clinical epidemiology* 2011;64(4):401-06.
110. Hernán MA, Hernandez-Diaz S, Robins JM. A structural approach to selection bias. *Epidemiology* 2004;15(5):615-25.
111. Hansen BB. The prognostic analogue of the propensity score. *Biometrika* 2008;95(2):481-88. doi: 10.1093/biomet/asn004
112. Mansournia MA, Etminan M, Danaei G, et al. Handling time varying confounding in observational research. *BMJ* 2017;359 doi: 10.1136/bmj.j4587
113. Sorvillo FJ, Morrison AC, Berlin OGW. Vector-Borne Transmission. In: Thomas JC, Weber DJ, eds. Epidemiologic methods for the study of infectious diseases. Oxford: Oxford University Press 2001:249-65.
114. Balmaseda A, Zambrana JV, Collado D, et al. Comparison of four serological methods and two RT-PCR assays for diagnosis and surveillance of Zika. *Journal of Clinical Microbiology* 2018 doi: 10.1128/JCM.01785-17
115. Screening, assessment and management of neonates and infants with complications associated with Zika virus exposure in utero: Interim guidance. 2016 30 August 2016. http://origin.who.int/csr/resources/publications/zika/assessment-infants/en/ (accessed 17 January 2017).
116. Gelman A, Carlin JB, Stern HS, et al. Bayesian data analysis: CRC press Boca Raton, FL 2014.
117. Riley RD, Lambert PC, Abo-Zaid G. Meta-analysis of individual participant data: rationale, conduct, and reporting. *BMJ: British Medical Journal* 2010;340(7745):521-25. doi: 10.2307/25674217
118. Burgess S, White IR, Resche-Rigon M, et al. Combining multiple imputation and meta-analysis with individual participant data. *Statistics in Medicine* 2013;32(26):4499-514. doi: 10.1002/sim.5844
119. Audigier V, White IR, Jolani S, et al. Multiple imputation for multilevel data with continuous and binary variables. *arXiv preprint arXiv:170200971* 2017
120. Peters JL, Sutton AJ, Jones DR, et al. Comparison of two methods to detect publication bias in meta-analysis. *JAMA* 2006;295(6):676-80. doi: 10.1001/jama.295.6.676
121. Hotez PJ, Fenwick A, Savioli L, et al. Rescuing the bottom billion through control of neglected tropical diseases. *The Lancet* 2009;373(9674):1570-75. doi: http://dx.doi.org/10.1016/S0140-6736(09)60233-6
122. Alfaro-Murillo JA, Parpia AS, Fitzpatrick MC, et al. A Cost-Effectiveness Tool for Informing Policies on Zika Virus Control. *PLoS Negl Trop Dis* 2016;10(5):e0004743. doi: 10.1371/journal.pntd.0004743
123. Pernambuco: Wikipedia; 2017 [updated 14 January 2017. Available from: https://en.wikipedia.org/wiki/Pernambuco accessed 17 January 2017 2017.
124. Sharing data during Zika and other global health emergencies: Wellcome Trust; 2016 [updated 10 February 2016. Available from: https://wellcome.ac.uk/news/sharing-data-during-zika-and-other-global-health-emergencies accessed 10 December 2016 2016.
125. Gérardin P, Randrianaivo H, Schaub B, et al. Congenital Zika syndrome: time to move from case series to case-control studies and data sharing. *BMJ* 2016;354 doi: 10.1136/bmj.i4850
126. The next steps on Zika. *Nature* 2016;530:5.
127. Popay J, Collins M, Group wtPS. The public involvement impact assessment framework guidance. *Universities of Lancaster, Liverpool and Exeter* 2014

For peer review only - http://bmjopen.bmj.com/site/about/guidelines.xhtml

128. Modjarrad K, Moorthy VS, Millett P, et al. Developing Global Norms for Sharing Data and Results during Public Health Emergencies. *PLOS Medicine* 2016;13(1):e1001935. doi: 10.1371/journal.pmed.1001935
129. Menikoff J. Letter from Jerry Menikoff, MD, JD, Director, Office for Human Research Protections, to ICMJE Secretariat. 7 March 2017, 2017.

For peer review only

For peer review only - http://bmjopen.bmj.com/site/about/guidelines.xhtml

Supplementary Table 1. List of longitudinal research studies and active surveillance programs that have agreed to contribute participant-level data to the ZIKV Consortium individual participant data meta-analysis of longitudinal studies of pregnant women and their infants and children

Country	City	Study Name	Coordinating Center(s)	Consortium Name*
Brazil	Campina Grande	Freqüência e evolução dos achados ultrassonograficos e de ressonäncia magnética em fetos de mães com sintomas de Zika virus e a associação com desfechos neonatais em Campina Grande - Paraíba: Estudo de coorte	Instituto do Cérebro, Rio de Janeiro; Instituto D´Or, Rio de Janeiro	
Brazil	Goiânia	Cohort of Pregnant women with rash from Goiânia, Goiás State, Brazil and Cohort of children vertically exposed to Zika virus in Goiania	Institute of Tropical Pathology and Public Health<br><br>Federal University of Goiás, Brazil	ZikaPLAN
Brazil	Jundiaí	Infecção Vertical pelo vírus ZIKA e suas repercussões na área materno-infantil	Faculdade de Medicina de Jundiaí	
Brazil	São Luís, Maranhão	Monitoramento da microcefalia em recém-nascidos e acompanhamento clínico e de crescimento e desenvolvimento de uma coorte de crianças com provável infecção congênita pelo virus da Zika	Hospital Universitário da Universidade Federal do Maranhão/HU/UFMA	

For peer review only - http://bmjopen.bmj.com/site/about/guidelines.xhtml

Country	City	Study Name	Coordinating Center(s)	Consortium Name*
Brazil	Metropolitan region of Recife, Pernambuco	Coorte de gestantes com exantema no estado de Pernambuco	Universidade Federal de Pernambuco and Centro de Pesquisas Aggeu Magalhães-Fiocruz-PE	MERG/Fiocruz, ZikaPLAN
Brazil	Pernambuco	Coorte de gestanes com exantema no estado de Pernambuco	Fundação Oswaldo Cruz (Fiocruz)	MERG/Fiocruz, ZikaPlan
Brazil	Pernambuco	Coorte clínica de crianças com microcefalia em Pernambuco	Universidade Federal de Pernambuco and Centro de Pesquisas Aggeu Magalhães-Fiocruz-PE	MERG/Fiocruz, ZikaPlan
Brazil	Ribeirão Preto	Natural history of Zika virus infection in pregnant and consequences for pregnancy, fetus and child (Zika Project in Pregnancy - ZIG)	Universidade de São Paulo	
Brazil	Rio de Janeiro	Infecção pelo vírus Zika em uma coorte de gestantes e seus conceptos	Maternidade Escola da Universidade Federal do Rio de Janeiro	
Brazil	Rio de Janeiro	Estudo de coorte de gestantes e criancas expostas e infectadas intrautero pelo Zika virus	Instituto de Puericultura e Pediatria Martagão Gesteira, Rio de Janeiro; Hospital Universitário Pedro Ernesto	
Brazil	Rio de Janeiro	Zika Virus Infection in Pregnant Women in Rio de Janeiro	Fundação Oswaldo Cruz (Fiocruz), Rio de Janeiro	Fiocruz
Brazil	Rio de Janeiro	Zika virus coinfection among HIV infected pregnant women in a Brazilian cohort	Hospital dos Servidores do Estado	

For peer review only - http://bmjopen.bmj.com/site/about/guidelines.xhtml

Country	City	Study Name	Coordinating Center(s)	Consortium Name*
Brazil	São José do Rio Preto	Diagnóstico de arboviroses brasileiras e emergentes em pacientes e mosquitos em duas regiões distintas do Brasil	Faculdade de Medicina de São José do Rio Preto, Secretaria de Desenvolvimento, Econômico, Ciência e Tecnologia, São Paulo State	
Brazil	Vitoria	Epidemia de Zika virus no estado do Espirito Santo: estudo de impacto da infeccao sobre o feto em uma coorte de gestantes, com sintomas da doenca e confirmacao virologica da infeccao	Hospital Universitário Cassiano Antônio de Moraes	
Brazil<br>Colombia<br>Guatemala<br>Nicaragua<br>Puerto Rico<br>Mexico		Zika in Infants and Pregnancy (ZIP)	RTI International; Eunice Kennedy Shriver National Institute of Child Health and Human Development; National Institute of Allergy and Infectious Disease, National Institute of Environmental Health Sciences; Fundação Oswaldo Cruz (Fiocruz)	NIH/NIAID
Colombia	Baranquilla, Soledad, Bucaramanga, Tuluá	Zika en Embarazadas y Niños (ZEN)		CDC/INS
Colombia	Santander	Neurodevelopment outcome of newborns exposed to Zika virus in utero (ZEN)	UNC-CH, Michigan State University, Universidad Industrial de Santander	

For peer review only - http://bmjopen.bmj.com/site/about/guidelines.xhtml

For peer review only

Country	City	Study Name	Coordinating Center(s)	Consortium Name*
Colombia	Barranquilla<br>Cali<br>Cúcuta	Vigilancia de Embarazadas con Zika (VEZ; Surveillance cohort)		CDC
Ecuador<br>Cuba<br>Mexico (IMSS, MOH)<br>Venezuela: Valencia<br>Brazil: Fortaleza, Recife, Rio de Janeiro<br>Colombia: Bucaramanga		Pregnant Women Cohort for evaluation of absolute and relative risk of congenital malformations after Zika virus infection – developmental milestones of children born to women exposed to Zika virus during pregnancy	Heidelberg University	ZIKAlliance, Fiocruz, IDAMS
Grenada		The Spectrum of Zika Disease in Grenada - Pregnancy Cohort	St. George's University, Stanford University, Windward Islands Research and Education Foundation	
Guadaloupe, Martinique, French Guyana, St Martin		Zika Virus Infection's Pregnancy Consequences in French Department of America (ZIKA-DFA-FE)		INSERM
French Guyana		Zika Virus Infection's Neonatal and Pediatric Consequences in French Department of America (ZIKA-DFA-BB)		INSERM
Honduras		Zika Virus Infection in Pregnant Women in Honduras (ZIPH case-cohort study)	Tulane	
La Réunion		ZikaRun: an integrative mother-infant inception cohort study to anticipate	Cellule Régionale de l'Institut de Veille Sanitaire océan Indien	INSERM

For peer review only - http://bmjopen.bmj.com/site/about/guidelines.xhtml

Country	City	Study Name	Coordinating Center(s)	Consortium Name*
		the introduction of Zika virus in the at-risk La Reunion island, Indian Ocean	2Département de Médecine Générale, UFR santé, Université de la Réunion, Saint Denis 3INSERM CIC1410, CHU Reunion, Saint Denis - Saint Pierre 4CH Gabriel Martin, Saint Paul 5Centre d'Etudes Périnatales de l'océan Indien (CEPOI), EA7388, Université de la Réunion, CHU Reunion, Saint Pierre 6UM 134 Processus Infectieux en Milieu Insulaire Tropical (PIMIT), Université de La Réunion, INSERM U1187, CNRS 9192, IRD 249 7UMR Diabète AthéroThRombose Océan Indien (DéTROI), INSERM U188, Sainte Clotilde, La Réunion"	
Jamaica, Haiti		ZIKAction: Mother to child transmission of Chikungunya, Dengue, and Zika Virus Infection: A prospective observational cohort study of pregnant women and their infants		ZIKAction

For peer review only - http://bmjopen.bmj.com/site/about/guidelines.xhtml

Country	City	Study Name	Coordinating Center(s)	Consortium Name*
Panama<br>El Salvador		Panama/El Salvador Influenza Birth Cohort Study with Added Zika Component		CDC
Spain		pedZIKARed/gestZIKARed Spanish Zika database for pregnant women and children	Barceola University Hospital Vall d'Hebron	ZIKAction
Suriname		A symptomatic cohort study in Zika infected pregnant women	Acadamic Hospital Paramaribo	
Western French Guiana		Association between Zika virus and foetopathy: a prospective cohort study in French Guiana	Centre Hospitalier de l'Ouest Guyanais Saint-Laurent du Maroni	

CDC=Centers for Disease Control and Prevention; IDAMS=International Research Consortium on Dengue Risk Assessment, Management, and Surveillance; INSERM=Institut National de la Santé Et de la Recherche Médicale; NIAID=National Institutes of Allergy and Infectious Disease; NIH=National Institutes of Health

For peer review only - http://bmjopen.bmj.com/site/about/guidelines.xhtml

Supplementary Table 2. PRISMA-P 2015 Checklist

This checklist has been adapted for use with protocol submissions to *Systematic Reviews* from Table 3 in Moher D et al: Preferred reporting items for systematic review and meta-analysis protocols (PRISMA-P) 2015 statement. *Systematic Reviews* 2015 **4**:1

Section/topic	#	Checklist item	Information reported		Page number(s)
			Yes	No	
**ADMINISTRATIVE INFORMATION**					
**Title**					
Identification	1a	Identify the report as a protocol of a systematic review	X		1
Update	1b	If the protocol is for an update of a previous systematic review, identify as such		X	
**Registration**	2	If registered, provide the name of the registry (e.g., PROSPERO) and registration number in the Abstract	X		1
**Authors**					
Contact	3a	Provide name, institutional affiliation, and e-mail address of all protocol authors; provide physical mailing address of corresponding author	X		1
Contributions	3b	Describe contributions of protocol authors and identify the guarantor of the review	X		26
**Amendments**	4	If the protocol represents an amendment of a previously completed or published protocol, identify as such and list changes; otherwise, state plan for documenting important protocol amendments		X	
**Support**					
Sources	5a	Indicate sources of financial or other support for the review	X		26
Sponsor	5b	Provide name for the review funder and/or sponsor	X		26
Role of sponsor/funder	5c	Describe roles of funder(s), sponsor(s), and/or institution(s), if any, in developing the protocol	X		26
**INTRODUCTION**					
**Rationale**	6	Describe the rationale for the review in the context of what is already known	X		9
**Objectives**	7	Provide an explicit statement of the question(s) the review will address with reference to participants, interventions, comparators, and outcomes (PICO)	X		12

For peer review only

BioMed Central The Open Access Publisher

Section/topic	#	Checklist item	Information reported		Page number(s)
			Yes	No	
**METHODS**					
**Eligibility criteria**	8	Specify the study characteristics (e.g., PICO, study design, setting, time frame) and report characteristics (e.g., years considered, language, publication status) to be used as criteria for eligibility for the review	☒	☐	12
**Information sources**	9	Describe all intended information sources (e.g., electronic databases, contact with study authors, trial registers, or other grey literature sources) with planned dates of coverage	☒	☐	13
**Search strategy**	10	Present draft of search strategy to be used for at least one electronic database, including planned limits, such that it could be repeated	☒	☐	13
STUDY RECORDS					
Data management	11a	Describe the mechanism(s) that will be used to manage records and data throughout the review	☒	☐	14
Selection process	11b	State the process that will be used for selecting studies (e.g., two independent reviewers) through each phase of the review (i.e., screening, eligibility, and inclusion in meta-analysis)	☒	☐	13
Data collection process	11c	Describe planned method of extracting data from reports (e.g., piloting forms, done independently, in duplicate), any processes for obtaining and confirming data from investigators	☒	☐	13
**Data items**	12	List and define all variables for which data will be sought (e.g., PICO items, funding sources), any pre-planned data assumptions and simplifications	☒	☐	14
**Outcomes and prioritization**	13	List and define all outcomes for which data will be sought, including prioritization of main and additional outcomes, with rationale	☒	☐	14
**Risk of bias in individual studies**	14	Describe anticipated methods for assessing risk of bias of individual studies, including whether this will be done at the outcome or study level, or both; state how this information will be used in data synthesis	☒	☐	17
DATA					
**Synthesis**	15a	Describe criteria under which study data will be quantitatively synthesized	☒	☐	17
	15b	If data are appropriate for quantitative synthesis, describe planned summary measures, methods of handling data, and methods of combining data from studies, including any planned exploration of consistency (e.g., I^2, Kendall's tau)	☒	☐	18
	15c	Describe any proposed additional analyses (e.g., sensitivity or subgroup analyses, meta-regression)	☒	☐	17-22
	15d	If quantitative synthesis is not appropriate, describe the type of summary planned	☐	☒	

BioMed Central
The Open Access Publisher

Section/topic	#	Checklist item	Information reported		Page number(s)
			Yes	No	
**Meta-bias(es)**	16	Specify any planned assessment of meta-bias(es) (e.g., publication bias across studies, selective reporting within studies)	☒	☐	22-24
**Confidence in cumulative evidence**	17	Describe how the strength of the body of evidence will be assessed (e.g., GRADE)	☒	☐	22

For peer review only

BioMed Central
The Open Access Publisher

Supplementary Table 3. Zika virus-related and general clinical trial databases (adapted from Reveiz, et al [1])

Data base name	Link
Clinical Trails.gov	https://clinicaltrials.gov/ct2/search
World Health Organization (WHO) International Clinical Trials Registry Platform (ICTRP)	http://apps.who.int/trialsearch/
United States Centers for Disease Control and Prevention (US-CDC)	https://www.cdc.gov/publications/
European Centers for Disease Control (E-CDC)	https://ecdc.europa.eu/en/publications-data
Pan American Health Organization (PAHO) Zika research portal	https://www.paho.org/zika-research/
Fiocruz Research portal	https://portal.fiocruz.br/
Sistema Nacional de Ética em Pesquisa (SISNEP)	http://portal2.saude.gov.br/sisnep/pesquisador/
Registro peruano de ensayos clínicos y de estudios observacionales (REPEC)	http://www.ensayosclinicos-repec.ins.gob.pe/acerca-del-repec/busqueda-de-ensayos-clinicos
Registro nacional de investigaciones en salud (ReNIS)	https://sisa.msal.gov.ar/sisa/#Renis
Registro nacional de ensayos clínicos (RNEC)	http://189.254.115.252/Resoluciones/Consultas/ConWebRegEnsayosClinicos.asp

Reference

1. Reveiz L, Haby MM, Martínez-Vega R, Pinzón-Flores CE, Elias V, Smith E, et al. Risk of bias and confounding of observational studies of Zika virus infection: A scoping review of research protocols. PLOS ONE. 2017;12(7):e0180220. doi: 10.1371/journal.pone.0180220.

For peer review only

Supplementary Text 1. ZIKV IPD-MA search strategy

PICO Question:

Population	Exposure	Comparator	Outcome (open)
Pregnant women and her fetus, infant, or child	ZIKV infection during pregnancy	No ZIKV infection during pregnancy	Primary: microcephaly, miscarriage, fetal loss. Secondary: early/late fetal death, ocular abnormalities, hearing loss, neuroimaging abnormalities, etc.

Medline (through Ovid):

1. exp Zika Virus Infection/ or exp ZIKA VIRUS/

2. (zika or ZIKV).ti,ab,kf.

3. 1 or 2

4. exp Pregnancy/ or exp Maternal Exposure/ or exp "Embryonic and Fetal Development"/ or exp "Congenital, Hereditary, and Neonatal Diseases and Abnormalities"/ or exp Infant/ or exp Child/

5. (pregnan* or matern* or gestation* or perinatal* or birth* or congenital* or newborn* or fetal or fetus* or foetal or foetus* or neonat* or infan* or toddler* or child*).ti,ab,kf.

6. 4 or 5

7. 3 and 6

8. 7 not (exp Animals/ not exp Humans/)

Embase (through Ovid):

1. exp Zika virus/ or exp Zika fever/

2. (zika or ZIKV).ti,ab,kw.

3. 1 or 2

4. exp pregnancy/ or exp pregnancy outcome/ or exp high risk pregnancy/ or exp pregnancy complication/ or exp maternal exposure/ or exp fetus/ or exp "functions of embryonic, fetal and placental structures"/ or exp Infant/ or exp infant disease/ or exp child/ or exp childhood disease/

5. (pregnan* or matern* or gestation* or perinatal* or birth* or congenital* or newborn* or fetal or fetus* or foetal or foetus* or neonat* or infan* or toddler* or child*).ti,ab,kw.

6. 4 or 5

7. 3 and 6

8. 7 not ((exp animal/ or exp nonhuman/) not exp human/)

For peer review only
